# A capability approach to assess aquaculture sustainability standard compliance

**DOI:** 10.1371/journal.pone.0227812

**Published:** 2020-01-23

**Authors:** Phatra Samerwong, Hilde M. Toonen, Peter Oosterveer, Simon R. Bush

**Affiliations:** Environmental Policy Group, Wageningen University, Wageningen, The Netherlands; Universitá Cattolica del Sacro Cuore, ITALY

## Abstract

Sustainability standards are used to assure improved environmental performance in the aquaculture sector. But standard setters face limitations in including a broad range of producers with different capabilities, which in turn reduces their scope and impact. Drawing on Sen’s capability approach, we introduce a novel way to assess the extent to which sustainability standards can support the capability of farmers to make prescribed improvements to their production practices. In doing so, we compare four shrimp aquaculture standards (Aquaculture Stewardship Council, Global Aquaculture Alliance, Southeast Asian Shrimp Aquaculture Improvement Protocol and the Thai Agricultural Standard) based on an analysis of what we label the ‘prescribed capitals’ and ‘bundle of capitals’ that underpin the compliance capability of producers. The results show that standards narrowly prescribe standards requiring human capital, while there is potential for prescribing a wider bundle of social, financial and physical capitals that can allow more flexible standard compliance. The findings raise the prospect of redesigning sustainability standards to support a broader diversity of producer capabilities and, in turn, increase their overall impact.

## Introduction

As in many food sectors, sustainability standards have emerged as a primary market-based assessment and assurance tool for ‘sustainable’ aquaculture production [1, 2]. There are currently more than 30 aquaculture standards available, ranging from certification schemes to recommendation lists, and representing a diverse set of claims related to food safety, quality, traceability, environmental and social impact [3]. What these standards hold in common is the prescription of principles, standards and criteria aimed at restructuring producer practices towards ‘improved’ forms of production [4].

The impact of sustainability standards depends to a large extent on the voluntary compliance of producers to their principles and indicators. This means that farmers are required to change their farming practices, farm management systems and/or shared water infrastructure between farms to meet the expectations prescribed within these principles and indicators [2, 5]. However, standard setters neither discriminate nor differentiate between producers based on their ability to make any improvements necessary for compliance. Instead the so-called ‘theory of change’ of these standards is based on the assumption that preferential market access will provide incentive enough for producers to invest in these improvements [6].

The assumption that market access provides adequate incentive for standard compliance is, however, questionable given that only between one and five percent of global aquaculture production is currently certified [7, 8]. In the shrimp aquaculture sector, for instance, the uptake of certification standards has been limited by the high diversity of production practices, location and farm size [9], as well as the differences in sectoral development and government capacities across different regions [10]. Another challenge is the presence of multiple standards, each requiring compliance to different standards with different ‘sustainability’ claims serving domestic and export markets. For example, producers in Thailand, a top five shrimp exporting country, have to comply with both national and international standards to meet the sustainability demands of buyers in the US and EU market [11]. For producers, non-compliance with these standards therefore means exclusion from these markets. For standard holders, the non-compliance of these producers limits the overall volume and area of production that meet their environmental and social goals, which in turn undermines the overall effectiveness of standards as a sustainability governance mechanism.

If sustainability standards are to increase the number of farms that are certified globally, we argue they need to move beyond a technical understanding of compliance to instead assess and support the capabilities of farmers to improve their production. ‘Technical compliance’ refers here to the voluntary performance of improved farming practices in response to standard criteria and their related indicators [12, 13]. Failure to comply with these technical criteria is generally thought to reflect the poor knowledge and/or skills of farmers, which is in turn commonly translated into the need for training programmes [14, 15]. Following the work of Sen [16, 17], a capabilities approach to standard compliance contrasts with this focus of ‘skills and knowledge’ by focusing instead on the socially mediated conditions that determine access and use of a portfolio of diversified ‘capitals’ (human, social, natural, physical and financial) by farmers that provide the means by which technical changes to their farming practices can be made (see also [18–20]). A capabilities approach also focuses attention on the degree of freedom farmers have to recombine different capitals to increase their capability to comply with standard requirements [21].

In this paper we develop a framework based on the capability approach of Sen to explore the extent to which aquaculture standards currently enable farmers to employ different capitals to comply with their technical requirements. Our analysis is divided in to two parts. First, based on the capability framework we analyse the extent to which four aquaculture sustainability standards (Aquaculture Stewardship Council, Global Aquaculture Alliance, Southeast Asian Shrimp Aquaculture Improvement Protocol and the Thai Agricultural Standard) enable producers to mobilise different combinations of capitals and therefore different capabilities for compliance. Second, we analyse how standard setters through their support programmes, designed to directly support producer compliance (see [22]), contribute to enhancing farmers’ capabilities. Our assumption is that by supporting a more diverse bundle of capitals standards and their support programmes will more effectively support farmers to achieve standard compliance; which can potentially increase the volume and area of certified production and improve the likelihood these standards will reach their environmental and social sustainability goals.

The following section presents the conceptual basis of the capabilities framework and its application to sustainability standards. We then outline the methodology used to operationalise the capabilities framework in our assessment of standards and their support programmes. The rest of the paper reports on the results of our analysis and discusses the potential contribution of the capabilities framework for reimaging the form and function of sustainability standards in the aquaculture sector and beyond.

## Capability assessment framework

### Capabilities, sustainability and standards

Sen’s work on capabilities has been interpreted, discussed and applied in different domains, ranging from economic development, social justice and environmental governance (e.g. [23–25]). Common to most if not all applications of a capabilities approach is to go beyond a simplistic view of cause and effect, and instead focus on the conditions which shape specific outcomes. For instance, instead of focusing on income as the cause of food insecurity, a capabilities approach draws attention to the wider social conditions that undermine a person’s ability to access and employ resources and skills to realize food security as an entitlement [24]. The same logic applies to studies on environmental governance. The negative consequences of environmental degradation is then not only determined by poor resource management, but by the inability of resource users to access and employ (for example) the legal rights needed to engage in effective resource management [26].

The notion of capabilities has been applied in certification to examine the extent that skills and knowledge are needed for standard compliance. For example, Lemeilleur’s analysis of mango farmers in Peru identified the threshold capital requirements smallholder producers must have to improve their production practices to meet standard requirements [27]. Studies applying Sen’s capabilities approach to standards are less common. Those that have used his approach have focused on notions of ‘rewarding regulation’, by analyzing how standards foster learning by producers and new connections with them and private and public actors who can improve their competitiveness [28]. Others have focused on the role of standards in affecting rights to resources needed to fulfil sustainability and humanitarian objectives [20]. None of these studies have, however, explored how the standards themselves limit or enable farmers to develop the capabilities to comply with their requirements. It is this gap in the literature we contribute to.

### Capabilities and capitals

Sen defines a capability as “the ability (a person has) to do (or be) certain things that she has reason to value” [17, p.1959]. Sen also argues that capabilities are not natural or intrinsic to an individual, or simply learnt. Capabilities are instead influenced by the social and political conditions within which an individual performs particular sets of ‘doings’ and ‘beings’ (which he labels ‘functionings’), such as shrimp farming or, more specific in the context of ‘sustainable’ or ‘responsible’ aquaculture, production practices compliant to requirements set by a standard. Improving the capabilities of individuals is then not only a matter of transferring the skills, knowledge or infrastructure to perform a given functioning (i.e. to comply), but instead the opportunities an individual has, or is provided, to acquire skills, knowledge or infrastructure necessary to perform the functioning [20, 29].

Sen further argues that the expansion of capabilities affords individuals the freedom to employ various combinations of performing everyday ‘doings’ and ‘beings’ [21, 30]. People might need or want to ‘do’ or ‘be’ different things, while still aiming to reach the same goal [19]. In the context of aquaculture, for instance, the more capabilities farmers have, the greater ability they have to not only meet basic needs and reproduce their practices, but to also actively engage in processes of change towards ‘improved’ or ‘better’ forms of production that can lead to wealth, wellbeing and/or sustainable production [31]. The potential of such expansion or diversification leads to increased freedom for farmers to make choices about how they improve their production.

The performance and diversification of capabilities, Sen argues, is based on the acquisition and translation of a set of assets or ‘capitals’ (also see [19, 32, 33]). Capitals are commonly categorised into five types: human, social, natural, physical and financial capital [33, 34]. Human capital refers to assets like knowledge, skills, health and labour. Social capital refers to for example informal networks, and membership of formalised groups or associations. Natural capital generally refers to natural resources (living and non-living), and/or to access rights to natural resources. Physical capital refers to basic infrastructure, like buildings, transportation, but also production technologies and tools. Financial capital refers to money and ‘savings’ (in various forms), as well as to access to financial services. Table 1 presents the five capitals and related capabilities, and how they enable a person to perform a functioning, or in other words, how capitals contribute to a producer’s ability to successfully perform sustainable aquaculture practices.

**Table 1:** Capitals and related capabilities.

Capital	Assets	Capability	Example
**Human Capital**	Knowledge, personal health, skills, labour, access to education and training	Ability to retrieve information, to understand, to reflect, and to physically carry out activities (e.g. to work)	An educated producer is more likely to correctly read drug prescriptions, thereby realizing the possibility of having a healthy stock
**Social Capital**	Social networks and informal relationships, memberships of formalized groups or associations	Ability to collaborate with, and learn from others, to engage in reciprocal interactions, to forge and maintain informal and formal relations	A producer connected to skilled/educated others (neighbours, co-producers, organization members) is more likely to easily ask for help and having broken tools or system errors fixed in a quick and cost-efficient manner
**Natural Capital**	Natural resources, both living and non-living (geology, land, soil, water, stocks, genetic resources), and/or rights to access to natural resources	Ability to situate one’s practices in an environment/ecosystem which provides necessary inputs for operations, and/or is insensitive (or little sensitive) to a farm’s waste outputs	A producer whose farm is located in an area with a year-round water supply of good quality is more likely to enable healthy stock growth
**Physical Capital**	Energy, irrigation and sanitation systems, buildings, transportation means and infrastructures, production technologies, and equipment	Ability to operate easily, efficiently and effectively or have infrastructures, systems or equipment in place for operating	A producer who has well-designed ponds or cages is more likely to avoid escapes
**Financial Capital**	Money and savings, access to loans, credits, financial services	Ability to purchase goods and services for production, to receive credit or make investments to sustain ongoing and future operations	A financially solvent producer is more likely to be able to do large or long-term investments in improving farm management

This typology of capitals is commonly used to assess the endowment of capitals available to an individual and the translation of these capitals into capabilities that enable them to achieve outcomes related to social wellbeing or environmental sustainability [31, 35]. In this paper we argue that the performance of primary production-related functionings, such as feeding or harvesting shrimp, are also dependent on the capitals available to an individual. As Sen also notes, however, the translation of individual capitals and the expression of individual capabilities is also affected by the social, political, economic conditions in which they exist (what Sen refers to as ‘conversion factors’) [19, 21, 36]. These enabling conditions influence both the capitals available to an individual as well as the freedom that individuals have to employ these capitals in developing the capability needed to achieve a goal like standard compliance [37, 38]. Conversely, as the rest of the paper argues, regulatory tools can also be seen and assessed as conditioning capabilities by enabling or limiting the freedom farmers have in meeting their requirements.

### Application to sustainability standards

The role of sustainability standards is to prescribe the performance of functions related to primary production [14]. For shrimp aquaculture, this includes setting and assessing technical criteria for on-farm practices and infrastructure related to pharmaceuticals use, feed, water management, labour and biodiversity (for further detail see [7]). However, included in these criteria are also prescriptions of the capitals needed for successful compliance–including the skills and knowledge farmers need to perform ‘sustainable’ or ‘responsible’ feeding or health management, or the infrastructure required for water management or biosecurity. Standards are, as such, not only prescriptions of ‘practice’, but also prescriptions of the capitals required to develop the capabilities to perform the practices needed to comply with standard requirements. To illustrate, in setting a specified survival rate as a measure of animal health, a standard may also require farmers to calculate survival rates from stocking to harvest. As such, standards not only expect information on a technical indicator to be reported, but also prescribes specific human capital (knowledge, skills) to meet those reporting requirements.

The prescription of capitals reflects a utilitarian logic of standards that assumes all farmers need the same capitals to achieve a standardised set of capabilities for compliance. But, as widely shown, this logic of standardisation tends to ignore the diversity of farmers, and the diverse ways of mobilizing skills, finance or social support to be certified (e.g. [39–41]). In short, the result of such a utilitarian logic is a narrow focus on specific capitals rather than the functioning they enable [42]. Furthermore, this logic fosters high levels of ‘specialisation’ and narrows the possible number of pathways to compliance down to those prescribed by standard setters [43, 44]. Such specialisation can also reduce the overall capability of farmers to respond to systemic issues like disease. But where farmers have greater freedom to diversify the capitals and therefore pathways to compliance they can also increase their capabilities for not only complying with a standard requirement but also more effectively dealing with the issues that negatively affect their production (see for e.g. [45, 46]).

**Fig 1:**
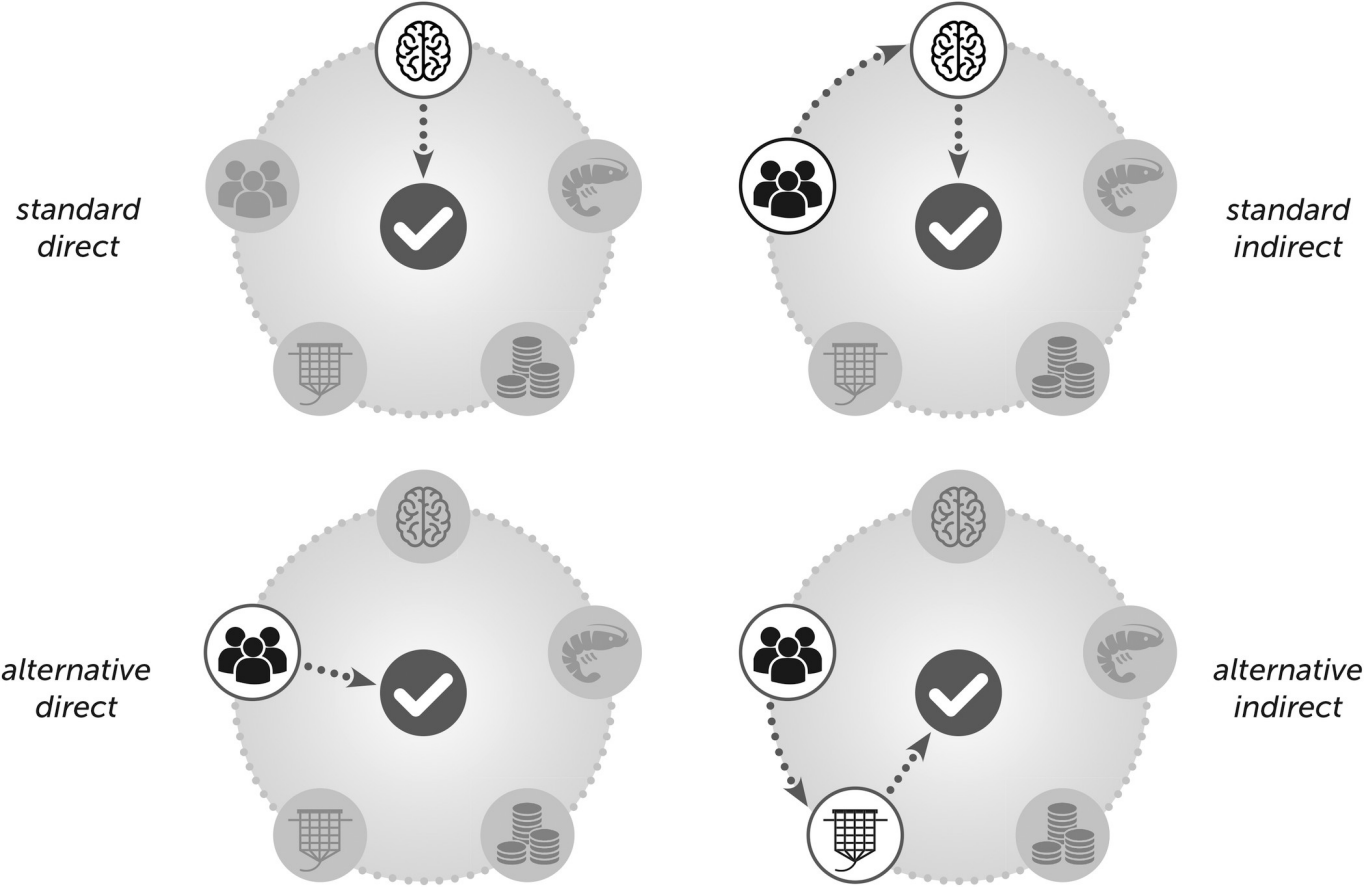
**Bundle of capitals** (1A: Capital directly prescribed by standard (standard direct); 1B: Alternative capital used to mobilize a capital prescribed by standard (standard indirect); 1C: Alternative (not prescribed) capital used to directly fulfil standard’s criteria (alternative direct); 1D: Alternative (not prescribed) capital used to mobilize another alternative (not prescribed) capital (alternative indirect).

Following Robeyns [19], we argue for a capabilities approach to standard design that focuses attention on the full ‘bundle’ of capitals that affect an individual’s freedom to develop compliance related functionings (see [47]). Underlying this approach is the assumption that different bundles of capitals can be used in different combinations to develop the capabilities required for standard compliance. For instance, a standard can prescribe that farmers use their own knowledge (human capital) to change farming practices in order to comply with the health management criteria of a standard (as illustrated in Fig 1A). Alternatively, if farmers have access to diverse bundle of capitals, they may also use their social capital to learn from others what knowledge and skills are needed in order to comply (Fig 1B). In doing so farmers use their bundle of capitals to indirectly mobilize the prescribed capital. But a farmer can also use their bundle of capitals to directly draw on social capital to bring in external knowledge to comply with a standard criteria (Fig 1C); or use social capital to change the physical capital (e.g. shared labour for the construction of a pond) such that the health criteria of a standard can also be met (Fig 1D). Conversely, if a farmer does not have the knowledge to comply, and only weak social or financial capital, the approach more specifically illuminates why they have limited options for achieving the functioning required for standard compliance.

## Methodology

### Standards assessed

For comparative purposes we limit our analysis to four standards that are all applied in a single country: Thailand. Each scheme holds different assumptions of what motivates farmers to improve their practices, either through market incentives or guided non-market improvement. However, the four standards are similar in that they are based on criteria covering a similar range of issues related to responsible or sustainable aquaculture production. The four standards, with a short description, are as follows:

1. *The Best Aquaculture Practices (BAP) Finfish and Crustacean Farm Standards*, *developed*, *and administrated by the Global Aquaculture Alliance (GAA)* [48]. BAP is a global third-party certification scheme, and its Finfish and Crustacean Farm standard is divided into 19 criteria, individually indicated as ‘critical’, ‘major’ or ‘minor’. The criteria are related to issues like community, environment, animal health and welfare, food safety, biosecurity and traceability. In total, the criteria cover 157 requirements.
2. *The Aquaculture Stewardship Council (ASC) Shrimp Standard* [49]. Like BAP, the ASC is also a global third-party certification scheme. The establishment of the ASC resulted from a series of Aquaculture Dialogues initiated by the World Wide Fund (WWF) and the Dutch Sustainable Trade Initiative (IDH). Its Shrimp Standard is based on seven principles, covering 110 metric-based indicators of farmer performance. A farm must achieve 100% compliance against all indicators in the ASC standard to be certified.
3. *The Southeast Asian Shrimp Aquaculture Improvement Protocol (SEASAIP) level one standard* set up by the Asian Seafood Improvement Collaborative (ASIC) [50]. ASIC is a regional multi-stakeholder arrangement, which has been funded by USAID and the Monterey Bay’s Seafood Watch programme. The SEASAIP level one standard has been designed to offer a roadmap for more inclusive improvement of shrimp farming. The standard is divided into eight principles related to production, environment and socio-economic criteria, which are developed based on the existing national standards the region with additional indicators of the Seafood Watch Aquaculture Sustainability criteria. Producers are required to demonstrate their compliance with all of the 78 criteria through a third party audit.
4. *The Thai Agricultural Standard Good Aquaculture Practices for Marine Shrimp Farms* (GAP-7401) [51]. GAP-7401 is a national voluntary public standard developed by the Thai government’s standard setting agency of National Bureau of Agricultural Commodity and Food Standards. The 70 requirements of this standard are organised around 10 ‘items’ related to farm management, including energy use, labour, social responsibilities and shrimp health management. The production practices are assessed against these requirements in an initial audit and subsequently re-audited if any improvements are deemed necessary [52].

### Analysis of standards

The analysis is based on the identification and comparison of 1) the ‘prescribed’ capitals set out by the standards, 2) the ‘bundles’ of capitals, which comprise both the prescribed capitals and the ‘alternative’ capitals which farmers could use to develop the capabilities required for complying to the requirements of each standard, and 3) the capitals addressed in the support programmes aimed to assist farmers to improve toward or meet the requirements of the standards. All standards require producers to comply with requirements either at the time of auditing or after the implementation of corrective actions. Therefore, our analysis does not suggest that the employment of different capitals allows farmers to influence audit scoring–e.g. trading off one area of assessment against another. Instead we assess how different capitals can be used to changing production practices and make material improvements to production systems that underpin the overall improvements necessary for standard compliance.

The identification and comparison of the prescribed capitals and the bundles of capitals are made from the perspective of the standards, by interpreting which capitals are explicitly or implicitly included in the different criteria [53]. Notably, we were unable to address how farmers interpret which capitals are relevant for improving their capability to comply given the scope and limited resources available for our analysis.

The analysis is carried out in three corresponding steps through mixed methods of analysing both quantitative and qualitative data.

First, ‘prescribed capitals’ were identified for every criteria of the four standards. The identification of these prescribed capitals was based on our explicit interpretation of the text and allocation of one or more of the capitals that relate to the resources, knowledge, skills, relations that enable producers to develop the capability for compliance. While the schemes mostly prescribed one capital to fulfil a requirement, all present instances of requirements in which more capitals have to be used (BAP: 10 requirements; ASC: 6; SEASAIP: 5; TAS 7401: 2). To ensure accuracy, the allocation of one or more of the five capitals was done iteratively, going back and forth between the capitals (with related capabilities need for compliance in mind), and the interpretation of the standard texts. This involved the lead author making an initial identification of capitals for each criteria and then testing this identification with the other authors.

Once the allocation of capitals was completed, the proportional distribution of prescribed capitals across all criteria for each standard was calculated.

This distribution indicates the relative importance of the different capitals for standard compliance. The more skewed the overall distribution to 1.0, the more compliance to the standard’s requirements depends on one capital, which in turn implies producers have limited degrees of freedom in how they can meet these requirements. Conversely, the closer the overall distribution of each capital to 0.2, the greater the degrees of ‘freedom’ (using Sen’s terms) a standard affords to a producer for developing the capabilities necessary for standard compliance. A distribution of, or close to, 0.2 reflects a more equally distributed set of prescribed capitals allowing farmers greater freedom to employ different capabilities and, as such, more diversified compliance strategies. Comparing the four standards allows us to identify the variation in how these schemes address producers’ capabilities.

Second, alternative capitals were identified, representing the potential alternative capitals that could be used to develop the capabilities necessary for standard compliance. The identification of the alternative capitals is based on the assumption that the capabilities needed for standard compliance can be realised through different combinations of the five capitals than those prescribed by the standards. These alternative capitals were identified by the authors by mapping out all the possible ways prescribed capitals could be substituted such that a producer could still develop the necessary capabilities to comply with each standard criteria. For example, a farmer can draw on social capital to meet a requirement for which human capital is prescribed, by asking either a friend or family member to provide the skills and knowledge necessary for meeting the requirements of a criteria. In such a case social capital represents one alternative pathway for a producer to develop the capability needed for compliance beyond the use of the prescribed capital.

The proportional distribution of the complete bundle of capitals, combining both the prescribed and alternative capitals, across all criteria was also calculated for each standard. Again, the closer to 1.0 the less degrees of freedom a standard confers to producer compliance, and the closer to 0.2 for each capital the greater the degrees of freedom. The difference between the proportional distribution of prescribed capitals and the bundle of capitals were also calculated, indicating the potential re-distribution and therefore change in the degrees of freedom that standards afford to producers in order to develop their capability for compliance. Also, the variation of the set of five capitals was calculated for each standard, both for the prescribed capitals and the bundles. This variation is measured by the standard deviation (SD), where a low SD indicates that combined the five capitals of a standard tend to be close to 0.2, so representing a larger degree of freedom.

### Analysis of support programmes

The final step of our analysis was to assess whether and how the pre-certification support programmes of each of the four standards relate to both the prescribed and alternative capitals.

Publicly available information (online sources) was reviewed [54–56] and semi-structured interviews were conducted in person and through Skype (between February 2017 and February 2018) with representatives from the four standard organisations and one official from the Thai Department of Fisheries. The interviews focused on the goal of the support programmes, and the kind of support activities they undertook with farmers. The specific questions asked related directly to the technical assistance, training, capacity building and network support provided by the programmes–with each question linking implicitly (not explicitly) to the five capitals. Based on these interviews we assessed the extent to which the goals and activities of the support programmes make use of the prescribed and alternative capitals identified in the analysis of the standards. This analysis assumed that programmes supporting the development of prescribed capitals stimulate specialised compliance that is achievable by a smaller range of farmers, while those supporting the development of alternative capitals are better able to incorporate diverse pathways for standard compliance that may in turn translate into higher levels of overall participation across the industry.

While systematic, the analysis of the standards and the support programmes remains interpretative [57]. To counterbalance any subjective bias in our interpretation the allocation normalization and aggregation steps of our analysis are made available in S1 to S4 Tables of the supplementary materials.

## Results

### Prescribed capitals

Our analysis of the prescribed capitals shows that the four schemes assume a very limited range of relevant capitals for developing the capabilities needed for standard compliance.

**Fig 2:**
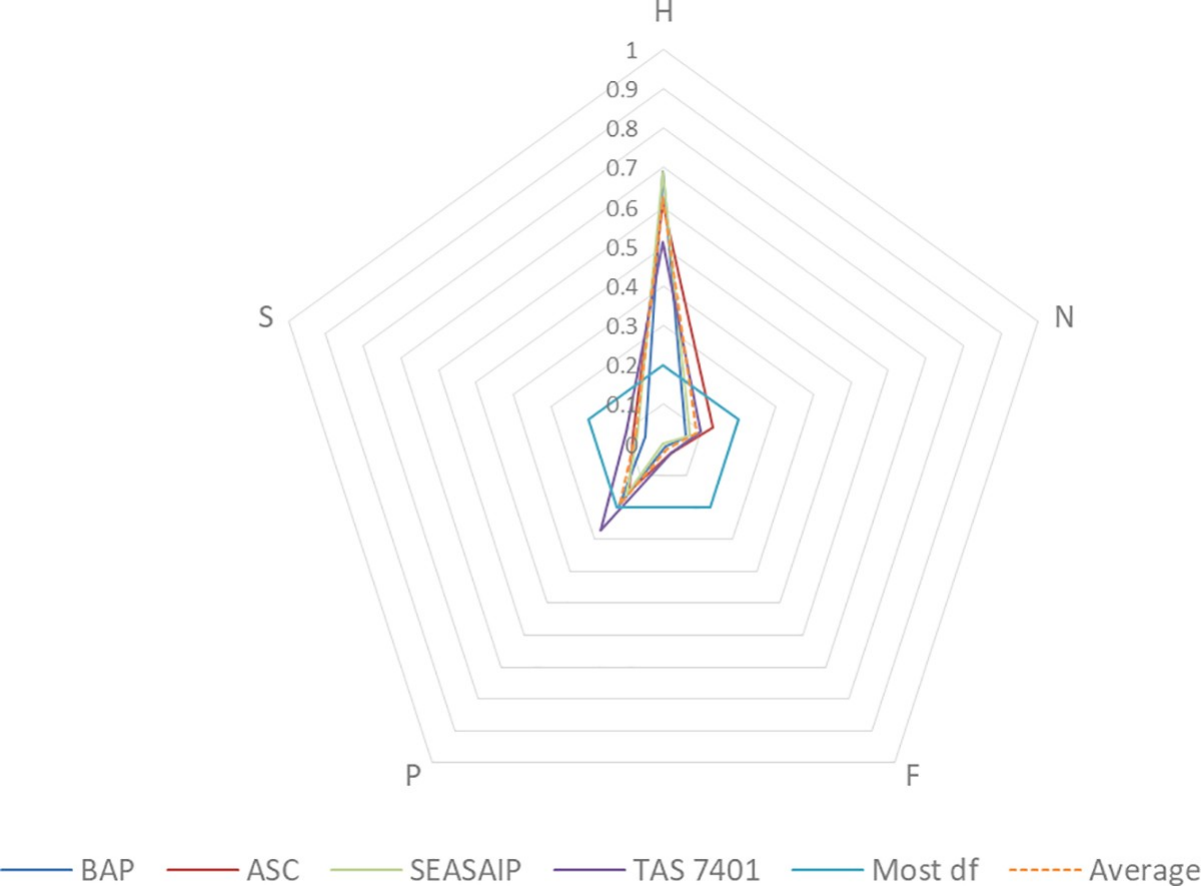
Comparison of prescribed capitals against a normalised capitals index across four aquaculture standards. (BAP—Best Aquaculture Practices; ASC—Aquaculture Stewardship Council; SEASAIP—Southeast Asian Aquaculture Improvement Protocol; TAS 7401—Thai Agricultural Standard).

Human capital is the most dominant capital across all four standards, with an average proportional distribution of 0.62 –larger than the all the other capitals combined (see Fig 2). The standard deviation (SD) between the standards is 0.08, indicating a relatively high degree of similarity between these standards with regard to human capital (see Table 2). The variation between the standards shows that BAP and SEASAIP have the highest relative reliance on human capital and the Thai TAS 7401 the least. Across all standards human capital is focused on 1) skills and knowledge on farming practices, 2) knowledge on regulations and compliant inputs (e.g. pharmaceuticals and feed), and 3) management skills, including data and documentation skills.

**Table 2:** Relative difference between prescribed capitals and bundles of capitals Standard.

	Capitals	H	S	N	P	F	SD
BAP	Prescribed	0.69	0.05	0.06	0.19	0.01	0.25
	Bundle	0.46	0.13	0.04	0.17	0.2	0.14
	Difference*	-0.23	0.08	-0.02	-0.02	0.19	0.14
ASC	Prescribed	0.61	0.08	0.13	0.15	0.03	0.21
	Bundle	0.47	0.08	0.1	0.16	0.19	0.14
	Difference*	-0.14	0	-0.03	0.01	0.16	0.1
SEASAIP	Prescribed	0.69	0.07	0.07	0.17	0	0.25
	Bundle	0.42	0.15	0.09	0.11	0.23	0.12
	Difference*	-0.27	0.08	0.02	-0.06	0.23	0.17
TAS 7401	Prescribed	0.50	0.1	0.1	0.27	0.03	0.17
	Bundle	0.36	0.12	0.08	0.2	0.24	0.10
	Difference*	-0.14	0.02	-0.02	-0.07	0.21	0.12
Average	Prescribed	0.62	0.07	0.09	0.20	0.02	0.22
	Bundle	0.43	0.12	0.08	0.16	0.21	0.12
	Difference*	-0.20	0.05	-0.01	-0.03	0.20	0.13
SD	Prescribed	0.08	0.02	0.03	0.05	0.01	Average 0.06
	Bundle	0.04	0.03	0.02	0.03	0.02	Average 0.03

* Difference between number of proportion of bundle of capitals increase or decrease from numbers of prescribed capitals

Physical capital, referring to changes in on-farm infrastructure (including ponds, equipment and feed), is ranked second across all standards with an average proportional distribution of 0.20. TAS 7401 places the greatest emphasis on physical capital compared with the other standards with a proportional distribution of 0.27. Overall, however, the SD in physical capital is low at 0.05, again indicating a relatively high degree of similarity between the standards.

The remaining prescribed capitals are far less prevalent than human and physical capital. Natural and social capital have an average proportional distribution of 0.09 and 0.07 respectively, with very low SD between the standards (0.03 and 0.02 respectively). Financial capital was the least prescribed capital with an average proportional distribution of 0.02 (and with a SD between the standards of 0.01). Notably, SEASAIP makes no prescription of financial capital at all, which appears to reflect its aim to reduce the financial burden for farmers when complying with their standard.

When combined, the prescribed capitals show that BAP and SEASAIP are more specialized, both have a SD of 0.25. TAS 7401 presents, on average, the most degrees of freedom for the prescribed capitals, while ASC falls in between (with a SD of 0.17 respectively 0.21).

### Bundles of capitals

The analysis of bundles of capitals shows that a more diverse set of capitals are possible for standard compliance than are currently prescribed. It is also apparent that these bundles, consisting of both prescribed and alternative capitals, offer a greater degree of freedom for developing the necessary capabilities for compliance than the prescribed capitals. The SD between capitals in these bundles is 0.03, which is half of the SD between prescribed capitals at 0.06. Of the four standards TAS 7401 has the most potential degree of freedom, with an SD between capitals of 0.1, followed by SEASAIP, with ASC exhibiting the same SD between capitals as BAP.

**Fig 3:**
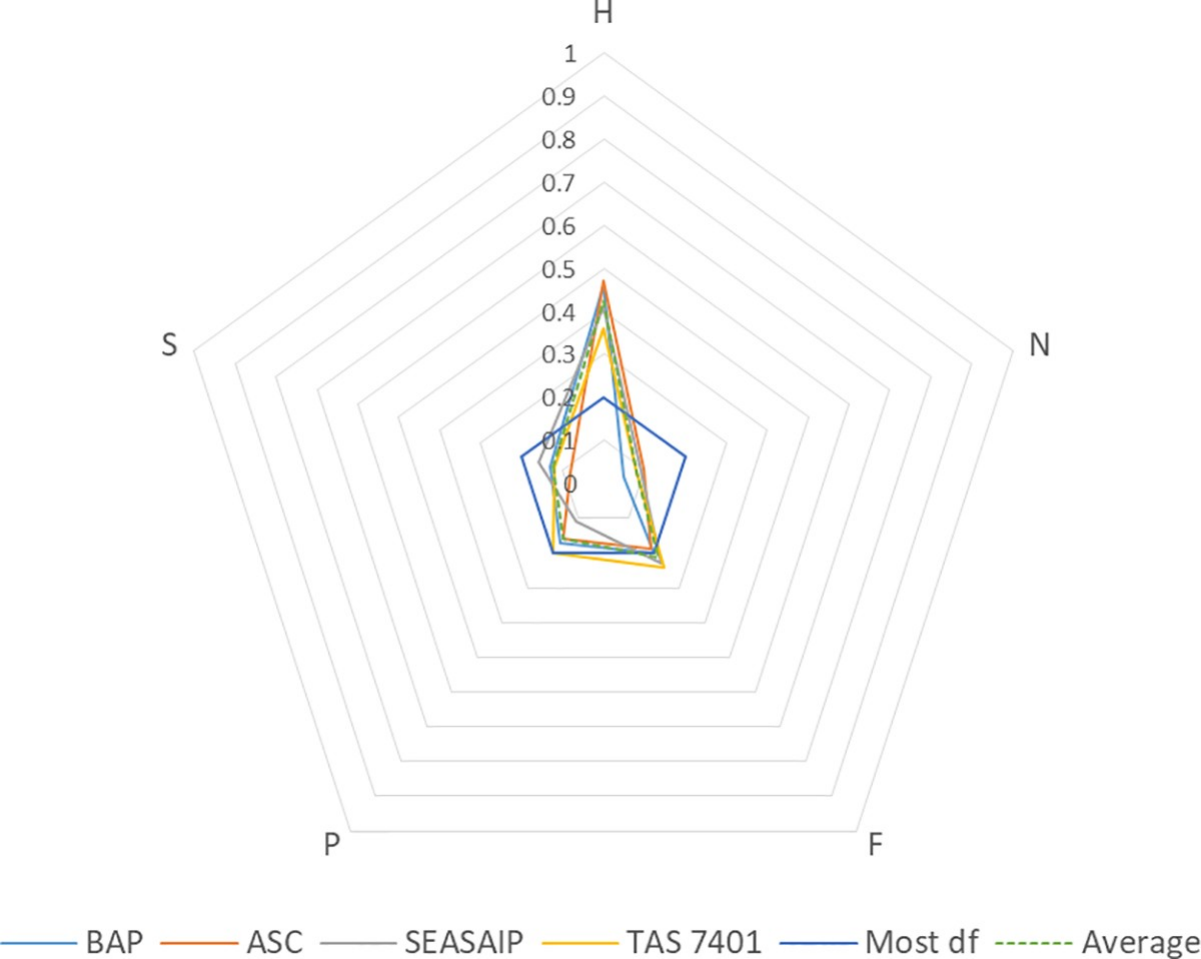
Comparison of bundle of capitals across against a normalised capitals index four aquaculture standards. (BAP—Best Aquaculture Practices; ASC—Aquaculture Stewardship Council; SEASAIP—Southeast Asian Aquaculture Improvement Protocol; TAS 7401—Thai Agricultural Standard).

Human capital remains the most dominant capital in the bundles of capitals across all standards with an average proportional distribution of 0.43, but less important (0.20 lower) than when it is a prescribed capital (see Fig 3). SEASAIP and BAP show the largest redistribution with a 0.27 and 0.23 shift from the prescription of human capital respectively. Correspondingly, the proportional distribution of financial capital increases by 0.20 from its prescribed level to become the second most important capital; indicating (as expected) that capitals are able to be monetised and exchanged. Social capital has smaller increases, ranging from 0.02 to 0.08, except for ASC where no change was observed. Physical capital is the third most important capital in the bundles overall, with a proportional distribution of 0.16, but decreased from its prescribed level in all standards, except for ASC. Natural capital remains the least important capital, with a proportional distribution of 0.08.

Overall, these results show that both financial and social capital present the most promising alternative means for farmers to develop the necessary capabilities for standard compliance. The increase in social capital in the bundles of capitals demonstrates the potential of social networks (and/or formal organisations such as shrimp clusters or cooperatives) to increase human capital by, for example, enabling shared learning of compliance related skills and knowledge. As expected, financial capital can enable producers to ‘buy in’ assistance, thereby bypassing their own lack of human capital, or invest in equipment or infrastructure. However, it is also noted that the combined proportional increase of social and financial capital indicates they may also be interlinked. As argued elsewhere (see [58, 59]), the ability to secure financial capital is largely dependent on the social relations of farmers, especially when they do not have access to formal sources of finance and credit.

**Fig 4:**
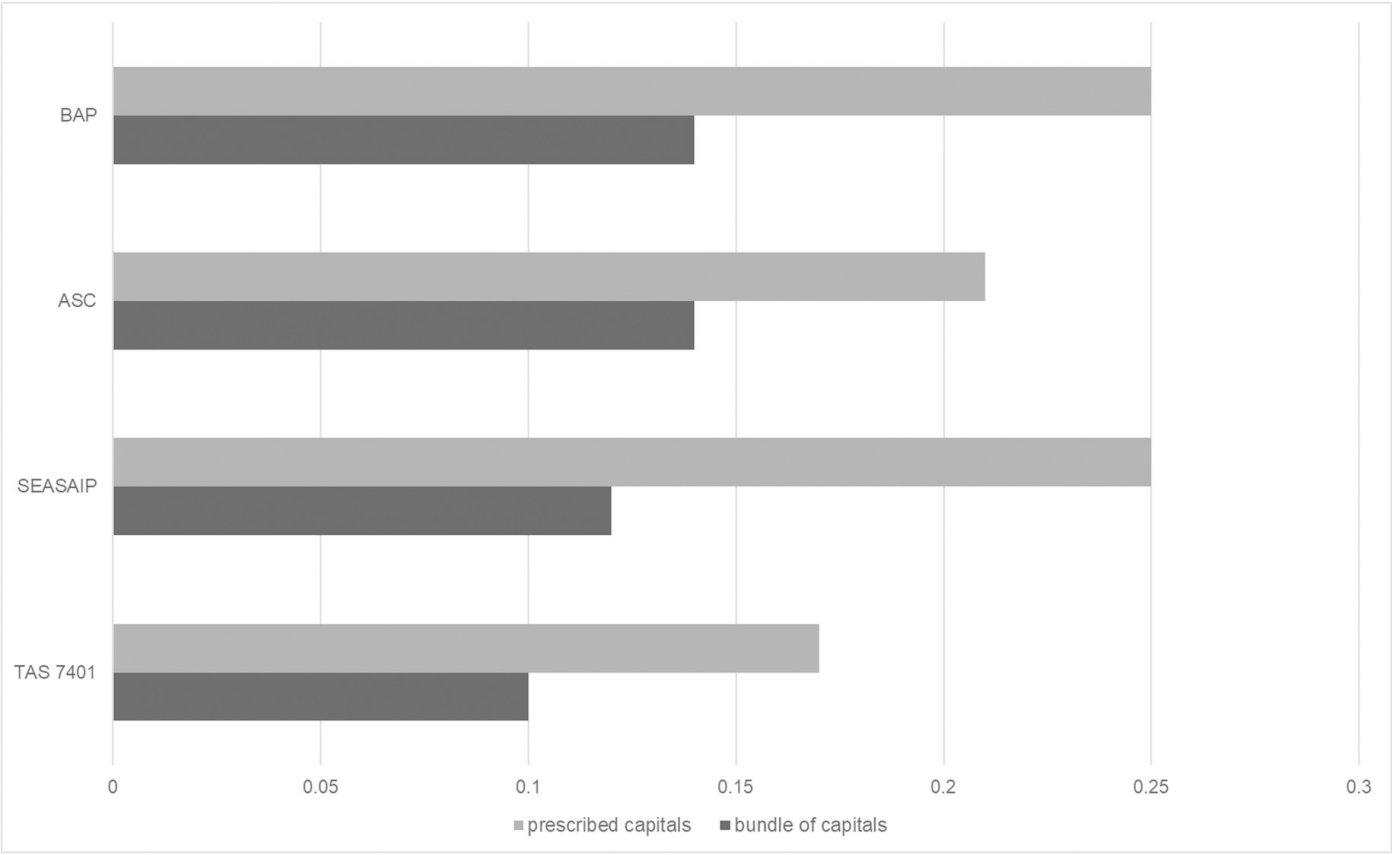
Comparison of average deviation between prescribed and bundles of capitals for four aquaculture standards. (BAP—Best Aquaculture Practices; ASC—Aquaculture Stewardship Council; SEASAIP—Southeast Asian Aquaculture Improvement Protocol; TAS 7401—Thai Agricultural Standard).

In comparing the standards, on average the bundles of capitals show that BAP and ASC remain relatively specialized, with a SD of 0.14 (see Fig 4). TAS 7401 has the lowest SD of 0.1, meaning that the capitals are more equally distributed, thereby providing higher degrees of freedom to farmers. Whilst SEASAIP represents the middle position, it does have the largest difference in SD between the prescribed capitals and the bundles of capitals.

### Certification support program

Our analysis indicates that the support programmes are strongly aligned with the prescribed capitals found in the standards. However, they also appear to be shifting their support beyond these prescribed capitals to a more diversified bundle of capitals (see Table 3). We observe this apparent shift in five ways.

**Table 3:** Certification support programs and supported capitals.

Standard	Indicators	BAP	ASC	SEASAIP	TAS 7401
**Human Capital**	Skills	✓	✓	✓	✓
	Knowledge	✓	✓	✓	✓
	Labour management	X	X	X	X
	Farming, pound management	✓	✓	✓	✓
	Documentation, Data collection	✓	X	o	✓
	General assessment	X	o	X	X
**Social Capital**	Knowledge sharing	✓	✓	✓	✓
	Connections with others	✓	✓	✓	✓
	Communication with community	✓	X	✓	✓
	Communication with authorities	X	X	X	✓
	Social network, connection with suppliers	✓	✓	✓	✓
	Participate in social organization, collective representation	✓	X	✓	✓
**Natural Capital**	Farm location	X	X	X	X
	Natural barriers	X	X	X	X
	Water, soil quality	X	X	X	X
	Specific shrimp larvae species	X	X	X	X
	Restoring the area	X	X	X	X
**Physical Capital**	Infrastructure	o	o	o	o
	Approved equipment, devices, materials	o	X	o	o
	Container, storage	X	X	X	X
	Approved probiotics	X	X	X	✓
	Specific feeds	o	o	o	o
	Irrigation, feeding system	X	X	X	X
**Financial Capital**	Purchasing certified, specific feeds	o	X	o	o
	Hiring assistant	X	X	X	X
	Assistance for construction	o	o	X	✓
	Buying proper equipment, supplies	o	o	X	✓
	Hiring expert to conduct assessment	X	X	X	X

Note: ✓ = Receives direct support on the capital, o = Receives indirect support through other capital, X = Does not receive support on the capital

First, representatives of the standard’s support programmes explicitly recognized the challenges farmers, who do requisite capabilities for compliance, face. They also acknowledged that, despite the often homogenising nature of the standard requirements, there is no archetypal farmer. Instead, the broad variation of ‘capitals’ held by farmers means there is a need for more flexible compliance strategies. For example, ASC expressed that they “should not only look at those producers who are close to ASC level […] but also those below the level for whom it is very difficult to move forward due to a lack of money or technical resources (sic.)”. Similarly, a SEASAIP representative argued that farmers who are “doing things differently” to what is prescribed by the standards, should also be given recognition because they might be moving towards the goals of standards. As summarised in Table 3, this translates into both direct support to prescribed capitals linked to the standard criteria and indirect support to alternative capitals that can also enable farmers’ capabilities to comply.

Second, the support programmes place a strong emphasis on human capital in terms of training, albeit through different channels. For example, the BAP representative explained that the iBAP programme bring in farms into BAP certification programme in “a more step-wise fashion” by providing step-by-step improvement plan and training through collaboration with processors involved in their programme [55]. ASC, while developing their own support programme, also collaborates with NGOs and processors to foster knowledge sharing in the form of deliver training or transition improvement programme (see S5 Table). By working with organisations beyond traditional extension services, BAP for instance collaborates with DoF in providing training through Seafood Taskforce, and ASC and TAS 7401 have partnered with organisations that are more connected to local farmers, such as WWF Thailand, local DoF and farmers cooperatives. These collaborations open up the possibility for different indirect channels of support for increasing the human capital of farmers required for compliance.

Third, support to human capital is closely related to support to social capital. All of the support programmes provide support to developing cooperative or group certification. Both the iBAP programme and ASC, for example, promote group certification in collaboration with their processors. BAP highlighted that "a more structured and formalized environment helps the group move along step-wise toward compliance”. SEASAIP and TAS 7401 engage farmers through a mix of NGOs, government extension and ‘pilot’ farmers. In all cases, the support programmes enrol farmers for training by a mix of peer-to-peer exchange, and by developing the capacity of groups to increase their economies of scale (for using compliant inputs and developing shared water infrastructure). Underlying these group activities is an implicit assumption that improved social capital will improve knowledge sharing for developing standard compliance capabilities, which in turn reduces the costs of certification.

Fourth, a mix of human and physical capital is observed through support given to what can be collectively labelled ‘information infrastructures’ by the support programmes. Most commonly this involves the development of reporting tools at the farm level and shared information systems at the cluster or group level. For example, SEASAIP attempted to develop a range of information technology systems for automatically measuring pond conditions which can feed into automated forms of compliance assessment. Integrating this with smartphone devices would allow to collect data directly from farms and cooperatives and digitalise them [60]. Similarly BAP intends to facilitate data collection with mobile devices. In all cases, the adoption of these ‘physical’ devices for data collection is aimed at supplementing the human capital (skills and knowledge) required for standard compliance.

Fifth, support to financial capital is provided across all support programmes, but often in combination with other capitals. The representative from BAP, for instance, stated that “there are actual investments to be made in farms, but farmers are either on their own or have processers as their sponsor”. Therefore, they focus on processors and collaborations with the Sustainable Trade Initiative (IDH) to provide financial support for changes identified in iBAP [55]. SEASAIP shared a similar vision, stating that financial support or an investment fund is needed so “farmers can tap into support and make improvements like infrastructure building”. For this, SEASAIP emphasized social capital, the partnership with supply chain actors, to share responsibility for certification and compliance costs. ASC and TAS 7401 also emphasize building social capital, by highlighting the need of in creating linkages to facilitate credits from existing financial institutions.

Overall, a wider set of capitals are taken up in the support programmes than observed in analysis of prescribed capitals, which focuses more narrowly on human capital. This indicates that certification schemes recognise the role that multiple capitals can play in support capabilities to ultimately comply with their standards.

## Discussion

The capabilities approach to aquaculture standards developed in this paper provides a new way of understanding producer compliance and improved production practices. Rather than evaluating compliance in terms of performing standard requirements, our approach focuses on the means by which producers can overcome limitations to and seek opportunities for improving their compliance capabilities.

Our findings have direct consequences for the content of standards and the conduct of their support programmes. Instead of (implicitly or explicitly) prescribing a narrow set of prescribed capitals for standard compliance, our analysis demonstrates there are bundles of capitals that farmers could draw upon for developing their compliance capabilities. While still exploratory, we also argue that if standards explicitly support the access or the development of alternative capitals they can in turn increase the potential number of producers who are able to respond to comply with their requirements. A capabilities approach could therefore enable standard organisations to respond to the key limitations they face in enabling producers, and especially small-holder producers [61] who are far below the level required for certification, to comply and be certified and, as such, increase their overall environmental and social impact.

More specifically, the results indicate that the over-reliance on human capital as a basis for compliance is too narrow. As presented in Table 2, all four standards analysed predominantly prescribe human capital as a means of standard compliance rather than social, financial, natural and physical capital. This in turn indicates a highly specialised and uniform mode of mobilizing capitals to meet their specific requirements. This specialised and uniform prescription of capitals can be considered an efficient way to improve production practices and comply with the standard (similar to the findings related to livelihoods of [62]). But specialisation also comes at a cost, as it reduces the degree of freedom that farmers have to employ their wider capabilities to develop the ‘functionings’ required for compliance (e.g. [46, 47]). Diversification through bundles of capitals, by contrast, can foster a higher degree of freedom that, when afforded to farmers, improves their capability to overcome limitations to standard compliance brought about by an over reliance on human capital alone. However, we also recognise that diversification can also come at a cost because it demands investments in capitals that may be ultimately redundant to compliance capabilities.

The results also reveal that, although largely unintentionally, all standards and their support programmes do currently allow for diversified bundles of capitals which can afford producers a greater degree of freedom in developing their compliance capabilities (cf. [63]). Human capital remains the most important capital for standard compliance in these alternative bundles. But, as we demonstrate, human capital can be replaced by social and financial capitals for most standard criteria–for example, through mobilizing skills and knowledge through collaboration and hired assistance. Key to this ‘substitution’ is the assumption that capitals are able to be converted from one capital into another capital (see [47, 64]). While we find that such conversion is possible in a large number of instances, it is also evident that not all capitals are equally convertible. For instance, while financial and social capitals provide farmers with more flexibility, natural and physical capitals (e.g. ponds, farm location, equipment) are not easily converted into other capitals. Furthermore, the conversion of capitals, regardless of the capability of a producer, is also influenced by wider social conditions beyond the control of standards and individuals. For example, rules and norms that structure access and control to natural resources such as land and water can either constrain or enable the extent to which capitals can be transformed or substituted [65].

Finally, our findings indicate that while standards continue to prescribe a narrow set of capitals they support a wider bundle of capitals in their support programmes. Again, while perhaps not intentional, the attention given to social, financial and physical capital (in addition to human capital) in these support programmes indicates a clear recognition of the breadth of the capabilities needed to comply with the standards. However, the ongoing mismatch between support programmes and prescriptive standards indicates that the standards are not yet fully aware of this potential. We see three opportunities for these support programmes to align their programmes with standards moving forward. First, they can explicitly identify the bundles of capitals, so both the prescriptive and alternative capitals, which producers need to improve their compliance with standard criteria and design their programme to support these capitals. Second, support programmes can attempt to change the social conditions that limit or enable producers to access their endowment of capitals or enable producers to convert one capital into another. Third, these programmes could support farmers to develop the capabilities needed to change the social conditions surrounding them that limit their ability to access or convert the capitals needed for improving the environmental and social performance of production and standard compliance.

## Conclusion

This paper presents demonstrates how the adoption of a capabilities approach can enable aquaculture standards to better support producer compliance by moving from a narrow focus on prescribed capitals to a more diversified bundle of both prescribed and alternative capitals. We argue that by supporting the development of these bundles of capitals producers are more likely to have greater freedom in developing the compliance capabilities that best suit the often dynamic social and environmental context in which they are embedded. Adopting a ‘bundled approach’ to developing producer capabilities means changes to the content of standard requirements as well as standard support programmes. In both instances attention needs to shift away from the skills and knowledge farmers need for compliance and instead focus on the social conditions that limit access to the capitals producers require for developing more adaptive compliance capabilities.

The results presented in this paper remain preliminary in that our analysis of both the standards and support programmes was not conducted in situ–that is, analysing the compliance challenges producers face given their local context. Further research is needed to further explore the potential of a capabilities approach to standard design. Particular attention should be given to explore which capitals producers use to respond to standard requirements in practice, as well as the social conditions that affect their ability to access and convert capabilities to develop compliance capabilities. Furthermore, attention could also be given to the practicalities of translating the capabilities approach presented into the re-design of standards and their support programmes. In doing so questions should further explore how the ‘theory of change’ of standards can be redesigned to more effectively foster greater progress towards more sustainable production.

## Supporting information

S1 Table Assessment of BAP. (PDF)

S2 Table Assessment of ASC. (PDF)

S3 Table Assessment of SEASAIP. (PDF)

S4 Table Assessment of TAS 7401. (PDF)

S5 Table Summary of certification support programs. (PDF)

## References

[1] Komives K, Jackson A. Introduction to Voluntary Sustainability Standard Systems In: Schmitz-Hoffmann C, Schmidt M, Hansmann B, Palekhov D, editors. Voluntary Standard Systems: A Contribution to Sustainable Development. Berlin, Heidelberg: Springer Berlin Heidelberg; 2014 p. 3–19.

[2] Tlusty MF, Tausig H. Reviewing GAA-BAP shrimp farm data to determine whether certification lessens environmental impacts. Reviews in Aquaculture. 2015;7(2):107–16. 10.1111/raq.12056

[3] Parkes G, Young JA, Walmsley SF, Abel R, Harman J, Horvat P, et al Behind the Signs—A Global Review of Fish Sustainability Information Schemes. Reviews in Fisheries Science. 2010;18(4):344–56. 10.1080/10641262.2010.516374

[4] Tlusty MF. Environmental improvement of seafood through certification and ecolabelling: theory and analysis. Fish and Fisheries. 2012;13(1):1–13. 10.1111/j.1467-2979.2011.00404.x

[5] Boyd CE, McNevin AA. Aquaculture, Resource Use, and the Environment: John Wiley & Sons, Inc.; 2014.

[6] Roheim CA, Bush SR, Asche F, Sanchirico JN, Uchida H. Evolution and future of the sustainable seafood market. Nature Sustainability. 2018;1(8):392–8. 10.1038/s41893-018-0115-z

[7] Potts J, MacFatridge S, Lynch M, Wilkings A. State of Sustainability Initiatives Review: Standards and the blue economy. International Institute for Sustainable Development, 2016.

[8] Bush SR, Toonen H, Oosterveer P, Mol APJ. The ‘devils triangle’ of MSC certification: Balancing credibility, accessibility and continuous improvement. Marine Policy. 2013;37:288–93. 10.1016/j.marpol.2012.05.011.

[9] Ashton E. The impact of shrimp farming on mangrove ecosystems2010.

[10] Miao W, Mohan CV, Ellis W, Davy B. Adoption of aquaculture assessment tools for improving the planning and management of aquaculture in Asia and the Pacific. Bangkok, Thailand: FAO Regional Office for Asia and the Pacific, 2013 Contract No.: RAP Publication 2013/11.

[11] Samerwong P, Bush SR, Oosterveer P. Implications of multiple national certification standards for Thai shrimp aquaculture. Aquaculture. 2018;493:319–27. 10.1016/j.aquaculture.2018.01.019.

[12] Amundsen VS, Gauteplass AÅ, Bailey JL. Level up or game over: the implications of levels of impact in certification schemes for salmon aquaculture. Aquaculture Economics & Management. 2019:1–17. 10.1080/13657305.2019.1632389

[13] Bosma R, Anh PT, Potting J. Life cycle assessment of intensive striped catfish farming in the Mekong Delta for screening hotspots as input to environmental policy and research agenda. The International Journal of Life Cycle Assessment. 2011;16(9):903 10.1007/s11367-011-0324-4

[14] Hatanaka M, Bain C, Busch L. Third-Party Certification in the Global Agrifood System. Food Policy. 2005;30:354–69. 10.1016/j.foodpol.2005.05.006

[15] Henson S, Jaffee S. Food Safety Standards and Trade: Enhancing Competitiveness and Avoiding Exclusion of Developing Countries. European Journal of Development Research. 2006;18 10.1080/09578810601070753

[16] Sen A. Capability and Well-Being In: Nussbaum M, Sen A, editors. The Quality of Life. Oxford: Clarendon Press; 1993.

[17] Sen A. Editorial: Human capital and human capability. Journal World Development. 1997;25(12):1959.

[18] Nussbaum MC. Aristotle, Politics, and Human Capabilities: A Response to Antony, Arneson, Charlesworth, and Mulgan. Ethics. 2000;111(1):102–40. 10.1086/233421

[19] Robeyns I. The Capability Approach: a theoretical survey. Journal of Human Development. 2005;6(1):93–117. 10.1080/146498805200034266

[20] Kalfagianni A. Addressing the Global Sustainability Challenge: The Potential and Pitfalls of Private Governance from the Perspective of Human Capabilities. Journal of Business Ethics. 2014;122(2):307–20. 10.1007/s10551-013-1747-6

[21] Sen A. Development as Freedom. Oxford: Oxford University Press; 1999.

[22] Bottema MJM. Institutionalizing area-level risk management: Limitations faced by the private sector in aquaculture improvement projects. Aquaculture. 2019;512:734310 10.1016/j.aquaculture.2019.734310.

[23] Walby S. Sen and the Measurement of Justice and Capabilities: A Problem in Theory and Practice. Theory, Culture & Society. 2012;29(1):99–118. 10.1177/0263276411423033

[24] Conceição P, Levine S, Lipton M, Warren-Rodríguez A. Toward a food secure future: Ensuring food security for sustainable human development in Sub-Saharan Africa. Food Policy. 2016;60:1–9. 10.1016/j.foodpol.2016.02.003.

[25] Burchi F, De Muro P. From food availability to nutritional capabilities: Advancing food security analysis. Food Policy. 2016;60:10–9. 10.1016/j.foodpol.2015.03.008.

[26] Bockstael E, Berkes F. Using the capability approach to analyze contemporary environmental governance challenges in Coastal Brazil. International Journal of the Commons. 2017;11:799–822. 10.18352/ijc.756

[27] Lemeilleur S. Smallholder Compliance with Private Standard Certification: The Case of GlobalGAP Adoption by Mango Producers in Peru. International Food and Agribusiness Management Review. 2012;16.

[28] Perez-Aleman P. Regulation in the Process of Building Capabilities: Strengthening Competitiveness While Improving Food Safety and Environmental Sustainability in Nicaragua*. Politics & Society. 2013;41(4):589–620. 10.1177/0032329213507553.

[29] Sen A. Commodities and Capabilities. UK: Oxford University Press; 1987.

[30] Sen A. Rationality and Freedom. Cambridge: The Harvard University Press; 2002.

[31] Bebbington A. Capitals and Capabilities: A framework for analysing peasant viability, rural livelihoods and poverty in the Andes. London: International Institute for Environment and Development (IIED), 1999 Contract No.: 6151IIED.

[32] Devereux S. Sen's Entitlement Approach: Critiques and Counter-critiques. Oxford Development Studies. 2001;29(3):245–63.

[33] Morse S, McNamara N. The Theory Behind the Sustainable Livelihood Approach. Sustainable Livelihood Approach: A Critique of Theory and Practice. Dordrecht: Springer Netherlands; 2013 p. 15–60.

[34] DFID. Sustainable Livelihoods Guidance Sheets. In: (DFID) DfID, editor. 1999. p. 149.

[35] Krantz L. The Sustainable Livelihoods Approach to Poverty Reduction: An Introduction. Stockholm: Sida, Division for Policyand Socio-Economic Analysis, 2001.

[36] Crocker DA. Ethics of Global Development: Agency, Capability, and Deliberative Democracy. Cambridge: Cambridge University Press; 2008.

[37] Evans P. Collective capabilities, culture, and Amartya Sen’s Development as Freedom. Studies in Comparative International Development. 2002;37(2):54–60. 10.1007/bf02686261

[38] Ibrahim SS. From Individual to Collective Capabilities: The Capability Approach as a Conceptual Framework for Self‐help. Journal of Human Development. 2006;7(3):397–416. 10.1080/14649880600815982

[39] Ouma S. Global Standards, Local Realities: Private Agrifood Governance and the Restructuring of the Kenyan Horticulture Industry. Economic Geography. 2010;86(2):197–222. 10.1111/j.1944-8287.2009.01065.x

[40] Oosterveer P, Adjei BE, Vellema S, Slingerland M. Global sustainability standards and food security: Exploring unintended effects of voluntary certification in palm oil. Global Food Security. 2014;3(3):220–6. 10.1016/j.gfs.2014.09.006.

[41] Henson S, Humphrey J. Understanding the Complexities of Private Standards in Global Agri-Food Chains as They Impact Developing Countries. The Journal of Development Studies. 2010;46(9):1628–46. 10.1080/00220381003706494 21328807

[42] Ortega Landa R. Amartya Sen: Utilitarianism, Ethics and Public Policy. Revista Latinoamericana de Desarrollo Económico. 2004:149–52.

[43] Emmanuelle C. Multi-stakeholder initiatives for sustainable agriculture: limits of the 'Inclusiveness' paradigm In: Stefano P, Peter G, Jakob V, editors. Governing through standards: origins, drivers and limitations. International Political Economy Series. Londres: Palgrave Macmillan; 2011 p. 210–35.

[44] Ponte S. ‘Roundtabling’ sustainability: Lessons from the biofuel industry. Geoforum. 2014;54:261–71. 10.1016/j.geoforum.2013.07.008.

[45] Whitney CK, Bennett NJ, Ban NC, Allison EH, Armitage D, Blythe JL, et al Adaptive capacity: from assessment to action in coastal social-ecological systems. Ecology and Society. 2017;22(2). 10.5751/ES-08983-220203

[46] Martin SM, Lorenzen K. Livelihood Diversification in Rural Laos. World Development. 2016;83:231–43. 10.1016/j.worlddev.2016.01.018.

[47] Ellis F. Rural livelihood diversity in developing countries: Evidence and policy implications. Natural Resource Perspectives. 1999;40(Livelihoods, diversification and Poverty).

[48] Global Aquaculture Alliance. Aquaculture Facility Certification Finfish and Crustacean Farms. Best Aquaculture Practices Certification Standards, Guidelines. New Hampshire, USA: Global Aquaculture Alliance (GAA); 2017.

[49] ASC. ASC Shrimp Standard v1.0—March 2014. Utrecht, The Netherlands: Aquaculture Stewardship Council (ASC); 2014.

[50] SEASAIP. The Southeast Asian Shrimp Aquaculture Improvement Protocol Draft #5. Southeast Asian Shrimp Aquaculture Improvement Protocol (SEASAIP); 2016.

[51] Thai Agricultural Standard TAS 7401–2014, TAS 7401–2014 (2014).

[52] Central Laboratory Thai. Inspection & Quality Systems and Products Certification. Inspection & Quality Systems and Products Certification: Central Laboratory (Thailand); 2018.

[53] Lebel L, Garden P, Luers A, Manuel-Navarrete D, Giap DH. Knowledge and innovation relationships in the shrimp industry in Thailand and Mexico. Proceedings of the National Academy of Sciences. 2016;113(17):4585–90. 10.1073/pnas.0900555106 19892739 PMC4855552

[54] Towers L. Indian, Thai Shrimp Farms Encouraged to Get BAP Certification: The FishSite; 2016 [updated February 15, 2016]. Available from: https://thefishsite.com/articles/indian-thai-shrimp-farms-encouraged-to-get-bap-certification.

[55] Best Aquaculture Practices. New iBAP Program To Advance Responsible Aquaculture 2015. Available from: https://bapcertification.org/blog/new-ibap-program-to-advance-responsible-aquaculture/.

[56] Peterson J. Best Aquaculture Practices Training in Malaysia. 2015.

[57] Booysen F. An Overview and Evaluation of Composite Indices of Development. Social Indicators Research. 2002;59(2):115–51. 10.1023/A:1016275505152

[58] Bush SR, Oosterveer P. The Missing Link: Intersecting Governance and Trade in the Space of Place and the Space of Flows. Sociologia Ruralis. 2007;47(4):384–99. 10.1111/j.1467-9523.2007.00441.x

[59] Kusumawati R, Bush SR, Visser LE. Can Patrons Be Bypassed? Frictions between Local and Global Regulatory Networks over Shrimp Aquaculture in East Kalimantan. Society & Natural Resources. 2013;26(8):898–911. 10.1080/08941920.2012.723305

[60] Bourgois E, editor Sustainability And Compliance Solutions For Asian Aquaculture Market/Value Chain: Introducing Verifik8. Asian-Pacific Aquaculture 2017; 2017; Kuala Lumpur, Malaysia.

[61] Marschke M, Wilkings A. Is certification a viable option for small producer fish farmers in the global south? Insights from Vietnam. Marine Policy. 2014;50:197–206. 10.1016/j.marpol.2014.06.010.

[62] Start D, Johnson C. Livelihood Options? The Political Economy of Access, Opportunity and Diversification 2004.

[63] Ellis F. Rural Livelihoods and Diversity in Devloping Countries. Oxford: Oxford university press Inc.; 2000.

[64] Bellwood-Howard I, Nchanji EB. The marketing of vegetables in a northern Ghanaian city: Implications and trajectories 2017 79–92 p.

[65] Stewart F, Deneulin S. Amartya Sen’s contribution to development thinking. Studies in Comparative International Development. 2002;37(2):61–70.

