# Supplementary material for: A capability approach to assess aquaculture sustainability standard compliance
Source: PLoS One. 2020 Jan 23;15(1):e0227812. doi: 10.1371/journal.pone.0227812 (PMC6977730; doi:10.1371/journal.pone.0227812)
Supplement: S1 Table — (PDF) [file pone.0227812.s001.pdf]

Supporting Information

The following supporting information presents data on the assessment of prescribed capitals and bundles of capitals for four standards:

1) The Best Aquaculture Practices (BAP) Finfish and Crustacean Farm Standards, developed by the Global Aquaculture Alliance (GAA) [1] (S1 Table)
2) The Aquaculture Stewardship Council (ASC) Shrimp Standard [2]. (S2 Table)
3) The Southeast Asian Shrimp Aquaculture Improvement Protocol (SEASAIP) level one standard [3] (S3 Table)
4) The Thai Agricultural Standard (TAS 7401) Good Aquaculture Practices for Marine Shrimp Farm, or alternatively named GAP-7401 [4] (S4 Table)
5) Summary of certification support programs (S5 Table).

S1 Table. Assessment of prescribed capitals and bundles of capitals for the Best Aquaculture Standards.

BAP	Prescribed capital	Justification	Bundle of capitals	In/Out	Justification (Bundle of capitals)
**1. Community (All Production Systems)**<br>**Property Rights and Regulatory Compliance**					
1.1: Current documents shall be available to prove legal land and water use by the applicant.	H	Documentation	H	In	
			S	Out	Criteron has no reference to social conditions
			N	Out	Criteron has no reference to natural conditions
			P	Out	Criteron has no reference to physical conditions
			F	Out	Criteron has no reference to financial conditions
1.2: Current documents shall be available to prove all business and operating licenses have been acquired.	H	Documentation	H	In	
			S	Out	Criteron has no reference to social conditions
			N	Out	Criteron has no reference to natural conditions
			P	Out	Criteron has no reference to physical conditions
			F	Out	Criteron has no reference to financial conditions
1.3: Current documents shall be available to prove compliance with applicable environmental regulations for construction and operation.	H	Documentation	H	In	
			S	Out	Criteron has no reference to social conditions
			N	Out	Criteron has no reference to natural conditions
			P	Out	Criteron has no reference to physical conditions
			F	Out	Criteron has no reference to financial conditions

**2. Community (All Production Systems)**<br>**Community Relations**					
2.1: The applicant shall accommodate local inhabitants by not blocking traditional access routes to fishing grounds, wetland areas and other public resources.	N	Farm site	H	In	Planning of farm layout
			S	Out	Criteron has no reference to social conditions
			N	In	
			P	In	Infrastructure
			F	In	Ability to relocate the farm
2.2: The applicant shall manage water usage to avoid restricting the amount of water available to other users.	H	Management	H	In	
			S	In	Communication, sharing water with neighbours and local community
			N	In	Availablity of water
			P	Out	Criteron has no reference to physical conditions
			F	Out	Criteron has no reference to financial conditions
2.3: The applicant shall demonstrate interaction with the local community to avoid or resolve conflicts through meetings, committees, correspondence, service projects or other activities performed annually or more often.	S	Communication with local community	H	In	Management
			S	In	
			N	Out	Criteron has no reference to natural conditions
			P	Out	Criteron has no reference to physical conditions
			F	Out	Criteron has no reference to financial conditions
**3. Community** (All Production Systems)<br>**Worker Safety and Employee Relations**					
**Wages and Benefits**					
3.1: The applicant shall meet or exceed the minimum wage rate, benefits, required by local and national labor laws.	H	Management	H	In	
			S	Out	Criteron has no reference to social conditions
			N	Out	Criteron has no reference to natural conditions

			P	Out	Criteron has no reference to physical conditions
			F	In	Ability to provide wage rate, benefits
3.2: The facility shall not make deductions from wages as part of a disciplinary process.	H	Management	H	In	
			S	Out	Criteron has no reference to social conditions
			N	Out	Criteron has no reference to natural conditions
			P	Out	Criteron has no reference to physical conditions
			F	Out	Criteron has no reference to financial conditions
3.3: The facility shall maintain all relevant documents that verify piece workers (those paid a fixed "piece rate" for each unit produced or action performed regardless of time) are paid in compliance with local law, including regulations regarding equivalence to or exceeding minimum requirements for wages, hours, overtime and holiday pay.	H	Documentation	H	In	
			S	Out	Criteron has no reference to social conditions
			N	Out	Criteron has no reference to natural conditions
			P	Out	Criteron has no reference to physical conditions
			F	Out	Criteron has no reference to financial conditions
**Working Hours**					
3.4: The applicant shall abide by the national mandated work week where applicable.	H	Management	H	In	
			S	Out	Criteron has no reference to social conditions
			N	Out	Criteron has no reference to natural conditions
			P	Out	Criteron has no reference to physical conditions
			F	Out	Criteron has no reference to financial conditions
3.5: The applicant shall comply with national labor laws for pay, overtime and holiday compensation for hours worked beyond the regular work day or week.	H	Management	H	In	
			S	Out	Criteron has no reference to social conditions
			N	Out	Criteron has no reference to natural conditions

			P	Out	Criteron has no reference to physical conditions
			F	Out	Criteron has no reference to financial conditions
**Forced, Bonded, Indentured, Trafficked, and Prison Labor**					
3.6: All work, including overtime, must be voluntary. The facility shall not engage in any form of forced or bonded labor. This includes human trafficking, the holding of original identity papers, prohibiting workers from leaving the premises after their shift or other coercion intended to force anyone to work. Where the holding of original identity papers is required by national law, such papers must be immediately returned to employees upon request and readily available to them at all times.	H	Management	H	In	
			S	Out	Criteron has no reference to social conditions
			N	Out	Criteron has no reference to natural conditions
			P	Out	Criteron has no reference to physical conditions
			F	Out	Criteron has no reference to financial conditions
3.7 The facility shall not require the payment of deposits, deduction from wages or withholding of pay that is not part of a legal contractual agreement with the employee and/or that is not provided for or permitted by national law.	H	Management	H	In	
			S	Out	Criteron has no reference to social conditions
			N	Out	Criteron has no reference to natural conditions
			P	Out	Criteron has no reference to physical conditions
			F	Out	Criteron has no reference to financial conditions
3.8: Workers shall have the right to terminate their employment after reasonable notice.	H	Management	H	In	
			S	Out	Criteron has no reference to social conditions
			N	Out	Criteron has no reference to natural conditions
			P	Out	Criteron has no reference to physical conditions
			F	Out	Criteron has no reference to financial conditions
**Child Labor and Young Workers**					

3.9: The applicant shall not engage in or support the use of child labor. The applicant shall comply with national child labor laws regarding minimum working age or ILO Minimum Age Convention 138, whichever is higher. ILO Minimum Age Convention 138 states the minimum age shall be 15, unless local law in developing nations is set at 14 – in accordance with developing nations exceptions under this convention.	H	Management	H	In	
			S	Out	Criteron has no reference to social conditions
			N	Out	Criteron has no reference to natural conditions
			P	Out	Criteron has no reference to physical conditions
			F	Out	Criteron has no reference to financial conditions
3.10: The employment of young workers above the minimum age but under 18 years old shall be in compliance with local laws, including required access to compulsory school attendance and any restrictions on hours and time of day.	H	Management	H	In	
			S	Out	Criteron has no reference to social conditions
			N	Out	Criteron has no reference to natural conditions
			P	Out	Criteron has no reference to physical conditions
			F	Out	Criteron has no reference to financial conditions
3.11: Young workers above the minimum age but under 18 years old shall not be subjected to hazardous work that can compromise their health and safety.	H	Labour management	H	In	
			S	Out	Criteron has no reference to social conditions
			N	Out	Criteron has no reference to natural conditions
			P	Out	Criteron has no reference to physical conditions
			F	Out	Criteron has no reference to financial conditions
3.12: The applicant shall only employ legally documented workers, whether nationals or migrants.	H	Labour management	H	In	
			S	Out	Criteron has no reference to social conditions
			N	Out	Criteron has no reference to natural conditions
			P	Out	Criteron has no reference to physical conditions

			F	Out	Criteron has no reference to financial conditions
3.13: The facility shall maintain all relevant documents that verify any contracted/subcontracted workers, whether contracted through a labor service or otherwise, are paid in compliance with all local wage, hour and overtime laws.	H	Documentation	H	In	
			S	Out	Criteron has no reference to social conditions
			N	Out	Criteron has no reference to natural conditions
			P	Out	Criteron has no reference to physical conditions
			F	Out	Criteron has no reference to financial conditions
3.14: All labor, recruiting or employment services used by the facility must be licensed to operate by the local or national government as a labor provider.	H	Labour management	H	In	
			S	Out	Criteron has no reference to social conditions
			N	Out	Criteron has no reference to natural conditions
			P	Out	Criteron has no reference to physical conditions
			F	Out	Criteron has no reference to financial conditions
3.15: The facility shall provide to all workers, whether hourly, salaried, piece-rate, temporary, seasonal or otherwise, prior to hire and during employment, written and understandable information regarding the terms of employment, worker rights, benefits, compensation, hours expected, details of wages for each pay period and facility policies regarding disciplinary actions, grievance procedures, authorized deductions from pay and similar labor-related issues. This information must be provided in the prevalent language of the majority of employees.	H	Labour management	H	In	
			S	In	Communication in local languages
			N	Out	Criteron has no reference to natural conditions
			P	Out	Criteron has no reference to physical conditions
			F	Out	Criteron has no reference to financial conditions
3.16: Where contracted/subcontracted or temporary workers are hired through a labor or employment service, the facility shall ensure	H	Labour management	H	In	
			S	In	Communication in local languages

that the labor or employment service provides the above information prior to and during hire, in appropriate languages, to ensure workers are aware of their rights and conditions of employment as described above.			N	Out	Criteron has no reference to natural conditions
			P	Out	Criteron has no reference to physical conditions
			F	Out	Criteron has no reference to financial conditions
3.17: The facility shall appoint a management person responsible for ensuring worker health, safety and training.	H	Management	H	In	
			S	In	Communications with workers, workers union
			N	Out	Criteron has no reference to natural conditions
			P	Out	Criteron has no reference to physical conditions
			F	Out	Criteron has no reference to financial conditions
3.18: The facility shall identify and eliminate or minimize any workplace health and safety hazards by conducting a thorough risk assessment. This includes a requirement for accident investigation.	H	Management	H	In	
			S	Out	Criteron has no reference to social conditions
			N	Out	Criteron has no reference to natural conditions
			P	Out	Criteron has no reference to physical conditions
			F	In	Hiring assistant to conduct risk assessment and minimize any hazards
3.19: If provided, employee housing shall meet local and national standards (e.g., water-tight structures, adequate space, heating/ ventilation/cooling), and shall be free of accumulated trash and garbage.	P	Infrastructure	H	Out	Criteron has no reference to human conditions
			S	Out	Criteron has no reference to social conditions
			N	Out	Criteron has no reference to natural conditions
			P	In	
			F	In	Building or renovate proper housing
3.20: Safe drinking water shall be readily available to employees. If meals are provided, they shall be wholesome and commensurate with local eating customs.	P	Infrastructure	H	In	
			S	In	
	H	Knowledge on wholesome meals	N	Out	Criteron has no reference to natural conditions

			P	In	
	S	Knowledge on local customs	F	In	Providing, buying safe drinking water for employees
3.21: Running water, toilets and hand-washing facilities shall be readily available to employees.	P	Infrastructure	H	Out	Criteron has no reference to human conditions
			S	Out	Criteron has no reference to social conditions
			N	Out	Criteron has no reference to natural conditions
			P	In	
			F	In	Provide, buy or install proper infrastructure or equipment
3.22: In the event of accidents or emergencies, the applicant shall provide basic medical care, including access to or communication with medical authorities. Additionally, first aid kits shall be readily available to employees, and any expired content shall be replaced.	H	Knowledge on basic medical care	H	In	
			S	In	
	P	Equipment	N	Out	Criteron has no reference to natural conditions
		Communication with authorities	P	In	
	S		F	In	Ability to buy first aid kits
3.23: The applicant shall provide training in general health, personal hygiene and safety (including aquatic safety and the use of boats and associated equipment), first aid and contamination risks to all employees. Safety documents must be available in a language understood by the workforce.	H	Training	H	In	
			S	In	Social networks facilitate knowledge sharing and translating documents into local language
			N	Out	Criteron has no reference to natural conditions
			P	Out	Criteron has no reference to physical conditions
			F	Out	Criteron has no reference to financial conditions
3.24: An emergency response plan shall be prepared for serious illnesses or accidents.	H	Management	H	In	
			S	In	Social networks facilitate knowledge sharing, learning from neighbour's example
			N	Out	Criteron has no reference to natural conditions
			P	Out	Criteron has no reference to physical conditions

			F	Out	Criteron has no reference to financial conditions
3.25: Select workers shall be made familiar with details in emergency response plans and trained in the first aid of electrical shock, profuse bleeding, drowning and other possible medical emergencies.	H	Management	H	In	
			S	Out	Criteron has no reference to social conditions
			N	Out	Criteron has no reference to natural conditions
			P	Out	Criteron has no reference to physical conditions
			F	Out	Criteron has no reference to financial conditions
3.26: Protective gear and equipment in good working order shall be provided for employees (e.g., eye protection for welding, gloves for shop work, boots for wet areas). Auditor to verify deployment.	P	Equipment	H	In	Maintenance
			S	Out	Criteron has no reference to social conditions
			N	Out	Criteron has no reference to natural conditions
			P	In	
			F	In	Ability to buy equipment
3.27: Electrical pumps and aerators shall be wired according to standard safe procedures. Machinery shall have proper driveshaft and/or drive belt safety guards.	P	Equipment	H	In	Proper installation, knowledge on policy
			S	In	Social networks, neighbours facilitate knowledge sharing and help in installation
			N	Out	Criteron has no reference to natural conditions
			P	In	
			F	In	Hiring assistant for proper installation
3.28: The applicant shall comply with laws that govern diving on aquaculture farms and develop a written dive safety plan that requires diver training and the maintenance of logs that document procedures, safety-related incidents and equipment maintenance. Limits for time under water shall be established and monitored.	H	Management, documentation	H	In	
			S	Out	Criteron has no reference to social conditions
			N	Out	Criteron has no reference to natural conditions
			P	Out	Criteron has no reference to physical conditions
			F	Out	Criteron has no reference to financial conditions
3.29: The applicant shall provide written procedures and staff training for handling	H	Management	H	In	
			S	Out	Criteron has no reference to social conditions

diving emergencies and regularly audit records and procedures. Emergency response equipment for divers shall include oxygen for resuscitation.	P	Equipment	N<br>P<br>F	Out<br>In<br>In	Criteron has no reference to natural conditions<br><br>Ability to buy emergency response equipment
3.30: The facility shall provide for equal opportunity with respect to recruitment, compensation, access to training, promotion, termination and retirement.	H	Management	H<br>S<br>N<br>P<br>F	In<br>Out<br>Out<br>Out<br>Out	 Criteron has no reference to social conditions<br>Criteron has no reference to natural conditions<br>Criteron has no reference to physical conditions<br>Criteron has no reference to financial conditions
3.31: The facility shall treat workers with respect and not engage in or permit physical, verbal or sexual abuse, bullying or harassment.	H	Management	H<br>S<br>N<br>P<br>F	In<br>Out<br>Out<br>Out<br>Out	 Criteron has no reference to social conditions<br>Criteron has no reference to natural conditions<br>Criteron has no reference to physical conditions<br>Criteron has no reference to financial conditions
3.32: Workers shall have the right to collective bargaining, or at least one employee shall be elected by the workers to represent them to management.	S	Collective representation<br>Worker organization	H<br>S<br>N<br>P<br>F	In<br>In<br>Out<br>Out<br>Out	Management<br><br>Criteron has no reference to natural conditions<br>Criteron has no reference to physical conditions<br>Criteron has no reference to financial conditions
3.33: There shall be a written worker grievance process, made available to all workers, that	H	Management	H<br>S	In<br>Out	 Criteron has no reference to social conditions

allows for the anonymous reporting of grievances to management without fear of retaliation.			N	Out	Criteron has no reference to natural conditions
			P	Out	Criteron has no reference to physical conditions
			F	Out	Criteron has no reference to financial conditions
**4. Environment (Ponds and Other Land-Based Systems Only)**<br>**Mangrove and Wetland Conservation**					
4.1: If net loss of wetland habitat (delineated by evaluation of hydrological conditions and the presence of wetland vegetation) occurred on facility property since 1999, the loss shall have been due to allowable purposes.	N		H	Out	Criteron has no reference to human conditions
			S	Out	Criteron has no reference to social conditions
			N	In	
			P	Out	Criteron has no reference to physical conditions
			F	Out	Criteron has no reference to financial conditions
4.2: If net loss of wetland habitat occurred on facility property since 1999, the loss shall have been mitigated by restoring an area three times as large or by an equivalent donation to restoration projects.	H	Restoring the area	H	In	
			S	In	Collective actions in restoring the area with other farmers
			N	In	Ability to find bigger area for restoring
			P	Out	Criteron has no reference to physical conditions
			F	In	Ability to donate for restoration projects
4.3: Farm activities shall not alter the hydrological conditions of the surrounding watershed, and the normal flow of brackish water to mangroves or freshwater to wetlands shall not be altered, unless specific permits apply.	N		H	In	Management of farm activities
			S	Out	Criteron has no reference to social conditions
			N	In	
			P	Out	Criteron has no reference to physical conditions
			F	Out	Criteron has no reference to financial conditions
	H	Maintainence	H	In	Maintainance

4.4: If wetland restoration has been conducted, the restored vegetation shall be maintained in a healthy state, viable and appropriately diverse.			S	In	Social networks facilitate or collective action with other farmers in maintain the area
			N	Out	Criteron has no reference to natural conditions
			P	Out	Criteron has no reference to physical conditions
			F	In	Hiring assistant to maintain the area
**5. Environment (Ponds and Other Land-Based Systems Only)**<br>**Effluent Management**					
5.1: If the facility is claiming the Limited Option as a justification for deviating from standard water quality criteria, it shall collect the requisite influent and effluent water quality data.	H	Data collection	H	In	
			S	Out	Criteron has no reference to social conditions
			N	Out	Criteron has no reference to natural conditions
			P	Out	Criteron has no reference to physical conditions
			F	Out	Criteron has no reference to financial conditions
5.2: If the applicant's facility operates within an irrigation system such that effluent water is exclusively destined to irrigate agricultural crops, Clauses 5.5 and 5.6 do not apply. Must be verified by auditor.	P	Irrigation system	H	Out	Criteron has no reference to human conditions
			S	Out	Criteron has no reference to social conditions
			N	Out	Criteron refers to natual conditions but focus in on irrigation system
			P	In	
			F	Out	Criteron has no reference to financial conditions
5.3: Clauses 5.5 and 5.6 do not apply for farms of less than 50 ha that avoid regular discharges of effluents into natural water bodies such that less than 1% of the culture water is exchanged daily on an annual basis – for example, by reusing all water or practicing infrequent, limited exchange of water.	N	Farm size	H	In	
			S	Out	Criteron has no reference to social conditions
	H	Management to avoid doing certian thing	N	In	
			P	Out	Criteron has no reference to physical conditions
			F	Out	Criteron has no reference to financial conditions

5.4: Records on volume of farm intake water use and results of effluent monitoring (if applicable) shall be maintained and available, as detailed in the Implementation Guidelines.	H	Documentation	H	In	
			S	Out	Criteron has no reference to social conditions
			N	Out	Criteron has no reference to natural conditions
			P	Out	Criteron has no reference to physical conditions
			F	In	Hiring assistant to monitor
5.5: Effluent water quality concentrations shall comply with BAP water quality criteria or applicable regulations if they are equivalent or more rigorous, or if this is not possible because of high concentrations in the intake water, concentrations shall reflect no deterioration between intake and discharge.	N	Water quality	H	In	Knowledge and calculation
			S	Out	Criteron has no reference to social conditions
			N	In	
			P	Out	Criteron has no reference to physical conditions
			F	Out	Criteron has no reference to financial conditions
5.6: Farms shall continue compliance with these criteria to maintain certification and comply with BAP's final criteria within five years.	H	Management	H	In	
			S	Out	Criteron has no reference to social conditions
			N	Out	Criteron has no reference to natural conditions
			P	Out	Criteron has no reference to physical conditions
			F	Out	Criteron has no reference to financial conditions
5.7: The farm shall provide the auditor with an estimated annual water use during the last calendar year, as illustrated in Appendix C, and the input data shall also be available for review.	H	Caculation	H	In	
			S	Out	Criteron has no reference to social conditions
			N	Out	Criteron has no reference to natural conditions
			P	Out	Criteron has no reference to physical conditions
			F	Out	Criteron has no reference to financial conditions
**6. Environment (Cages or Pens in Fresh or Brackish Water Only)** **Water Quality and Sediment Control**					

6.1: The water quality of the water body, including its discharge point if applicable, shall meet the BAP effluent water quality criteria, with sampling conducted following the implementation guidelines above.	N	Water quality	H	In	
			S	Out	Criteron has no reference to social conditions
	H	Sampling	N	In	
			P	Out	Criteron has no reference to physical conditions
			F	Out	Criteron has no reference to financial conditions
6.2: Facilities shall maintain accurate records of daily feed inputs that reflect compliance with the BAP maximum allowable daily feed input levels.	H	Caculation, documentation	H	In	
			S	Out	Criteron has no reference to social conditions
			N	Out	Criteron has no reference to natural conditions
			P	Out	Criteron has no reference to physical conditions
			F	In	Hiring assistant to maintain records
6.3: Total feed input for all culture operations on the lake or reservoir shall not exceed the BAP maximum allowable daily feed input.	P	Feeding system	H	In	Caculation
			S	Out	Criteron has no reference to social conditions
			N	Out	Criteron has no reference to natural conditions
			P	In	
			F	Out	Criteron has no reference to financial conditions
6.4: Water quality-monitoring records shall be applied in the management of feeding rates when dissolved oxygen levels are consistently below 5 mg/L in the early morning.	H	Monitoring	H	In	
			S	Out	Criteron has no reference to social conditions
			N	Out	Criteron has no reference to natural conditions
			P	Out	Criteron has no reference to physical conditions
			F	Out	Criteron has no reference to financial conditions
6.5: Water quality-monitoring records shall be applied in the management of feeding rates	H	Monitoring	H	In	
			S	Out	Criteron has no reference to social conditions

when mean annual Secchi disk visibility decreases by 25% since initial certification.			N	Out	Criteron has no reference to natural conditions
			P	Out	Criteron has no reference to physical conditions
			F	Out	Criteron has no reference to financial conditions
6.6: Water quality-monitoring records shall be applied in the management of feeding rates when blue-green or other potentially harmful algae comprise more than 60% of total phytoplankton.	H	Monitoring	H	In	
			S	Out	Criteron has no reference to social conditions
			N	Out	Criteron has no reference to natural conditions
			P	Out	Criteron has no reference to physical conditions
			F	Out	Criteron has no reference to financial conditions
6.7: Water quality-monitoring records shall be applied in the management of feeding rates when the thermocline becomes 25% shallower since initial certification.	H	Monitoring	H	In	
			S	Out	Criteron has no reference to social conditions
			N	Out	Criteron has no reference to natural conditions
			P	Out	Criteron has no reference to physical conditions
			F	Out	Criteron has no reference to financial conditions
6.8: For cages in water less than 30m deep where sediments are (in the absence of cages) usually aerobic, divers or cameras shall periodically, at least once per production cycle, inspect for accumulation of feces and uneaten feed and where necessary sites shall restore aerobic benthic conditions by fallowing or other means.	H	Inspection and restore aerobic benthic conditions	H	In	
			S	Out	Criteron has no reference to social conditions
			N	Out	Criteron has no reference to natural conditions
			P	In	Equipment (camera)
			F	In	Hiring assistant for inspection and restoring conditions
6.9: Monthly records of upstream and downstream total ammonia nitrogen and	H	Documentation	H	In	
			S	Out	Criteron has no reference to social conditions

soluble phosphorus concentrations shall be available.			N	Out	Criteron has no reference to natural conditions
			P	Out	Criteron has no reference to physical conditions
			F	Out	Hiring assistant to maintain record
6.10: Maximum daily feeding rates shall be managed such that downstream concentrations of nutrients do not exceed upstream concentrations by more than 25%.	H	Management	H	In	
			S	Out	Criteron has no reference to social conditions
			N	Out	Criteron has no reference to natural conditions
			P	In	Feeding system
			F	Out	Criteron has no reference to financial conditions
**Cages, Net Pens in Estuaries**					
6.11: Feeding records shall demonstrate that the maximum daily feeding rate of 7.5 kg/ha of estuary is not exceeded.	H	Documentation<br>Management	H	In	
			S	Out	Criteron has no reference to social conditions
			N	Out	Criteron has no reference to natural conditions
			P	Out	Criteron has no reference to physical conditions
			F	Out	Hiring assistant to manage feeding rate
6.12: Water quality-monitoring records shall be maintained as specified.	H	Documentation	H	In	
			S	Out	Criteron has no reference to social conditions
			N	Out	Criteron has no reference to natural conditions
			P	Out	Criteron has no reference to physcial conditions
			F	Out	Criteron has no reference to financial conditions
**7. Environment** (Marine Cages Only)<br>**Sediment Control**					
7.1: The applicant shall provide documents that describe local standards for benthic impacts	H	Documentation	H	In	
			S	Out	Criteron has no reference to social conditions

under cage farms, which shall include the benthic indicator "trigger level" above which the farm would not be in full compliance with the local standard, where this is clearly defined, or with its intent where it is not clearly defined.			N	Out	Criteron has no reference to natural conditions
			P	Out	
			F	In	Hiring assistant to prepare such documents
7.2: For established farms, the applicant shall provide three years of monitoring data to show that the farm meets or exceeds sediment quality criteria specified in its operating permits and/or its own monitoring plan at current operating levels.	H	Documentation	H	In	
			S	Out	Criteron has no reference to social conditions
			N	Out	Criteron has no reference to natural conditions
			P	Out	Criteron has no reference to physcial conditions
			F	Out	Criteron has no reference to financial conditions
7.3: For newly established farms or farms that have expanded and do not yet have enough monitoring data, the applicant shall provide an independent study that characterizes the hydrographic and benthic characteristics of the area and provides a consultant's opinion (without liability) that the farm can meet or exceed sediment and water quality criteria if operated correctly. This opinion shall be verified by reference to sampling results at the next audit.	F	Paid study by consultant	H	Out	Criteron has no reference to human conditions
			S	In	Social networks facilitate knowledge and study sharing of the area
			N	Out	Criteron has no reference to natural conditions
			P	Out	Criteron has no reference to physical conditions
			F	In	
7.4: Monitoring of sediment conditions shall be undertaken at the time of peak feeding during the production cycle and shall be conducted according to the requirements of the farm's operating permits or its own plan in countries or regions where sediment monitoring is not required, and as specified in the implementation requirements.	H	Monitoring	H	In	
			S	In	Social networks facilitate knowledge sharing and help
			N	Out	Criteron has no reference to natural conditions
			P	Out	Criteron has no reference to physical conditions
			F	In	Hiring assistant to monitor
7.5: Sediment sampling and analysis performed as part of the monitoring program shall apply	H	Monitoring	H	In	
			S	Out	Criteron has no reference to social conditions

generally accepted international methods and be adapted to the local hydrographic or benthic conditions.			N	Out	Criteron has no reference to natural conditions
			P	Out	Criteron has no reference to physical conditions
			F	In	Paid for lab testing
7.6: The results of sediment monitoring shall be reported to and reviewed and accepted by the appropriate regulators. Where regulatory approval is conditional upon implementing a program of remedial action, this shall have been implemented and completed.	H	Monitoring, documentation	H	In	
			S	In	Connection, communication with regulator for approval
			N	Out	Criteron has no reference to natural conditions
			P	Out	Criteron has no reference to physical conditions
			F	Out	Criteron has no reference to financial conditions
7.7: Data that will enable the farm's feed-based carbon and nitrogen discharge to be calculated shall be collected and recorded.	H	Monitoring, calculation	H	In	
			S	Out	Criteron has no reference to social conditions
			N	Out	Criteron has no reference to natural conditions
			P	Out	Criteron refers to physical conditions but focus is on data collection
			F	In	Hiring assistant to collect and record
7.8: Production cycles and fallowing shall be coordinated with other neighboring BAP applicants or BAP-certified farms, or with members of an established Area Management Agreement.	S		H	Out	Criteron has no reference to human conditions
			S	In	
			N	Out	Criteron has no reference to natural conditions
			P	Out	Criteron has no reference to physical conditions
			F	Out	Criteron has no reference to financial conditions
7.9: Where an AMA has not been established, applicants shall nevertheless demonstrate cooperation on matters of stocking, fallowing,	S		H	Out	Criteron has no reference to human conditions
			S	In	

animal health and biosecurity with BAP-certified farms within an area twice the regulatory minimum separation distance to an upper limit of a 5-km radius.			N	Out	Criteron has no reference to natural conditions
			P	Out	Criteron has no reference to physical conditions
			F	Out	Criteron has no reference to financial conditions
**8. Environment** (Ponds and Other Land-Based Systems Only)<br>**Soil and Water Conservation**<br>**Pond Sludge Management**					
8.1: If ponds are constructed on permeable soil, measures such as the use of pond liners shall be taken to control seepage and avoid contamination of aquifers, lakes, streams and other natural bodies of freshwater.	P		H	Out	Criteron has no reference to human conditions
			S	Out	Criteron has no reference to social conditions
			N	Out	Criteron has no reference to natural conditions
			P	In	
			F	In	Paid for pond liners construction
8.2: For inland brackish ponds, quarterly monitoring of neighboring well and surface water shall not show that chloride levels are increasing due to farm operations.	H	Monitoring	H	In	
			S	Out	Criteron has no reference to social conditions
			N	Out	Criteron has no reference to natural conditions
			P	Out	Criteron has no reference to physical conditions
			F	Out	Criteron has no reference to financial conditions
8.3: If a farm is extracting groundwater, water levels in nearby wells shall be monitored at least annually during the dry season to establish that aquaculture is not lowering the water table.	H	Monitoring	H	In	
			S	In	Social networks facilitate help in monitoring nearby wells
			N	Out	Criteron has no reference to natural conditions
			P	Out	Criteron has no reference to physical conditions
			F	Out	Criteron has no reference to financial conditions

8.4: Use of water from wells, lakes, streams, springs or other natural sources shall not cause ecological damage or subsidence in surrounding areas.	N		H	Out	Criteron has no reference to human conditions
			S	Out	Criteron has no reference to social conditions
			N	In	
			P	Out	Criteron has no reference to physical conditions
			F	Out	Criteron has no reference to financial conditions
8.5: Farm operations shall not cause wetland vegetation at the facility perimeter to die off.	N		H	Out	Criteron has no reference to human conditions
			S	Out	Criteron has no reference to social conditions
			N	In	
			P	Out	Criteron has no reference to physical conditions
			F	Out	Criteron has no reference to financial conditions
8.6: Dredge and fill activities shall not be conducted in sensitive wetlands or wetland buffers to increase the area available for pond construction.	H	Management to avoid certain thing	H	In	
			S	Out	Criteron has no reference to social conditions
			N	Out	Criteron has no reference to natural conditions
			P	Out	Criteron has no reference to physical conditions
			F	Out	Criteron has no reference to financial conditions
8.7: Any accumulated sludge removed from ponds, reservoirs or sedimentation basins shall be confined within the farm property or consolidated and used locally for landfill or agriculture.	P	Storage	H	In	Management
			S	Out	Criteron has no reference to social conditions
			N	Out	Criteron has no reference to natural conditions
			P	In	
			F	Out	Criteron has no reference to financial conditions
8.8: Removed sediment shall be properly contained and located to prevent the	P	Storage	H	Out	Criteron has no reference to human conditions

salinization of soil and groundwater and not cause other ecological nuisances.			S	Out	Criteron has no reference to social conditions
			N	Out	Criteron has no reference to natural conditions
			P	In	
			F	In	Ability to buy or build proper container
8.9: Facilities shall avoid the creation of degraded areas such as borrow pits and piles of soil.	H	Management	H	In	Criteron has no reference to human conditions
			S	Out	Criteron has no reference to social conditions
			N	In	Degraded soil
			P	Out	Criteron has no reference to physical conditions
			F	Out	Criteron has no reference to financial conditions
8.10: Dredged material shall be properly contained and not placed in mangrove areas or other sensitive habitats.	P	Storage	H	In	
			S	In	Social norm
	H	Management	N	Out	Criteron has no reference to natural conditions
			P	In	
			F	In	Ability to build proper storage
8.11: The applicant shall take measures to control erosion and other impacts caused by outfalls.	H	Management	H	In	
			S	Out	Criteron has no reference to social conditions
			N	Out	Criteron has no reference to natural conditions
			P	In	Proper equipment or infrastructure to control the erosion
			F	In	Ability to buy or build proper equipment of system
8.12: If the applicant's facility produces more than 20 MT/ha/crop, the facility shall possess sufficient sedimentation basin capacity to handle the associated sludge/sediment. The facility shall process all sludge/sediment in	P	Infrastructure	H	In	MAnagement
			S	In	Social norm
			N	Out	Criteron has no reference to natural conditions
			P	In	

sedimentation basins and not dump material in sensitive wetland or mangrove areas, or public water bodies.			F	In	Ability to buy or build proper equipment of system
**9. Environment (All Production Systems)**<br>**Fishmeal and Fish Oil Conservation**					
9.1: The applicant's facility shall use feed for which the manufacturer has provided data on the wild fishmeal and fish oil content or feed fish inclusion factor.	P	Feed	H	Out	Criteron has no reference to human conditions
			S	In	Connection with manufacturer that provide proper feed
			N	Out	Criteron has no reference to natural conditions
			P	Out	Criteron has no reference to physical conditions
			F	In	Ability to buy proper feed
9.2: The facility shall record the characteristics of all feeds used, the total amounts of each feed used each year and the total annual crustacean or fish production.	H	Documentation	H	In	
			S	Out	Criteron has no reference to social conditions
			N	Out	Criteron has no reference to natural conditions
			P	Out	Criteron has no reference to physical conditions
			F	In	Hiring assistant to record
9.3: The facility shall calculate and record a yearly feed-conversion ratio for completed crops.	H	Recording, caculating	H	In	
			S	Out	Criteron has no reference to social conditions
			N	Out	Criteron has no reference to natural conditions
			P	Out	Criteron has no reference to physical conditions
			F	In	Hiring assistant to record
9.4: The facility shall calculate and record a final yearly fish in:fish out ratio for completed crops.	H	Recording, caculating	H	In	
			S	Out	Criteron has no reference to social conditions

			N	Out	Criteron has no reference to natural conditions
			P	Out	Criteron has no reference to physical conditions
			F	In	Hiring assistant to record
9.5: The fish in:fish out ratio shall not exceed the following values: *Litopenaeus vannamei* – 1.2, *Penaeus monodon* – 1.7, tilapia – 0.7, *Pangasius* – 0.5. Limits have not yet been fixed for other species, and will be added once adequate data has been accumulated. For other species the values shall be recorded as information only.	N	Fish in: Fish Out (FIFO) ratios for the conversion of wild feed to farmed shrimp	H	In	Calculation, documentation
			S	Out	Criteron has no reference to social conditions
			N	In	
			P	Out	Criteron has no reference to physical conditions
			F	Out	Criteron has no reference to financial conditions
9.6: The applicant shall obtain feed from a BAP-certified feed mill or a feed mill that declares and documents compliance with standards 3.1 and 3.3 of the BAP feed mill standards.	S	Connection with manufacturer that provide proper feed	H	Out	Criteron has no reference to human conditions
			S	In	
			N	Out	Criteron has no reference to natural conditions
			P	Out	Criteron has no reference to physical conditions
			F	In	Ability to buy feed from certified sources
**10. Environment (All Production Systems)**<br>**Stocking Sources and GMOs**					
10.1: The facility shall maintain accurate records of the species farmed and, where relevant, any significant stock characteristics, including but not limited to non-native, specific pathogen-free, specific pathogen-resistant, hybrid, triploid, sex-reversed or genetically modified (GMO) status.	H	Documentation	H	In	
			S	Out	Criteron has no reference to social conditions
			N	Out	Criteron has no reference to natural conditions
			P	Out	Criteron has no reference to physical conditions
			F	Out	Criteron has no reference to financial conditions
10.2: If government regulations control the use or importation of any of the species or stocks	H	Documentation	H	In	
			S	Out	Criteron has no reference to social conditions

farmed, relevant permits shall be made available for inspection, even if imported fry were purchased from an intermediary.			N	Out	Criteron has no reference to natural conditions
			P	Out	Criteron has no reference to physical conditions
			F	Out	Criteron has no reference to financial conditions
10.3: The facility shall keep records of sources and purchases of stocking material, and record the number stocked in each culture unit for each crop.	H	Documentation	H	In	
			S	Out	Criteron has no reference to social conditions
			N	Out	Criteron has no reference to natural conditions
			P	Out	Criteron has no reference to physical conditions
			F	In	Hiring assistant to record
10.4: Wild juveniles shall not be stocked, other than as incidental introductions when extensive ponds are first filled.	H	Management	H	In	
			S	Out	Criteron has no reference to social conditions
			N	In	Ability to find other types of juveniles
			P	Out	Criteron has no reference to physical conditions
			F	In	Ability to buy other types of juveniles
10.5: Where the species farmed is not native, not feral or not already farmed, further documents shall be provided to demonstrate that regulatory approval for farming is based on the 2005 ICES Code of Practice on Introductions and Transfers of Marine Organisms or, for freshwater species, the Codes of Practice and Manual of Procedures for Consideration of Introduction and Transfers of Marine and Freshwater Organisms, FAO 1988.	H	Documentations	H	In	
			S	Out	Criteron has no reference to social conditions
			N	Out	Crtieron refers to natural conditions but focus is on the documentation
			P	Out	Criteron has no reference to physical conditions
			F	Out	Criteron has no reference to financial conditions
**11. Environment (All Production Systems, several sections for cages only)**<br>**Control of Escapes**					
11.1: All holding, transport and culture systems shall be designed, operated and maintained to	P	Infrastructure	H	In	Designing of infrastructure and farm layout, maintainence of the system

minimize the release of eggs, larval forms, juveniles and adult animals.			S	Out	Criteron has no reference to social conditions
			N	Out	Criteron has no reference to natural conditions
			P	In	
			F	In	Paid assistant to install and maintain the system
11.2: Screens and nets sized to retain the smallest farmed animals present shall be installed on water outlet pumps, pipes or sluices. Screens, nets or other controls shall be installed on or near pump intakes to minimize the introduction of local aquatic fauna.	P	Equipment	H	Out	Criteron has no reference to human conditions
			S	Out	Criteron has no reference to social conditions
			N	Out	Criteron has no reference to natural conditions
			P	In	
			F	In	Ability to buy the equipments
11.3: During harvesting and stock transfer operations, effective secondary containment measures shall be applied to control the escape of animals.	P	Equipment	H	In	Management
			S	Out	Criteron has no reference to social conditions
			N	Out	Criteron has no reference to natural conditions
			P	In	
			F	In	Ability to buy and install proper equipment and system
11.4: All incidents involving escapes of aquaculture animals shall be accurately documented.	H	Documentation	H	In	
			S	Out	Criteron has no reference to social conditions
			N	Out	Criteron has no reference to natural conditions
			P	Out	Criteron has no reference to physical conditions
			F	Out	Criteron has no reference to financial conditions
**12. Environment (All Production Systems, several standards for cages only)**<br>**Biodiversity and Wildlife Protection**					
12.1: The facility shall use humane methods of predator deterrents and actively favor non-	P	Humane and non-lethal equipments	H	In	
			S	Out	Criteron has no reference to social conditions

lethal methods. Where applicable, government permits for predator control shall be made available for review. No controls, other than non-lethal exclusion, shall be applied to species that are listed as endangered or highly endangered on the IUCN Red List or that are protected by local or national laws.	H	Documentation	N	Out	Criteron has no reference to natural conditions
			P	In	Equipments
			F	In	Ability to buy proper equipment
12.2: The facility shall record, and report where required, the species and numbers of all avian, mammalian and reptilian mortalities.	H		H	In	
			S	Out	Criteron has no reference to social conditions
			N	Out	Criteron has no reference to natural conditions
			P	Out	Criteron has no reference to physical conditions
			F	Out	Criteron has no reference to financial conditions
**13. Environment (All Production Systems)**					
13.1: Fuel, lubricants, feed and agricultural chemicals shall be labeled, stored, used and disposed of in a safe and responsible manner.	P	Infrastructure	H	In	Labelling, management
			S	Out	Criteron has no reference to social conditions
			N	Out	Criteron has no reference to natural conditions
			P	In	
			F	Out	Ability to provide proper storage and buy proper equipments
13.2: Fuel, lubricants and agricultural chemicals shall not be stored near feed, in employee housing or kitchen areas, or near harvest equipment and supplies.	P	Storage	H	Out	Criteron has no reference to human conditions
			S	Out	Criteron has no reference to social conditions
			N	Out	Criteron has no reference to natural conditions
			P	In	
			F	In	Ability to buid proper stroage
13.3: Fuel, lubricant and chemical storage areas shall be marked with warning signs.	H		H	In	
			S	Out	Criteron has no reference to social conditions

			N	Out	Criteron has no reference to natural conditions
			P	Out	Criteron refers to physical conditions but the focus is on proper signing
			F	Out	Criteron has no reference to financial conditions
13.4: Precautions shall be taken to prevent spills, fires and explosions, and procedures and supplies shall be readily available to manage chemical and fuel spills or leaks. Designated staff shall be trained to manage such spills and leaks.	P<br>H	Infrastucture	H	In	
			S	Out	Criteron has no reference to social conditions
			N	Out	Criteron has no reference to natural conditions
			P	In	
			F	In	Ability to buy and prepare equipments
13.5: Garbage from housing and food waste shall be retained in water-tight receptacles with covers to protect contents from insects, rodents and other animals.	P	Storage	H	Out	Criteron has no reference to human conditions
			S	Out	Criteron has no reference to social conditions
			N	Out	Criteron has no reference to natural conditions
			P	In	
			F	Out	Ability to buy and install proper equipment for storage
13.6: Garbage and other solid waste, including fouling organisms, shall be disposed of to comply with local regulations and avoid environmental contamination and odor problems (e.g., recycling, burning, composting or placing in a legal landfill).	H	Management	H	In	
			S	In	Social norm
			N	Out	Criteron has no reference to natural conditions
			P	In	Reclycling infrastructure
			F	Out	Criteron has no reference to financial conditions
13.7: Household trash and other farm wastes shall not be dumped in mangrove areas, wetlands or other vacant land and shall be removed promptly and properly to avoid accumulation.	H	Management	H	In	
			S	In	Social norm
			N	Out	Criteron has no reference to natural conditions
			P	In	Infrastucture or system for proper waste discharging

			F	Out	Criteron has no reference to financial conditions
13.8: Discarded farm supplies and equipment (e.g., tires, pallets, bags, barrels, aeration paddles or engines) shall not be dumped in mangrove areas, wetlands or other vacant land, and shall be removed properly to avoid excessive accumulation.	H	Management	H	In	
			S	In	Social norm
			N	Out	Criteron has no reference to natural conditions
			P	In	Infrastucture or system for proper waste discharging
			F	Out	Criteron has no reference to financial conditions
13.9: Measures shall be taken to prevent infestation by animal and insect vectors and pests.	P	Equipment	H	In	Management
			S	Out	Criteron has no reference to social conditions
			N	Out	Criteron has no reference to natural conditions
			P	In	
			F	Out	Criteron has no reference to financial conditions
13.10: Secondary fuel containment shall conform to BAP guidelines for fuel storage.	P	Infrastucture	H	In	Management, knowledge on regulations
			S	Out	Criteron has no reference to social conditions
			N	Out	Criteron has no reference to natural conditions
			P	In	
			F	In	Ability to buy or provide proper containment
**14. Animal Health and Welfare (All Production Systems)**					
14.1: The applicant's facility shall apply a maximum biomass limit based on performance measures for aquatic animal health and survival records, and any applicable national regulations.	H	Knowledge	H	In	
			S	Out	Criteron has no reference to social conditions
			N	Out	Criteron has no reference to natural conditions
			P	Out	Criteron has no reference to physcial conditions
			F	Out	Criteron has no reference to financial conditions

14.2: Feeding shall be managed to avoid stress caused by under- or overfeeding.	H	Managment	H	In	
			S	Out	Criteron has no reference to social conditions
			N	Out	Criteron has no reference to natural conditions
			P	In	Feeding machine
			F	Out	Criteron has no reference to financial conditions
14.3: The facility shall define upper limits for time periods of fasting, crowding and time out of water to ensure best welfare practices and provide accurate records showing that these limits are respected.	H	Caculation, management	H	In	
			S	Out	Criteron has no reference to social conditions
			N	Out	Criteron has no reference to natural conditions
			P	In	Feeding machine
			F	Out	Criteron has no reference to financial conditions
14.4: Facility staff shall make regular inspections of the culture facility, water quality, and behavior and condition of crustaceans or fish.	H	Inspection	H	In	
			S	Out	Criteron has no reference to social conditions
			N	Out	Criteron has no reference to natural conditions
			P	Out	Criteron has no reference to physical conditions
			F	Out	Criteron has no reference to financial conditions
14.5: Disease outbreaks shall be managed through rapid diagnosis and treatment, and when necessary, humane slaughter.	H	Management	H	In	
			S	In	Social networks facilitate knowledge sharing and help
			N	Out	Criteron has no reference to natural conditions
			P	In	Equipment for treatment or slaughter
			F	Out	Criteron has no reference to financial conditions
	H		H	In	
			S	Out	Criteron has no reference to social conditions

14.6: Humane slaughter techniques shall be used that are appropriate for the culture species.			N	Out	Criteron has no reference to natural conditions
			P	In	Equipment
			F	Out	Criteron has no reference to financial conditions
14.7: When ill, deformed or unmarketable specimens are removed, they shall be documented and killed by humane techniques, with the carcasses disposed of responsibly in accordance with applicable local and state regulations.	H		H	In	
			S	Out	Criteron has no reference to social conditions
			N	Out	Criteron has no reference to natural conditions
			P	In	Proper slaughter equipment and proper containment for disposal
			F	Out	Criteron has no reference to financial conditions
14.8: Health management procedures shall be defined in a health management plan or operating manual, reviewed and approved by a fish health professional, that includes procedures to avoid the introduction of diseases, protocols for water quality management, health monitoring and disease diagnosis techniques.	H		H	In	
			S	In	
	S		N	Out	Criteron has no reference to natural conditions
			P	Out	Criteron has no reference to physical conditions
			F	In	Ability to pay for expert or professional help
14.9: The adequacy and duration of live haul transport methods shall be assessed through documented mortality rates during transport.	H	Documentations	H	In	
		Knowledge	S	Out	Criteron has no reference to social conditions
			N	Out	Criteron has no reference to natural conditions
			P	Out	Criteron has no reference to physical conditions
			F	Out	Ability to hire or paid to arrange for proper transport
**15. Food Safety (All Production Systems)**					
15.1: The facility shall conduct an assessment of the watershed surrounding the facility to identify any potential watershed contamination	F	Paid expert to conduct assessment	H	In	
			S	In	Social networks facilitate or collective action with othdr farmers to conduct assessment

risks. This includes monitoring any changes to land use practices over time. Potential watershed contamination risks may involve such things as pesticides, PCBs and heavy metals introduced from nearby industrial or agricultural operations.	H	Monitoring	N	Out	Criteron has no reference to natural conditions
			P	Out	Criteron has no reference to physical conditions
			F	In	
15.2: If used, drug treatments shall be based on recommendations and authorizations overseen by a fish health specialist only to treat diagnosed diseases, accompanied by antibiotic sensitivity testing in accordance with instructions on product labels and national regulations.	H	Managment	H	In	
			S	In	Social networks facilitate knowledge sharing by specialist
			N	Out	Criteron has no reference to natural conditions
			P	In	Proper drug treatments
			F	In	Ability to buy proper drug treatments
15.3: Records shall be maintained for every application of drugs and other chemicals that include the date, compound used, reason(s) for use, antibiotic sensitivity test results, dose and harvest date for treated production lots. See the Traceability requirement. Periodic verification testing of the effectiveness of the withdrawal period shall be conducted. The auditor shall collect usage data for all antimicrobials	H	Documentations	H	In	
			S	Out	Criteron has no reference to social conditions
			N	Out	Criteron has no reference to natural conditions
			P	Out	Criteron has no reference to physical conditions
			F	In	Hiring assistant to record
15.4: Any use of antifouling agents must involve recognized applications of approved materials in a manner that can be monitored for potential contamination of the aquacultured animals.	P	Approved materials	H	In	Knowledge on approved materials
			S	Out	Criteron has no reference to social conditions
			N	Out	Criteron has no reference to natural conditions
			P	In	
			F	In	Ability to buy proper approved materials
15.5: Any use of food additives shall involve approved materials and be monitored for time and method of application.	P	Approved materials	H	In	Knowledge on approved materials and monitoring
			S	Out	Criteron has no reference to social conditions
			N	Out	Criteron has no reference to natural conditions

			P	In	
			F	In	Ability to buy proper approved materials
15.6: Antibiotics or chemicals that are proactively prohibited in the producing or importing country shall not be used in feeds, pond additives or any other treatment.	H	Knowledge on what not to use	H	In	
			S	Out	Criteron has no reference to social conditions
			N	Out	Criteron has no reference to natural conditions
			P	In	Producers have other feeds that are not related to antibiotics
			F	Out	Criteron has no reference to financial conditions
15.7: Statements from fry, fingerling or postlarvae suppliers that declare no proactively prohibited drugs or other chemicals were applied to seed are required.	H	Documentations	H	In	
			S	In	Connection with suupliers to obtain proper documents
			N	Out	Criteron has no reference to natural conditions
			P	Out	Criteron has no reference to physical conditions
			F	Out	Criteron has no reference to financial conditions
15.8: For feed suppliers that are not BAP-certified, statements are required attesting to the application of production procedures that exclude proactively prohibited drugs, by-products from same species, unsafe levels of heavy metals and physical or other contaminants.	H	Documentations	H	In	
			S	In	Connection with suupliers to obtain proper documents
			N	Out	Criteron has no reference to natural conditions
			P	Out	Criteron has no reference to physical conditions
			F	Out	Criteron has no reference to financial conditions
15.9: Feed, nutritional supplements or pond additives used, manufactured, or prepared on the farm shall include procedures to ensure these substances do not contain unsafe levels of contaminants and contain only substances	H	Knowledge	H	In	
			S	In	Social networks, other farmers facilitate knowledge sharing and help
			N	Out	Criteron has no reference to natural conditions

permitted by the appropriate national authorities.			P	In	Producers have proper feed, nutritional supplements without these substances
			F	Out	Criteron has no reference to financial conditions
15.10: Antibiotics, antimicrobials or hormones shall not be used as growth promoters.	H	Knowledge	H	In	Knowledge on antibiotics
			S	Out	Criteron has no reference to social conditions
			N	Out	Criteron has no reference to natural conditions
			P	In	Producers have other growth promoters that are not related to antibiotics
			F	Out	Criteron has no reference to financial conditions
15.11 All records specified in Section 15 shall be retained for a period that exceeds both 12 months and the expected shelf life of the aquaculture products.	H	Documentations	H	In	
			S	Out	Criteron has no reference to social conditions
			N	Out	Criteron has no reference to natural conditions
			P	Out	Criteron has no reference to physical conditions
			F	In	Hiring assistant to record
**16. Food Safety (All Production Systems)**					
16.1: Domestic sewage shall be treated and properly disposed of to avoid contamination of surrounding areas (e.g., sewer system, septic system, portable toilet or outhouse).	P	Infrastructure	H	In	Management
			S	Out	Criteron has no reference to social conditions
			N	Out	Criteron has no reference to natural conditions
			P	In	
			F	In	Ability to buy and install proper equipment and system
16.2: Farm animals and domestic pets shall not be allowed to access production ponds.	P	Barrier	H	In	Management, human control
			S	Out	Criteron has no reference to social conditions
			N	Out	Criteron has no reference to natural conditions
			P	In	

			F	Out	Ability to install proper barrier
16.3: Human waste and untreated animal manure shall not be used to fertilize ponds.	H		H	In	
			S	Out	Criteron has no reference to social conditions
			N	Out	Criteron has no reference to natural conditions
			P	In	Ability to find and use other types of fertilize
			F	Out	Criteron has no reference to financial conditions
16.4: Uncooked organisms and their by-products shall not be used as feed in growout ponds.	H	Management	H	In	
			S	Out	Criteron has no reference to social conditions
			N	Out	Criteron has no reference to natural conditions
			P	In	Producers can use other types of feeds
			F	In	Ability to find and use proper feed
16.5: Fish and crustaceans shall be harvested and transported in a manner that maintains temperature control.	P	Infrastucture, transportation	H	In	Harvesting
			S	Out	Criteron has no reference to social conditions
			N	Out	Criteron has no reference to natural conditions
			P	In	
			F	In	Ability to buy and install proper storage
16.6: Ice shall be made from water that complies with microbial limits for potable water.	N		H	In	Knowledge onmicrobial limits for water
			S	Out	Criteron has no reference to social conditions
			N	In	
			P	Out	Criteron has no reference to physical conditions
			F	In	Ability to buy proper types of ice
16.7: Equipment and containers used to harvest and transport fish or crustaceans shall be cleaned, sanitized, and be free of lubricants, fuel, metal fragments and other foreign material.	P		H	In	Management
			S	Out	Criteron has no reference to social conditions
			N	Out	Criteron has no reference to natural conditions
			P	In	

			F	In	Ability to buy cleaning supplies, hiring assistant to clean
16.8: Non-approved chemicals shall not be applied directly or indirectly to aquacultured products during transport.	H	Knowledge on approved, non-approved chemicals, management	H	In	
			S	In	Social networks facilitate knowledge sharing on regulations on chemicals
			N	Out	Criteron has no reference to natural conditions
			P	In	Ability to have and use other approved chemicals
			F	Out	Criteron has no reference to financial conditions
16.9: Workers with wounds, open sores or skin infections shall be prohibited from handling harvested products.	H	Managment	H	In	
			S	Out	Criteron has no reference to social conditions
			N	Out	Criteron has no reference to natural conditions
			P	Out	Criteron has no reference to physical conditions
			F	Out	Criteron has no reference to financial conditions
16.10: Workers shall be trained in good hygienic practices to ensure they are aware of their roles and responsibilities for protecting aquaculture products from food safety risks such as contamination and deterioration.	H	Training	H	In	
			S	In	Social networks, or collective learning on hygienic practices
			N	Out	Criteron has no reference to natural conditions
			P	Out	Criteron has no reference to physical conditions
			F	Out	Criteron has no reference to financial conditions
**17. Biosecurity (All Production Systems)**					
17.1: The applicant shall have in place biosecurity controls that seek to prevent the introduction and spread of disease agents and disease on the farm, including the sanitization	P	Infrastructure	H	In	
			S	In	Social networks facilitate sharing help with neighbour or authorities or experts in case of suspected disease

of equipment and personnel when disease is suspected or confirmed at the farm site, and these shall be detailed in a biosecurity plan as described in the Implementation guidelines above.	H	Management	N	Out	Criteron has no reference to natural conditions
			P	In	
			F	In	Ability to buy and install proper equipment and system
17.2: Farm staff shall be trained in biosecurity procedures and shall, along with all visitors, comply with them.	H	Training	H	In	Management
			S	Out	Criteron has no reference to social conditions
			N	Out	Criteron has no reference to natural conditions
			P	Out	Criteron has no reference to physical conditions
			F	Out	Criteron has no reference to financial conditions
17.3: A plan for prompt and responsible disposal of excessive mortalities of culture animals by incineration, burial, composting or removal by a competent contractor shall be available for inspection and applied.	H	Management	H	In	
			S	Out	Criteron has no reference to social conditions
			N	Out	Criteron has no reference to natural conditions
			P	Out	Criteron has no reference to physical conditions
			F	Out	Criteron has no reference to financial conditions
17.4: Where slaughtering is conducted at the farm, blood water and other effluents generated through processing shall be contained or treated so they do not contaminate the environment or present a biosecurity risk.	P	Equipment	H	Out	Criteron has no reference to human conditions
			S	Out	Criteron has no reference to social conditions
			N	Out	Criteron has no reference to natural conditions
			P	In	
			F	In	Ability to buy proper equipment
**18. Traceability (All Production Systems)**					
18.1: The facility shall operate an effective record-keeping system that provides timely, organized, accurate entries, performed and overseen by a designated trained person or	H	Recording	H	In	
			S	In	Social networks facilitate sharing help with cooperatives, team leader, learning from others

team responsible for collecting the data, ensuring it is complete and accurate, and that traceability requirements are met.			N	Out	Criteron has no reference to natural conditions
			P	Out	Criteron has no reference to physical conditions
			F	In	Hiring assistant to record
18.2: The facility shall keep complete and accurate records for each culture unit and production cycle, including the culture unit identification number, unit area and volume, species stocked and, if applicable, species specifications such as triploid or GMO.	H	Recording	H	In	
			S	Out	Criteron has no reference to social conditions
			N	Out	Criteron has no reference to natural conditions
			P	Out	Criteron has no reference to physical conditions
			F	In	Hiring assistant to record
18.3: The facility shall keep complete and accurate records concerning any antibiotic or other drug use at both the hatchery and the farm.	H	Recording	H	In	
			S	Out	Criteron has no reference to social conditions
			N	Out	Criteron has no reference to natural conditions
			P	Out	Criteron has no reference to physical conditions
			F	In	Hiring assistant to record
18.4: Complete and accurate records shall be maintained on the use of sulfites or other approved food-processing aids/additives in shrimp, as well as the use of herbicides, algicides and other pesticides.	H	Recording	H	In	
			S	Out	Criteron has no reference to social conditions
			N	Out	Criteron has no reference to natural conditions
			P	Out	Criteron has no reference to physical conditions
			F	In	Hiring assistant to record
18.5: Complete and accurate records regarding manufacturer and lot numbers for each feed used shall be maintained.	H	Recording	H	In	
			S	In	Manufacturer can assist in providing information, provide proper records
			N	Out	Criteron has no reference to natural conditions
			P	Out	Criteron has no reference to physical conditions

			F	In	Hiring assistant to record
18.6: The facility shall maintain complete and accurate records of the sources and numbers of postlarvae or fingerlings stocked, stocking dates and all feeds used for each culture unit.	H	Recording	H	In	
			S	In	Manufacturer can assist in providing information, provide proper records
			N	Out	Criteron has no reference to natural conditions
			P	Out	Criteron has no reference to physical conditions
			F	In	Hiring assistant to record
18.7: Complete and accurate records regarding the harvest date, harvest quantity, movement document number (if applicable) and processing plant(s) or purchaser(s) shall be maintained. If product lots are destined to more than one plant or purchaser, each lot shall be separately identified.	H	Documentation	H	In	
			S	In	Social network, authorities, brokers can help with providing movement documents
			N	Out	Criteron has no reference to natural conditions
			P	Out	Criteron has no reference to physical conditions
			F	In	Hiring assistant to record
18.8: In order to use the BAP logo, facilities shall have such use approved and registered in advance with BAP Management.	H	Managment	H	In	
			S	In	Social networks or collective group facilitate help in contacting or coordinating with BAP representative
			N	Out	Criteron has no reference to natural conditions
			P	Out	Criteron has no reference to physical conditions
			F	Out	Criteron has no reference to financial conditions
18.9: The facility shall keep records of any customer complaints related to its products' compliance with the BAP standards.	H	Documentation	H	In	
			S	Out	Criteron has no reference to social conditions
			N	Out	Criteron has no reference to natural conditions
			P	Out	Criteron has no reference to physical conditions

			F	In	Hiring assistant to record
18.10: The facility shall keep records of investigations of such complaints and actions taken to address/correct them.	H	Documentations	H	In	
			S	Out	Criteron has no reference to social conditions
			N	Out	Criteron has no reference to natural conditions
			P	Out	Criteron has no reference to physical conditions
			F	In	Hiring assistant to record
**19. Shrimp-Specific Standards - Environment**					
19.1: The mean water exchange rate shall not exceed 10% per day (i.e., on an annual basis, 36 x total pond volume). This limit does not apply to shrimp ponds in deserts.	H	Controlling the exchange rate	H	In	
			S	Out	Criteron has no reference to social conditions
			N	In	Availability of water for proper water exchange rate
			P	In	Equipment
			F	In	Ability to buy and install proper water exchange system
19.2: If used, sulfites shall be applied in a manner that will yield a tissue concentration within regulatory limits.	H	Managment	H	In	Knowledge on regulatory limits
			S	Out	Criteron has no reference to social conditions
			N	Out	Criteron has no reference to natural conditions
			P	In	Proper sulfites
			F	Out	Criteron has no reference to financial conditions
19.3: Sulfite solutions shall be deactivated or neutralized, for example by 48-hour retention, prior to release into natural water bodies.	H	Knowledge on sulfite solutions	H	In	
			S	Out	Criteron has no reference to social conditions
			N	Out	Criteron has no reference to natural conditions
			P	In	Equipment
			F	Out	Criteron has no reference to financial conditions
