# Supplementary material for: A capability approach to assess aquaculture sustainability standard compliance
Source: PLoS One. 2020 Jan 23;15(1):e0227812. doi: 10.1371/journal.pone.0227812 (PMC6977730; doi:10.1371/journal.pone.0227812)
Supplement: S2 Table — (PDF) [file pone.0227812.s002.pdf]

S2 Table. Assessment of prescribed capitals and bundles of capitals for the Aquaculture Stewardship Council.

**ASC**		**Prescribed capital**	**Justification**	**Bundle of capitals**	**In/out**	**Justification (Bundle of capitals)**
**PRINCIPLE 1. COMPLY WITH ALL APPLICABLE NATIONAL AND LOCAL LAWS AND REGULATIONS** Criterion 1.1: Documented compliance with local and national legal requirements						
1.1.1	**Indicator:** Compliance with local and national laws or regulation.	H	Managment	H	In	
				S	Out	Criteron has no reference to social conditions
	**Requirement:** <u>Proofs of permits</u> or other relevant documentation available for applicable regulations.			N	Out	Criteron has no reference to natural conditions
				P	Out	Criteron has no reference to physical conditions
				F	Out	Criteron has no reference to financial conditions
1.1.2	**Indicator:** Transparency on legal compliance.	H	Documentation	H	In	
	**Requirement:** Government-issued operational permits and licenses are publicly available one month after request.			S	Out	Criteron has no reference to social conditions
				N	In	Ownership right of lands or land lease
				P	Out	Criteron has no reference to physical conditions
				F	Out	Criteron has no reference to financial conditions
**PRINCIPLE 2. SITE FARMS IN ENVIRONMENTALLY SUITABLE LOCATIONS WHILE CONSERVING BIODIVERSITY AND IMPORTANT NATURAL ECOSYSTEMS** Criterion 2.1: Biodiversity Environmental Impact Assessment (B-EIA)						
2.1.1	**Indicator:** Farm owners shall <u>commission a participatory B-EIA</u> and disseminate results and outcomes openly in <u>locally appropriate language</u>. The B-EIA process and document must follow the outline in Appendix A.	H	Management	H	In	
				S	Out	Criteron has no reference to social conditions
	**Requirement:** Report available and complies to B-EIA Appendix I	F	Paid experts for assessment	N	Out	Criteron has no reference to natural conditions
				P	Out	Criteron has no reference to physical conditions

				F	In	
Criterion 2.2: Conservation of protected areas or critical habitats						
2.2.1	**Indicator:** Allowance for siting in Protected Areas (PAs).<br>**Requirement:** None, except within PAs with IUCN category V if the farming system is regarded as traditional land use, or category VI if the farm was built legally prior to the designation of the PA and in both cases is in compliance with the management objectives and plan of the PA, and shrimp farming is no more than 25% of the total PA area.	N	Farm location	H	Out	Criteron has no reference to human conditions
				S	Out	Criteron has no reference to social conditions
				N	In	
				P	Out	Criteron has no reference to physical conditions
				F	In	Financial capital for the ability to relocate the farm
2.2.2	**Indicator:** Allowance for siting in mangrove ecosystems and other natural wetlands, or areas of ecological importance as determined by the B-EIA or national/state/local authority plans/list.<br>**Requirement:** None for farms built (with or without permits) after May 1999, except for pumping stations and inlet/outlet canals provided they have been permitted by authorities and an equivalent area is rehabilitated [18] as compensation. For farms built or permitted before May 1999, farmers are required to compensate/offset impacts via rehabilitation as determined by the B-EIA, or the national/state/local authority plans/list, or 50% of the affected ecosystem (whichever is greater).	N	Farm location	H	Out	Criteron has no reference to human conditions
				S	Out	Criteron has no reference to social conditions
				N	In	
				P	Out	Criteron has no reference to physical conditions
				F	In	Financial capital for the ability to relocate the farm
Criterion 2.3: Consideration of habitats critical for endangered species						
2.3.1	**Indicator:** Allowance for siting farms in critical habitats of endangered species as defined by the IUCN Red List, national listing processes or other official lists.<br>**Requirement:** None	N	Farm location	H	Out	Criteron has no reference to human conditions
				S	Out	Criteron has no reference to social conditions
				N	In	
				P	Out	Criteron has no reference to physical conditions
				F	In	Financial capital for the ability to relocate the farm
2.3.2	**Indicator:** Maintain habitats critical for endangered species within farm boundaries and implement protection measures of such areas.	N		H	Out	Criteron has no reference to human conditions
				S	Out	Criteron has no reference to social conditions
				N	In	

	**Requirement:** Implement protection measures of habitats identified by the B-EIA process.			P	Out	Criteron has no reference to physical conditions
				F	Out	Criteron has no reference to financial conditions
Criterion 2.4: Ecological buffers, barriers and corridors						
2.4.1	**Indicator:** Coastal barriers: Minimum permanent barrier (or natural) between farm and marine environments.	N	Natural barriers	H	Out	Criteron has no reference to human conditions
				S	Out	Criteron has no reference to social conditions
	**Requirement:** As defined in legislation at the time of construction, or as determined by the B-EIA, or following the indications in the Guidance below, whichever is greater.	P	Barriers	N	In	
				P	In	
				F	In	Investiment in building barrier
2.4.2	**Indicator:** Riparian buffers: Minimum width of permanent native and natural vegetation between farms and natural aquatic/brackish environments .	N	Natural barriers	H	Out	Criteron has no reference to social conditions
				S	Out	Criteron has no reference to social conditions
	**Requirement:** As defined in national legislation at the time of construction, or as determined is necessary by the B-EIA, or following the indications given in the Guidance below, whichever is greater.			N	In	
				P	Out	Criteron has no reference to physical conditions
				F	Out	Criteron has no reference to financial conditions
2.4.3	**Indicator:** Corridors: Minimum width of permanent native and natural vegetation through farms to provide human or native wildlife movement across agricultural landscapes.	N	Natural barriers	H	Out	Criteron has no reference to social conditions
				S	Out	Criteron has no reference to social conditions
	**Requirement:** As defined in national legislation at the time of construction, or as determined necessary for wildlife by the B-EIA, or access issues identified during B-EIA/p-SIA. Needs for wildlife movement identified during B-EIA.			N	In	
				P	Out	Criteron has no reference to physical conditions
				F	Out	Criteron has no reference to financial conditions
Criterion 2.5: Prevention of salinization of freshwater and soil resources						
2.5.1	**Indicator:** Allowance for discharging saline water to natural freshwater bodies.	H	Water management	H	In	
				S	Out	Criteron has no reference to social conditions
	**Requirement:** None			N	Out	Criteron has no reference to natural conditions
				P	In	Infrastructure

				F	Out	Criteron has no reference to financial conditions
2.5.2	**Indicator:** Allowance for the use of fresh groundwater in ponds<br><br>**Requirement:** None	H	Water management	H	In	
				S	Out	Criteron has no reference to social conditions
				N	In	Availability of water from other sources
				P	Out	Criteron has no reference to physical conditions
				F	In	Ability to buy water from other sources
2.5.3	**Indicator:** Water-specific conductance or chloride concentration in freshwater wells used by the farm or located on adjacent properties.<br><br>**Requirement:** For all freshwater wells (identified prior to full assessment), specific conductance may not exceed 1,500 µS/cm and/or chloride concentration may not exceed 300 mg/L.	N		H	In	Knowledge on concentration values, calculation
				S	Out	Criteron has no reference to social conditions
				N	In	
				P	Out	Criteron has no reference to physical conditions
				F	Out	Criteron has no reference to financial conditions
2.5.4	**Indicator:** Soil-specific conductance or chloride concentration in adjacent land ecosystems and agricultural fields.<br><br>**Requirement:** No net increase when compared to the first year of monitoring	N		H	In	Management
				S	Out	Criteron has no reference to social conditions
				N	In	
				P	Out	Criteron has no reference to physical conditions
				F	Out	Criteron has no reference to financial conditions
2.5.5	**Indicator:** Specific conductance or chloride concentration of sediment prior to disposal outside the farm.<br><br>**Requirement:** The specific conductance or chloride concentration values must not exceed those of the soil in the disposal area.	N		H	In	Management
				S	Out	Criteron has no reference to social conditions
				N	In	
				P	Out	Criteron has no reference to physical conditions
				F	Out	Criteron has no reference to financial conditions
**PRINCIPLE 3:DEVELOP AND OPERATE FARMS WITH CONSIDERATION FOR SURROUNDING COMMUNITIES**						

Criterion 3.1: All impacts on surrounding communities, ecosystem users and land owners are accounted for and are, or will be, negotiated in an open and accountable manner						
3.1.1	**Indicator:** Farm owners shall commission or undertake a participatory Social Impact Assessment (p-SIA) and disseminate results and outcome openly in locally appropriate language. Local government and at least one civil society organization chosen by the community shall have a copy of this document. The p-SIA process and document includes a participatory (shared) impact and risk analysis with surrounding communities and stakeholders. The participatory element (community input and response) is visibly included in the report. Outcomes as agreed between farm and surrounding community on how to manage risks and impacts are included in the report.<br>**Requirement:** The p-SIA report adheres to the steps outlined in Appendix II; is available in the local government, the community and through the chosen community civil organization; and the report lists dates of meetings and names of participants.	S		H	In	Management
				S	In	
				N	Out	Criteron has no reference to natural conditions
		F	Ability to hire experts to conduct assessment	P	Out	Criteron has no reference to physical conditions
				F	In	Ability to hire experts to conduct assessment
Criterion 3.2: Complaints by affected stakeholders are being resolved						
3.2.1	**Indicator:** Farm owners shall develop and apply a verifiable conflict resolution policy for local communities. The policy shall state how conflicts identified in the p-SIA and new complaints will be tracked transparently, how third party mediation can be part of the process and explain how to respond to all received complaints. Complaint boxes, complaint registers and complaint acknowledgement receipts (in local language(s)) are used.<br>**Requirement:** Completed	S		H	Out	Criteron has no reference to human conditions
				S	In	
				N	Out	Criteron has no reference to natural conditions
				P	Out	Criteron has no reference to physical conditions
				F	Out	Criteron has no reference to financial conditions
3.2.2	**Indicator:** Areas of conflict or dispute are recorded and shared among farm, local government and surrounding community representatives. At least 50% of the conflicts shall be resolved within one year from the date of being filed, and a total of 75% in the period between two successive audits.	S		H	In	Management
				S	In	
				N	Out	Criteron has no reference to natural conditions
				P	Out	Criteron has no reference to physical conditions

	**Requirement:** Completed			F	Out	Criteron has no reference to financial conditions
Criterion 3.3: Transparency in providing employment opportunities within local communities						
3.3.1	**Indicator:** Farms shall document evidence of advertising positions to people living within daily traveling distance from the farm before hiring people who cannot travel to and from home on a daily basis.<br><br>**Requirement:** Proof of dated job opening advertisements in surrounding villages, by means of either/or signposts, billboards or ads in local magazines or newspapers.	H	Documentation	H	In	
				S	Out	Criteron has no reference to social conditions
				N	Out	Criteron has no reference to natural conditions
				P	Out	Criteron has no reference to physical conditions
				F	Out	Criteron has no reference to financial conditions
3.3.2	**Indicator:** Justifications for employment of each worker are available, and based on profile and merits (skills, experience or CV in the case of hired migrant worker).<br>**Requirement:** Written and dated records of applications and interviews with applicants, including stating whether they are from an outside community or from the local area. Records must also state reasons for successful or unsuccessful applications. Name and contact	H	Management	H	In	
				S	Out	Criteron has no reference to social conditions
				N	Out	Criteron has no reference to natural conditions
				P	Out	Criteron has no reference to physical conditions
				F	Out	Criteron has no reference to financial conditions
Criterion 3.4: Contract farming arrangements (if practiced) are fair and transparent to the contract farmer						
3.4.1	**Indicator:** Written contract agreements<br><br>**Requirement:** The contracts are written in an appropriate language, and co-signed copies are kept by both parties.	H	Management	H	In	
				S	Out	Criteron has no reference to social conditions
				N	Out	Criteron has no reference to natural conditions
				P	Out	Criteron has no reference to physical conditions
				F	Out	Criteron has no reference to financial conditions
3.4.2	**Indicator:** Contract provisions<br><br>**Requirement:** The contracts comply with the Appendix III (part A) on content of basic provisions to ensure that conditions of the agreement are mutually understood.	H	Management	H	In	
				S	Out	Criteron has no reference to social conditions
				N	Out	Criteron has no reference to natural conditions
				P	Out	Criteron has no reference to physical conditions

				F	Out	Criteron has no reference to financial conditions
3.4.3	**Indicator:** Transparency and openness of negotiations<br>**Requirement:** Meetings between the purchaser and the contract farmers to discuss and negotiate agreements are held at least twice a year and documented. Meetings are attended by at least three representatives of the farm group or cooperative. All members contributing to the supply contract must sign their agreement to the negotiated terms.	S		H	Out	Criteron has no reference to human conditions
				S	In	
				N	Out	Criteron has no reference to natural conditions
				P	Out	Criteron has no reference to physical conditions
				F	Out	Criteron has no reference to financial conditions
**PRINCIPLE 4: OPERATE FARMS WITH RESPONSIBLE LABOR PRACTICES**<br>Criterion 4.1: Child labor and young workers						
4.1.1	**Indicator:** Minimum age of hired workers<br><br>**Requirement:** 18 years of age	H	Management	H	In	
				S	Out	Criteron has no reference to social conditions
				N	Out	Criteron has no reference to natural conditions
				P	Out	Criteron has no reference to physical conditions
				F	Out	Criteron has no reference to financial conditions
Criterion 4.2: Forced, bonded compulsory labor						
4.2.1	**Indicator:** Right to full final payment and benefits<br><br>**Requirement:** Employers will not withhold any part of employee salary, property or benefits upon the termination of employment.	H	Management	H	In	
				S	Out	Criteron has no reference to social conditions
				N	Out	Criteron has no reference to natural conditions
				P	Out	Criteron has no reference to physical conditions
				F	In	Financial capital is involved with salary
4.2.2	**Indicator:** Employees have the right to keep identity documents and work permits<br><br>**Requirement:** Hired workers are not required to surrender original identity documents with their employer upon commencing employment.	H	Management	H	In	
				S	Out	Criteron has no reference to social conditions
				N	Out	Criteron has no reference to natural conditions
				P	Out	Criteron has no reference to physical conditions
				F	Out	Criteron has no reference to financial conditions

4.2.3	**Indicator:** Hired workers have the freedom of movement outside working hours<br><br>**Requirement:** Hired workers shall be free to leave the workplace and manage their resting time.	H	Management	H	In	
				S	Out	Criteron has no reference to social conditions
				N	Out	Criteron has no reference to natural conditions
				P	Out	Criteron has no reference to physical conditions
				F	Out	Criteron has no reference to financial conditions
Criterion 4.3: Discrimination in the work environment						
4.3.1	**Indicator:** Anti-discrimination policy in place, including, but not limited to, how to deal with discrimination in the workplace and equal access to all jobs in relation to gender, age, origin (locals vs. migrants), race or religion, and outlining clear and transparent company procedures are to raise/file and respond to discrimination complaints. Clear and transparent company procedures are outlined to raise/file and respond to discrimination complaints.<br><br>**Requirement:** Policy document is available on farm and its content is known by workers. Evidence that the procedures are in place and being used. No complaints from workers as to adherence to it.	H	Management	H	In	
				S	In	Social rights
				N	Out	Criteron has no reference to natural conditions
				P	Out	Criteron has no reference to physical conditions
				F	Out	Criteron has no reference to financial conditions
4.3.2	**Indicator:** Number of incidences of discrimination<br><br>**Requirement:** None	H	Documentation	H	In	
				S	Out	Criteron has no reference to social conditions
				N	Out	Criteron has no reference to natural conditions
				P	Out	Criteron has no reference to physical conditions
				F	Out	Criteron has no reference to financial conditions
4.3.3	**Indicator:** Equality of salaries and opportunities. All hired workers, independent of their gender, origin, race or religion, receive equal pay, benefits, promotion opportunities, job security arrangements and training opportunities for equal work at equal role and experience levels within the same hierarchical position.<br><br>**Requirement:** Evidence of equality of salaries and opportunities.	H	Management	H	In	
				S	In	Social rights, norms
				N	Out	Criteron has no reference to natural conditions
				P	Out	Criteron has no reference to physical conditions
				F	Out	Criteron has no reference to financial conditions
4.3.4	**Indicator:** Respect of maternity rights and benefits	H	Management	H	In	

	**Requirement:** Employers shall not test for pregnancy and shall not sanction and/or dismiss on the basis of marital status and shall guarantee legal rights to pregnancy/maternity leave.			S	Out	Criteron has no reference to social conditions
				N	Out	Criteron has no reference to natural conditions
				P	Out	Criteron has no reference to physical conditions
				F	Out	Criteron has no reference to financial conditions
Criterion 4.4: Work environment health and safety						
4.4.1	**Indicator:** Percentage of workers trained in health and safety practices, procedures and policies relevant to the job. Safety equipment provided and maintained and in use.<br>**Requirement:** 100% of workers trained. Certificates of training issued by the relevant competent national or provincial authority or by such an authority-recognized training center are required for operations with more than five employees and evidence that safety equipment is in use by workers.	H	Training	H	In	
				S	Out	Criteron has no reference to social conditions
		P	Equipment	N	Out	Criteron has no reference to natural conditions
				P	In	
				F	In	Ability to buy and maintainence safety equipment
4.4.2	**Indicator:** Monitoring of accidents and incidents and corrective actions.<br><br>**Requirement:** All job-related accidents and incidents must be recorded and corrective actions must be documented and implemented.	H	Documentation	H	In	
				S	Out	Criteron has no reference to social conditions
				N	Out	Criteron has no reference to natural conditions
				P	Out	Criteron has no reference to physical conditions
				F	Out	Criteron has no reference to financial conditions
4.4.3	**Indicator:** Medical expenses coverage.<br><br>**Requirement:** Employer must provide a proof of coverage of all expenses related to any accident/injury occurring under the responsibility of the employer when not covered under national law.	F		H	Out	Criteron has no reference to human conditions
				S	Out	Criteron has no reference to social conditions
				N	Out	Criteron has no reference to natural conditions
				P	Out	Criteron has no reference to physical conditions
				F	In	
Criterion 4.5: Minimum and fair wages or "decent wages"						
4.5.1	**Indicator:** Minimum wage level as applicable to their specific job/task description.<br>**Requirement:** All hiredworkers, including temporary workers, must receive pay greater than or equal to legally set minimum wage	H	Management	H	In	
				S	Out	Criteron has no reference to social conditions
				N	Out	Criteron has no reference to natural conditions

	according to country or region in country (whichever applies). Payments must be done: in legal tender, at the workplaceor in the worker's bank account, at the frequency specified in the contract, with clearly documented pay slips given to workers, including identification of any deductions.			P<br>F	Out<br>In	Criteron has no reference to physical conditions<br>Financial capital is involved in salaries
4.5.2	**Indicator:** <u>Permanent workers are paid fair wages.</u> Salaries, if not already at a "fair wage" level, are gradually increased to include sufficient funds for a worker's basic needs plus a discretionary income that allows for savings and/or pension payments<br>**Requirement:** Evidence available confirming fair wages or gradual pay rises through time-series of pay slips in farm administration and in the hands of workers	H	Management	H<br>S<br>N<br>P<br>F	In<br>Out<br>Out<br>Out<br>In	 Criteron has no reference to social conditions<br>Criteron has no reference to natural conditions<br>Criteron has no reference to physical conditions<br>Financial capital is involved in salaries
4.5.3	**Indicator:** Punishment through infringement of workers' rights or wages.<br><br>**Requirement:** <u>No allowance for withholding any part or all of worker salaries, benefits or rights acquired or stipulated or rights acquired or stipulated by law</u>. Not even as punishment of (alleged) wrongdoings on the part of the worker (cf. ILO 29 and 105).	H	Management	H<br>S<br>N<br>P<br>F	In<br>Out<br>Out<br>Out<br>Out	 Criteron has no reference to social conditions<br>Criteron has no reference to natural conditions<br>Criteron has no reference to physical conditions<br>Criteron has no reference to financial conditions
4.5.4	**Indicator:** There is a <u>mechanism for setting wages and benefits</u> (including, if applicable, the combination of pay and harvest sharing arrangements).<br><br>**Requirement:** Decision-making criteria and processes for wage and benefit adjustments are known by all workers	H	Management	H<br>S<br>N<br>P<br>F	In<br>Out<br>Out<br>Out<br>Out	 Criteron has no reference to social conditions<br>Criteron has no reference to natural conditions<br>Criteron has no reference to physical conditions<br>Criteron has no reference to financial conditions
4.5.5	**Indicator:** Revolving labor-contract schemes designed to deny long-time workers full access to fair and equitable remuneration and other benefits<br>**Requirement:** Prohibited	H	Management	H<br>S<br>N<br>P	In<br>Out<br>Out<br>Out	 Criteron has no reference to social conditions<br>Criteron has no reference to natural conditions<br>Criteron has no reference to physical conditions

				F	Out	Criteron has no reference to financial conditions
Criterion 4.6: Access to freedom of association and the right to collective bargaining						
4.6.1	**Indicator:** Percentage of workers with access to trade unions, worker organizations, and/or have the ability to self-organize and the ability to bargain collectively or to have access to representative(s) chosen by workers without management interference<br>**Requirement:** 100% of workers have access, if they so choose, to worker organizations capable of representing them independently from the employer.	S		H	Out	Criteron has no reference to human conditions
				S	In	
				N	Out	Criteron has no reference to natural conditions
				P	Out	Criteron has no reference to physical conditions
				F	Out	Criteron has no reference to financial conditions
4.6.2	**Indicator:** Members of unions or worker organizations are not discriminated against by employers<br><br>**Requirement:** Employers shall not interfere with or penalize workers for exercising their right of representation.	H	Management	H	In	
				S	Out	Criteron has no reference to social conditions
				N	Out	Criteron has no reference to natural conditions
				P	Out	Criteron has no reference to physical conditions
				F	Out	Criteron has no reference to financial conditions
Criterion 4.7: Harassment and disciplinary practices in the working environment causing temporary or permanent physical and/or mental harm						
4.7.1	**Indicator:** Fairness of disciplinary measures<br><br>**Requirement:** No instances of abuses	H	Management	H	In	
				S	Out	Criteron has no reference to social conditions
				N	Out	Criteron has no reference to natural conditions
				P	Out	Criteron has no reference to physical conditions
				F	Out	Criteron has no reference to financial conditions
4.7.2	**Indicator:** Clear, fair and transparent disciplinary policies and procedures<br><br>**Requirement:** Evidence of documentation and communication to all workers.	H	Management	H	In	
				S	Out	Criteron has no reference to social conditions
				N	Out	Criteron has no reference to natural conditions

				P	Out	Criteron has no reference to physical conditions
				F	Out	Criteron has no reference to financial conditions
4.7.3	**Indicator:** Prohibition of harassment.<br><br>**Requirement:** Evidences that any instances have been addressed and resolved.	H	Management	H	In	
				S	Out	Criteron has no reference to social conditions
				N	Out	Criteron has no reference to natural conditions
				P	Out	Criteron has no reference to physical conditions
				F	Out	Criteron has no reference to financial conditions
Criterion 4.8: Overtime compensation and working hours						
4.8.1	**Indicator:** Maximum number of regular working hours: Eight hours/day or 48 hours/week (maximum average over 17 week period) including "stand-by" hours; with at least one full day (including two nights) off in every seven-day period.<br>**Requirement:** Reflected in records available on the farm and 100% compliance expressed in worker interviews.	H	Management	H	In	
				S	Out	Criteron has no reference to social conditions
				N	Out	Criteron has no reference to natural conditions
				P	Out	Criteron has no reference to physical conditions
				F	Out	Criteron has no reference to financial conditions
4.8.2	**Indicator:** Right to leave the farm after completion of daily work duties<br><br>**Requirement:** Evidence of freedom of movement for all employees.	H	Management	H	In	
				S	Out	Criteron has no reference to social conditions
				N	Out	Criteron has no reference to natural conditions
				P	Out	Criteron has no reference to physical conditions
				F	Out	Criteron has no reference to financial conditions
4.8.3	**Indicator:** Minimum time off from work, with the right but not the obligation to leave farm premises if accommodations are on the farm, except where both the employer and employee agree that off-days cannot be accommodated on the farm	H	Management	H	In	
				S	Out	Criteron has no reference to social conditions
				N	Out	Criteron has no reference to natural conditions

	**Requirement:** Four full 24-hour periods per month			P	Out	Criteron has no reference to physical conditions
				F	Out	Criteron has no reference to financial conditions
4.8.4	**Indicator:** <u>Transport provided to workers</u> (in cases where farm locations are remote) to allow workers to enjoy relaxation at home, with family or in places of recreation of their choosing.	H	Management	H	In	
				S	Out	Criteron has no reference to social conditions
				N	Out	Criteron has no reference to natural conditions
	**Requirement:** The <u>farm owner shall provide transport</u> to and from the first location from which regular public transport is available.			P	In	Workers transportation to farm
				F	Out	Ability to hire and provide transportation
4.8.5	**Indicator:** <u>Overtime compensation is provided</u>	H	Management	H	In	
				S	Out	Criteron has no reference to social conditions
	**Requirement:** Paid at a premium rat of at least 25% above the wage for normal hours			N	Out	Criteron has no reference to natural conditions
				P	Out	Criteron has no reference to physical conditions
				F	In	Financial capital is involved in salaries
4.8.6	**Indicator:** Overtime is voluntary, and not longer than 12 hours/week.	H	Management	H	In	
				S	Out	Criteron has no reference to social conditions
				N	Out	Criteron has no reference to natural conditions
	**Requirement:** Occasionally (not on a regular basis).			P	Out	Criteron has no reference to physical conditions
				F	Out	Criteron has no reference to financial conditions
4.8.7	**Indicator:** Rights to maternity leave, including daily breaks or a reduction of hours of work to address child care needs.	H	Management	H	In	
				S	Out	Criteron has no reference to social conditions
	**Requirement:** Maternity leave is a minimum of 14 weeks (total period off-duty period including before and/or after moment of birth) and includes a guarantee to return to the job. Payment during this period shall minimally be at the level of social insurance offered by the country.			N	Out	Criteron has no reference to natural conditions
				P	Out	Criteron has no reference to physical conditions
				F	Out	Criteron has no reference to financial conditions
Criterion 4.9: Worker contracts are fair and transparent						
4.9.1		H	Management	H	In	

	**Indicator:** Allowance for labor-only contracting relationships or false apprenticeship schemes including revolving / consecutive labor contracts to deny benefit accrual.<br>**Requirement:** None			S	Out	Criteron has no reference to social conditions
				N	Out	Criteron has no reference to natural conditions
				P	Out	Criteron has no reference to physical conditions
				F	Out	Criteron has no reference to financial conditions
4.9.2	**Indicator:** All workers have the appropriate and applicable permits for working in the country.<br>**Requirement:** Employer has a list of permit reference numbers or copies of permits for all concerned workers.	H	Management	H	In	
				S	Out	Criteron has no reference to social conditions
				N	Out	Criteron has no reference to natural conditions
				P	Out	Criteron has no reference to physical conditions
				F	Out	Criteron has no reference to financial conditions
4.9.3	**Indicator:** Workers are fully aware of their employment conditions and confirmed their agreement (verbal or written). Written employment policies and procedures are required when there are more than five hired workers.<br>**Requirement:** Evidence of contract agreement for all workers. Written contracts: a complete contract is filed in the office, mutually signed and copies are available to the worker. Verbal agreements: employer and worker cite consistent employment conditions in independent interviews.	H	Management	H	In	
				S	Out	Criteron has no reference to social conditions
				N	Out	Criteron has no reference to natural conditions
				P	Out	Criteron has no reference to physical conditions
				F	Out	Criteron has no reference to financial conditions
4.9.4	**Indicator:** Probation period stipulated in contract<br>**Requirement:** The probation period shall follow prevalent law in the country, but not be more than 30 days in cases laws do not exist or are not applicable.	H	Management	H	In	
				S	Out	Criteron has no reference to social conditions
				N	Out	Criteron has no reference to natural conditions
				P	Out	Criteron has no reference to physical conditions
				F	Out	Criteron has no reference to financial conditions

4.9.5	**Indicator:** In subcontracting or home-working arrangements, the farm owner shall assure that labor laws, social security laws and ratified ILO provisions have been duly respected and complied with.<br>**Requirement:** Confirmation that sub-contractors and intermediaries have contracts with their workers that are in accordance with laws and regulations.	H	Management	H	In	
				S	Out	Criteron has no reference to social conditions
				N	Out	Criteron has no reference to natural conditions
				P	Out	Criteron has no reference to physical conditions
				F	Out	Criteron has no reference to financial conditions
Criterion 4.10: Fair and transparent worker-management systems						
4.10.1	**Indicator:** The employer ensures that all workers have access to appropriate channels of communication with managers on matters relating to labor rights and working conditions.<br>**Requirement:** Management and the full workforce meet at least twice per year on the basis of written agendas and written minutes of the meetings are available.	S		H	In	Management
				S	In	
				N	Out	Criteron has no reference to natural conditions
				P	Out	Criteron has no reference to physical conditions
				F	Out	Criteron has no reference to financial conditions
4.10.2	**Indicator:** Percentage of issues raised by workers which are recorded, responded to and monitored by employer.<br>**Requirement:** 100%	H	Management	H	In	
				S	Out	Criteron has no reference to social conditions
				N	Out	Criteron has no reference to natural conditions
				P	Out	Criteron has no reference to physical conditions
				F	Out	Criteron has no reference to financial conditions
4.10.3	**Indicator:** Clear plan, with process actions and timeframe, is developed to address complaints, and comply with.<br>**Requirement:** List of complaints, corresponding action plan and timeframe for resolution is available.	H	Management	H	In	
				S	Out	Criteron has no reference to social conditions
				N	Out	Criteron has no reference to natural conditions
				P	Out	Criteron has no reference to physical conditions
				F	Out	Criteron has no reference to financial conditions
4.10.4	**Indicator:** Percentage of complaints that are resolved within three months after being received.	H	Management	H	In	
				S	Out	Criteron has no reference to social conditions
				N	Out	Criteron has no reference to natural conditions

	**Requirement:** 90%, according to the timeframe of 4.10.3.			P	Out	Criteron has no reference to physical conditions
				F	Out	Criteron has no reference to financial conditions
Criterion 4.11: Living conditions for workers accommodated on the farm						
4.11.1	**Indicator:** <u>Living conditions</u> for workers accomodated on the farm are decent and safe.<br>**Requirement:** All facilities are clean, sanitary, rainproof, safe and suitable for habitation. Shared quarters need to include provisions that allow for visibility privacy, such as walls, curtains provisions that allow for visibility privacy, such as walls, curtains or movable rattan/bamboo screens. Potable water and cooking facilities or catering facilities are available to all accomodated workers on the farm premises.	P	Infrastructure	H	Out	Criteron has no reference to human conditions
				S	Out	Criteron has no reference to social conditions
				N	Out	Criteron has no reference to natural conditions
				P	In	
				F	In	Ability to buid and provide proper housing
4.11.2	**Indicator:** Adequate facilities for women.<br><br>**Requirement:** Separate and suitable sanitary and toilet facilities are available for men and women, with the possible exception of married couples being accommodated together.	P	Infrastructure	H	Out	Criteron has no reference to human conditions
				S	Out	Criteron has no reference to social conditions
				N	Out	Criteron has no reference to natural conditions
				P	In	
				F	In	Ability to buid and provide proper housing
**PRINCIPLE 5: MANAGE SHRIMP HEALTH AND WELFARE IN A RESPONSIBLE MANNER**<br>Criterion 5.1: Disease prevention						
5.1.1	**Indicator:** <u>Develop and maintain an operational health plan</u> addressing:1) Pathogens that can come from the surrounding environment into the farm (e.g., predator and vector control), 2) Pathogens that can spread from the farm to the surrounding environment (e.g., effluent filtration/sterilization, and waste such as dead-shrimp management) 3) Spreading of pathogens within the farm. Critical to avoid cross contamination, detect and prevent emerging pathogen(s), and monitor external signs of pathologies and moribund animals<br>**Requirement:** Demonstration that the operational health plan is functional	H	Management	H	In	
				S	Out	Criteron has no reference to social conditions
				N	Out	Criteron has no reference to natural conditions
				P	Out	Criteron refers to physical conditions but focus in on operational health plan
				F	In	Hiring assistant in developing the plan

5.1.2	**Indicator:** Filtration of inlet water for minimizing the entry of pathogens<br><br>**Requirement:** Nets, grills, screens or barriers of the appropriate mesh size are present on all farm or pond inlets.	P	Equipment	H	Out	Criteron has no reference to human conditions
				S	Out	Criteron has no reference to social conditions
				N	Out	Criteron has no reference to natural conditions
				P	In	
				F	In	Ability to buy and install proper barrier
5.1.3	**Indicator:** Annual <u>average farm survival rate</u> (SR):1) Unfed and non-permanently aerated pond systems2) Fed but non-permanently aerated pond systems3) Fed and permanently aerated pond systems.<br><br>**Requirement:** SR >25%SR >45%SR >60%	N		H	Out	Criteron has no reference to human conditions
				S	Out	Criteron has no reference to social conditions
				N	In	
				P	Out	Criteron has no reference to physical conditions
				F	Out	Criteron has no reference to financial conditions
5.1.4	**Indicator:** <u>Percent of stocked postlarvae</u> (PLs) that are Specific Pathogen Free (SPF) or Specific Pathogen Resistant (SPR) for all important pathogens.<br>**Requirement:** 100% if commercially available , i.e., if for any given species, at least 20% of the PLs stocked in the country are from SPF or SPR stocks, then the supply is deemed commercially available. If not commercially available, PLs screened for all important pathogens can be used.	N		H	Out	Criteron has no reference to human conditions
				S	Out	Criteron has no reference to social conditions
		F	Commercially available, then purchase	N	In	
				P	Out	Criteron has no reference to physical conditions
				F	In	
Criterion 5.2: Predator control						
5.2.1	**Indicator:** <u>Allowance for intentional lethal predator control</u> of any protected, threatened or endangered species <u>as defined by the International Union for Conservation of Nature (IUCN) Red List national listing processes, or other official lists</u>.<br>**Requirement:** None	H	Management of not to use it	H	In	
				S	Out	Criteron has no reference to social conditions
				N	Out	Criteron has no reference to natural conditions
				P	In	Availability of proper chemicals
				F	In	Ability to buy proper equipment
5.2.2	Indicator: Allowance for <u>use of lead shot and select chemicals for predator control</u>.	H	Management of not to use it	H	In	
				S	Out	Criteron has no reference to social conditions

				N	Out	Criteron has no reference to natural conditions
	**Requirement:** None			P	In	Availability of proper chemicals
				F	In	Ability to buy proper chemicals, devices
5.2.3	**Indicator:** In case lethal predator control is used, a basic monitoring program must be in place for documenting the frequency of visits, variety of species and number of animals interacting with the farm.	H	Monitoring	H	In	
				S	Out	Criteron has no reference to social conditions
				N	Out	Criteron has no reference to natural conditions
	**Requirement:** Yes			P	Out	Criteron has no reference to physical conditions
				F	Out	Criteron has no reference to financial conditions
Criterion 5.3: Disease management and treatment						
5.3.1	**Indicator:** Allowance for use of antibiotics and medicated feed on ASC-labeled products (farm can be certified but specific product receiving medicated feed will not be authorized to carry ASC label).	H	Knowledge on what not to use	H	In	
				S	Out	Criteron has no reference to social conditions
				N	Out	Criteron has no reference to natural conditions
	**Requirement:** None			P	In	Proper non-antibiotics feeds
				F	In	Ability to use and buy non-antibiotics
5.3.2	**Indicator:** Allowance for the use of antibiotics categorized as critically important by the World Health Organization (WHO), even if authorized by the pertinent national authorities	H	Knowledge on what not to use	H	In	
				S	Out	Criteron has no reference to social conditions
				N	Out	Criteron has no reference to natural conditions
	**Requirement:** None			P	In	Proper non-antibiotics feeds
				F	In	Ability to use and buy non-antibiotics
5.3.3	**Indicator:** Information on chemical storage and usage.	H	Documentation	H	In	
				S	Out	Criteron has no reference to social conditions
				N	Out	Criteron has no reference to natural conditions
	**Requirement:** Records of stocks and usage are available for all products.			P	Out	Criteron has no reference to physical conditions
				F	In	Hiring assistant to record
5.3.4	**Indicator:** Proper use of chemical products by farm workers	H	Training	H	In	
				S	Out	Criteron has no reference to social conditions
				N	Out	Criteron has no reference to natural conditions

	**Requirement:** Evidences of worker awareness / training and instructions are available			P	Out	Criteron has no reference to physical conditions
				F	Out	Criteron has no reference to financial conditions
5.3.5	**Indicator:** Allowance for treating water with pesticides banned or restricted by the Rotterdam Convention on Prior Informed Consent (PIC), the Stockholm Convention on Persistent Organic Pollutants (POPs) or classed as "extremely hazardous" or "highly hazardous" (classes Ia and Ib) by the World Health Organization (WHO).<br><br>**Requirement:** None	H	Knowledge on what not to use	H	In	
				S	Out	Criteron has no reference to social conditions
				N	Out	Criteron has no reference to natural conditions
				P	In	Availability of allowed pesticides for treating water
				F	Out	Criteron has no reference to financial conditions
5.3.6	**Indicator:** Allowance for discharge of any hazardous chemicals without previous neutralization.<br>**Requirement:** None	P	Container	H	In	Knowledge on procedures for neutralization
				S	Out	Criteron has no reference to social conditions
				N	Out	Criteron has no reference to natural conditions
				P	In	
				F	In	Ability to build or install proper discharge system
5.3.7	**Indicator:** Use of probiotic bacterial strains excluding the use of fermented product to seed further batches<br>**Requirement:** Only probiotic products approved by the appropriate competent authorities can be used.	P	Approved probiotic	H	In	Knowledge
				S	Out	Criteron has no reference to social conditions
				N	In	
				P	Out	Criteron has no reference to physcial conditions
				F	In	Ability to buy approved probiotic products
**PRINCIPLE 6: MANAGE BROODSTOCK ORIGIN, STOCK SELECTION AND EFFECTS OF STOCK MANAGEMENT**<br>Criterion 6.1: Presence of exotic or introduced shrimp species						
6.1.1	**Indicator:** Use of non-indigenous shrimp species.<br>**Requirement:** Allowed, provided it is in commercial production locally AND there is no evidence of establishment or impact on adjacent ecosystems by that species AND there is documentation (hatchery permits, import licenses, etc.) that demonstrates	N		H	Out	Criteron has no reference to human conditions
				S	Out	Criteron has no reference to social conditions
				N	In	
				P	Out	Criteron has no reference to physical conditions
				F	In	Ability to buy proper approved species

	compliance with introduction procedures as identified by regional, national and international importation guidelines (e.g., OIE and ICES).					
6.1.2	**Indicator:** Prevention measures in place to prevent escapes at harvest and during grow-out include (A-F): A. Effective screens or barriers of appropriate mesh size for the smallest animals present; double screened when non-indigenous species. **Requirement:** Yes	P	Screens or barriers	H	Out	Criteron has no reference to human conditions
				S	Out	Criteron has no reference to social conditions
				N	Out	Criteron has no reference to natural conditions
				P	In	
				F	In	Ability to buy and install proper screens or barriers
	B. Perimeter pond banks or dykes are of adequate height and construction to prevent breaching in exceptional flood events. **Requirement:** Yes	P	Infrastructure	H	Out	Criteron has no reference to human conditions
				S	Out	Criteron has no reference to social conditions
				N	Out	Criteron has no reference to natural conditions
				P	In	
				F	In	Paid labour on construction of ponds banks or dykes
	C. Regular, timely inspections are performed and recorded in a permanent register **Requirement:** Yes	H	Monitoring	H	In	
				S	Out	Criteron has no reference to social conditions
				N	Out	Criteron has no reference to natural conditions
				P	Out	Criteron has no reference to physcial conditions
				F	In	Hiring assistant to record
	D. Timely repairs to the system are recorded **Requirement:** Yes	H	Documentation	H	In	
				S	Out	Criteron has no reference to social conditions
				N	Out	Criteron has no reference to natural conditions
				P	Out	Criteron has no reference to physcial conditions
				F	In	Hiring assistant to record
	E. Installation and management of trapping devices to sample for the existence of escapes; data is recorded **Requirement:** Yes	H	Documentation	H	In	
				S	Out	Criteron has no reference to social conditions
				N	Out	Criteron has no reference to natural conditions
				P	Out	Criteron has no reference to physcial conditions

				F	In	Hiring assistant to record
	F. Escape recovery protocols in place.<br><br>**Requirement:** Yes	H	Management (of protocols)	H	In	
				S	Out	Criteron has no reference to social conditions
				N	Out	Criteron has no reference to natural conditions
				P	Out	Criteron has no reference to physcial conditions
				F	Out	Criteron has no reference to financial conditions
6.1.3	**Indicator:** Escapes and actions taken to prevent reoccurrence.<br><br>**Requirement:** Records are available for inspection.	H	Management	H	In	
				S	Out	Criteron has no reference to social conditions
				N	Out	Criteron has no reference to natural conditions
				P	Out	Criteron has no reference to physcial conditions
				F	Out	Criteron has no reference to financial conditions
Criterion 6.2: Origin of postlarvae or broodstock						
6.2.1	**Indicator:** PL and broodstock have appropriate disease-free status and sources meet regional, national and international importation guidelines (e.g., OIE and ICES)<br>**Requirement:** Documentation provided demonstrating compliance within two years of standard's publication date for wild monodon broodstock sourced locally; applicable immediately in all other cases	H	Documentation	H	In	
				S	Out	Criteron has no reference to social conditions
				N	Out	Criteron has no reference to natural conditions
				P	Out	Criteron has no reference to physical conditions
				F	Out	Criteron has no reference to financial conditions
6.2.2	**Indicator:** Percent of total postlarvae from closed loop hatchery (i.e., farm-raised broodstock)<br><br>**Requirement:** P. vannamei, P. indicus, P. stylirostris 100% P. monodon must be increased over time, and reach 100% within six years after the publication of the standard.	N	Postlarvae	H	Out	Criteron has no reference to human conditions
				S	Out	Criteron has no reference to social conditions
				N	In	
				P	Out	Criteron has no reference to physical conditions
				F	In	Abilty to buy proper stocks
6.2.3	**Indicator:** Origin of wild-caught broodstock	N	Broodstock	H	Out	Criteron has no reference to human conditions

	**Requirement:** Sourced from locally fished broodstock only.			S	Out	Criteron has no reference to social conditions
				N	In	
				P	Out	Criteron has no reference to physical conditions
				F	In	Abilty to buy from proper source
6.2.4	**Indicator:** Allowance for wild-caught PL other than natural tidal flow into ponds<br><br>**Requirement:** None	N		H	Out	Criteron has no reference to human conditions
				S	Out	Criteron has no reference to social conditions
				N	In	
				P	Out	Criteron has no reference to physical conditions
				F	Out	Criteron has no reference to financial conditions
Criterion 6.3: Transgenic shrimp						
6.3.1	**Indicator:** Allowance for the culture of transgenic shrimp (including the offspring of genetically engineered shrimp)<br>**Requirement:** None	H	Management	H	In	
				S	Out	Criteron has no reference to social conditions
				N	Out	Criteron has no reference to natural conditions
				P	Out	Criteron has no reference to physical conditions
				F	Out	Criteron has no reference to financial conditions
**PRINCIPLE 7: USE RESOURCES IN AN ENVIRONMENTALLY EFFICIENT AND RESPONSIBLE MANNER**<br>Criterion 7.1 - Traceability of raw materials in feed						
7.1.1	**Indicator:** Evidence of basic traceability of feed ingredients, including source, species, country of origin and harvest method demonstrated by the feed producer.<br><br>**Requirement:** List of all ingredients making up more than 2% of the feed available provided on company letterhead.	S	Supplier can provide document	H	Out	Criteron has no reference to human conditions
				S	In	
				N	Out	Criteron has no reference to natural conditions
				P	Out	Criteron refers to physical conditions but focus is on traceability
				F	Out	Criteron has no reference to financial conditions
7.1.2	**Indicator:** Demonstration of chain of custody and traceability for fisheries products in feed through an ISEAL member or ISO 65	S	Supplier can provide document	H	Out	Criteron has no reference to human conditions
				S	In	

	compliant certification scheme that also incorporates the FAO Code of Conduct for Responsible Fisheries.<br>**Requirement:** Yes			N	Out	Criteron has no reference to natural conditions
				P	Out	Criteron refers to physical conditions but focus is on traceability
				F	Out	Criteron has no reference to financial conditions
Criterion 7.2 - Origin of aquatic and terrestrial feed ingredients						
7.2.1a	**Indicator:** Timeframe for 100% (mass balance) fishmeal and fish oil used in feed to come from fisheries certified by a full ISEAL member that has guidelines specifically promoting ecological sustainability of forage fisheries<br>**Requirement:** Within five years following the date of standards publication	H	Planning, management	H	In	
				S	In	Connections with certified fishmeal seller
				N	Out	Criteron has no reference to natural conditions
				P	In	Certified feed
				F	In	Ability to buy certified feed
Criterion 7.2 - Origin of aquatic and terrestrial feed ingredients						
7.2.1b	**Indicator:** FishSource score, for the fishery(ies) from which a minimum of 80% of the fishmeal and fish oil by volume is derived (See Appendix IV, subsection 3 for explanation of FishSource scoring) a. for Fishsource Criteria 4 (spawning biomass assessment) b. for Fishsource Criteria 1, 2, 3 and 5<br>**Requirement:** a. 8<br>b. 6 or compliance with alternative interim proposal 7.1.1c	H	Knowledge	H	In	
				S	Out	Criteron has no reference to social conditions
				N	Out	Criteron has no reference to natural conditions
				P	In	Feed with specific FishSource score
				F	In	Ability to buy specific FishSource score
7.2.1c	**Indicator:** Lacking a FishSource assessment a fishery could be engaged in an Improvers Programme. (transparent and public Fisheries Improvement Project (FIP) with periodic public reporting (refer to Appendix VII).<br>**Requirement:** See Appendix VII for details on compliance	H		H	In	
				S	In	Engage in collective improvement program with other farmers
				N	Out	Criteron has no reference to natural conditions
				P	Out	Criteron has no reference to physical conditions
				F	Out	Criteron has no reference to financial conditions
7.2.2	**Indicator:** Percentage of non-marine ingredients from sources certified by an ISEAL member's certification scheme that addresses environmental and social sustainability	P		H	In	Knowledge on ingredients, regulations, Calculation
				S	Out	Criteron has no reference to social conditions

	**Requirement:** 80% for soy and palm oil within five years from the date of the ASC Shrimp Standard's publication			N	Out	Criteron has no reference to natural conditions
				P	In	
				F	Out	Criteron has no reference to financial conditions
Criterion 7.3: Use of genetically modified (GM) ingredients in feed						
7.3.1	**Indicator:** Allowance for feed containing ingredients that are genetically modified ONLY when information regarding the use of GM ingredients in shrimp feed is made easily available to retailers and end consumers, including:a. Disclosure on the audit reports if GMO ingredients were used in the feed fed to shrimpb. Disclosure if GMO ingredients were used in the feed fed to ASC-certified shrimp all along the supply chain up to the retailer. Total disclosure on therevised auditor reports are published on an easy-access database on the ASC web page (when available). This database, when available, should be made available on demand to retailer and consumers.c. Use of the most adequate, fast and user-friendly communication tools to inform retailers and consumers on all certified products<br>**Requirement:** Yes	H	Documentation	H	In	
				S	In	Seller can provide the document
				N	Out	Criteron has no reference to natural conditions
				P	In	Availability of feed with info on GMO
				F	In	Ability to buy proper feed ingredients
7.3.2	**Indicator:** List (footnote) of feed ingredients does not contain any GMO<br>**Requirement:** Yes	P	Availability of non-GMO feed	H	In	Knowledge on GMO
				S	Out	Criteron has no reference to social conditions
				N	Out	Criteron has no reference to natural conditions
				P	In	
				F	Out	Ability to buy proper feed ingredients
7.3.3	**Indicator:** Non-GMO feed traceability by the feed producer and on the farm<br>**Requirement:** Yes	S	Documentation by feed producer	H	In	
				S	In	
		H	Documentation	N	Out	Criteron has no reference to natural conditions
				P	Out	Criteron refers to physical conditions but focus is on traceability
				F	Out	Criteron has no reference to financial conditions

7.3.4	**Indicator:** Samples taken randomly by the auditor are tested negative by PCR<br><br>**Requirement:** Yes	H	Testing	H	In	
				S	Out	Criteron has no reference to social conditions
				N	Out	Criteron has no reference to natural conditions
				P	Out	Criteron has no reference to physical conditions
				F	Out	Criteron has no reference to financial conditions
Criterion 7.4: Efficient use of wild fish [136] for fishmeal and oil						
7.4.1	**Indicator:** Feed Fish Equivalence Ratio (FFER) L. vannamei and P. monodon<br><br>**Requirement:** L. vannamei ≤1.35:1 and P. monodon ≤1.9: 1	P		H	In	Calculation
				S	Out	Criteron has no reference to social conditions
				N	Out	Criteron has no reference to natural conditions
				P	In	
				F	Out	Criteron has no reference to financial conditions
7.4.2a	**Indicator:** Economic Feed Conversation Ratio (eFCR)<br><br>**Requirement:** Records are available	H	Documentation	H	In	
				S	Out	Criteron has no reference to social conditions
				N	Out	Criteron has no reference to natural conditions
				P	Out	Criteron has no reference to physical conditions
				F	Out	Criteron has no reference to financial conditions
7.4.2b	**Indicator:** Protein Retention Efficiency<br><br>**Requirement:** Records are available	H	Documentation	H	In	Knowledge on proper ratio
				S	Out	Criteron has no reference to social conditions
				N	Out	Criteron has no reference to natural conditions
				P	Out	Criteron has no reference to physical conditions
				F	Out	Criteron has no reference to financial conditions
Criterion 7.5: Effluent contaminant load						
7.5.1	**Indicator:** Nitrogen effluent load per ton of shrimp produced over a 12-month period.	P		H	In	Calculation
				S	Out	Criteron has no reference to social conditions

	**Requirement:** Less than 25.2 kg N per ton of shrimp for L. vannamei. Less than 32.4 kg N per ton of shrimp for P. monodon.			N	Out	Criteron has no reference to natural conditions
				P	In	
				F	Out	Criteron has no reference to financial conditions
7.5.2	**Indicator:** Phosphorous effluent load per ton of shrimp produced over a 12-month period.<br><br>**Requirement:** Less than 3.9 kg P per ton of shrimp for L. vannamei. Less than 5.4 kg P per ton of shrimp for P. monodon.	P		H	In	Calculation
				S	Out	Criteron has no reference to social conditions
				N	Out	Criteron has no reference to natural conditions
				P	In	
				F	Out	Criteron has no reference to financial conditions
7.5.3	**Indicator:** Responsible handling and disposal of sludge and sediments removed from ponds and canals.<br><br>**Requirement:** No discharge or disposal of sludge and sediments to public waterways and wetlands.	H	Management	H	In	
				S	In	Social norm
				N	Out	Criteron has no reference to natural conditions
				P	In	Infrastructure
				F	Out	Criteron has no reference to financial conditions
7.5.4	**Indicator:** Treatment of effluent water from permanently aerated ponds.<br><br>**Requirement:** Evidence that all discharged water goes through a treatment system, and concentration of settleable solids in effluent water < 3.3 mL/L.	P	Infrastructure	H	In	Managment of effluent water treatment plan, documentation
				S	Out	Criteron has no reference to social conditions
				N	Out	Criteron has no reference to natural conditions
				P	In	
				F	In	Ability to build proper system
Criterion 7.6: Energy efficiency						
7.6.1	**Indicator:** Energy consumption by sources over a 12-month period.<br><br>**Requirement:** Records available for all activities.	H	Documentation	H	In	
				S	Out	Criteron has no reference to social conditions
				N	Out	Criteron has no reference to natural conditions
				P	In	Availability og enery
				F	Out	Criteron has no reference to financial conditions
7.6.2		H	Documentation	H	In	

	**Indicator:** Annual Cumulative Energy Demand (megajoules/ton of shrimp produced) over a 12-month period.<br>**Requirement:** Records available for verification of calculations.			S	Out	Criteron has no reference to social conditions
				N	Out	Criteron has no reference to natural conditions
				P	In	Availability og enery
				F	Out	Criteron has no reference to financial conditions
Criterion 7.7: Handling and disposal of hazardous materials and wastes						
7.7.1	**Indicator:** Safe storage and handling of chemicals and hazardous materials.<br>**Requirement:** Evidence of procedures in place.	P	Storage	H	In	Management
				S	Out	Criteron has no reference to social conditions
				N	Out	Criteron has no reference to natural conditions
				P	In	
				F	In	Ability to build proper storage
7.7.2	**Indicator:** Responsible handling and disposal of wastes based on risk assessment and possibilities of recycling.<br>**Requirement:** Evidence of procedures in place.	P	Infrastructure	H	In	Managment of disposal waste
				S	Out	Criteron has no reference to social conditions
				N	Out	Criteron has no reference to natural conditions
				P	In	
				F	In	Ability to build proper system
