# Supplementary material for: A capability approach to assess aquaculture sustainability standard compliance
Source: PLoS One. 2020 Jan 23;15(1):e0227812. doi: 10.1371/journal.pone.0227812 (PMC6977730; doi:10.1371/journal.pone.0227812)
Supplement: S3 Table — (PDF) [file pone.0227812.s003.pdf]

S3 Table. Assessment of prescribed capitals and bundles of capitals for the Southeast Asian Shrimp Aquaculture Improvement Protocol level one standard.

SEASAIP Level 1						
**Criteria**	**Requirements**	**Prescribed capitals**	**Justification**	**Bundle of capitals**	**In/out**	**Justification (Bundle of capitals)**
1. Traceability						
1.1 Legal compliance for farm operations -	Evidence of operational legality via certificate, legal document, or other applicable evidence.	H	Documentation	H	In	
				S	Out	Criteron has no reference to social conditions
				N	In	Ownership right of lands or land lease
				P	Out	Criteron has no reference to physical conditions
				F	Out	Criteron has no reference to financial conditions
1.2 Compliance with Country Good Aquaculture Practice Standard for Shrimp or ASEAN Shrimp GAP	Evidence of compliance with the requirements of the relevant scheme if applicable.	H	Documentation	H	In	
				S	Out	Criteron has no reference to social conditions
				N	Out	Criteron has no reference to natural conditions
				P	Out	Criteron has no reference to physical conditions
				F	Out	Criteron has no reference to financial conditions
1.3 Farm Profile Document	A farm profile document is available and must include, at a minimum: name of farm, owner and/or manager, membership to farming cooperative or other farmers' union if applicable, GPS coordinates, aerial map,	H	Documentation	H	In	
				S	In	Social networks, cooperatives facilitate in preparing documents
				N	Out	Criteron has no reference to natural conditions

	farm size, number of ponds, production volume, and species farmed.			P	Out	Criteron has no reference to physical conditions
				F	Out	Criteron has no reference to financial conditions
1.4 Identification of Government Enforcement Officials	The name and contact information (e.g. telephone number and/or e-mail address) of the relevant authorities can be identified	H	Documentation	H	In	
				S	In	Social network for contact info
				N	Out	Criteron has no reference to natural conditions
				P	Out	Criteron has no reference to physical conditions
				F	Out	Criteron has no reference to financial conditions
1.5 Effective system of segregation between compliant and non- compliant products on the farm	A system must be established to avoid mixing compliant and non-compliant products via physical identification or product handling procedures, including the relevant records	P	Infrastructure	H	In	Managment
				S	Out	Criteron has no reference to social conditions
				N	Out	Criteron has no reference to natural conditions
				P	In	
				F	In	Financial capital to develop or install the system
1.6 Broker License and/or Registration (if applicable)	Brokers must have a license or be registered with the government, or be an approved supplier for a processor	H	Documentation	H	In	
				S	In	Social network with proper broker, broker can prepare documents
				N	Out	Criteron has no reference to natural conditions
				P	Out	Criteron has no reference to physical conditions
				F	Out	Criteron has no reference to financial conditions
		H	Documentation	H	In	

1.7 Effective system to identify different batches of product from different farms	Broker will have to provide a written description to the farm about how they differentiate different products from different farms.			S	In	Social network with proper broker, broker can prepare documents
				N	Out	Criteron has no reference to natural conditions
				P	Out	Criteron has no reference to physical conditions
				F	Out	Criteron has no reference to financial conditions
1.8 Movement of Harvested Shrimp	Movement/sale of all harvested shrimp shall be recorded	H	Documentation	H	In	
				S	In	Buyers or brokers facilitate in reocrding, provide traceability
				N	Out	Criteron has no reference to natural conditions
				P	Out	Criteron has no reference to physical conditions
				F	In	Hiring assistant to record
1.9 Data Recording and availability	Data relevant to compliance criteria in this section are collected using robust methods and are available for inspection. Collected data must be available for inspection by auditors and/or ASIC upon request.	H	Documentation	H	In	
				S	In	Social networks, cooperatives facilitate in document preparation
				N	Out	Criteron has no reference to natural conditions
				P	Out	Criteron has no reference to physical conditions
				F	In	Hiring assistant to record
2. Shrimp Health Management						
2.1 Monitoring of shrimp health and disease prevalence	• The farm must routinely examine shrimp for health status and disease prevalence<br>• Records of sampling for disease prevalence inside farm and records noting	H	Monitoring	H	In	
				S	In	

	tests that indicate the presence of disease and detailing subsequent actions					
	• Records for all mortalities on farm (except days of allowable/normal/expected mortalities)			N	Out	Criteron has no reference to natural conditions
	• Records of causation analysis and corrective actions	S	communication with neighbours	P	In	Devices, equipment for simple testing, monitoring
	• Records show that farmer informed the relevant authority upon evidence of outbreak			F	In	Hiring assistant to record
	• Records show that farmers are in regular communication with the farmers surrounding the farm regarding disease occurrence and prevention					
2.2 Monitoring of water quality	The farm must examine and record, at least once weekly, the water quality in shrimp ponds, including, at a minimum: temperature, dissolved oxygen, salinity, pH, and ammonia.	H	Monitoring	H	In	
				S	Out	Criteron has no reference to social conditions
				N	Out	Criteron has no reference to natural conditions
				P	In	Devices, equipment for simple testing, monitoring
				F	In	Hiring assistant to record
2.3 Handling and disposal of dead/infected shrimp and pond water	• Must demonstrate proper disposal of dead/infected shrimp	P	Devices, proper discharge system	H	In	Managment, knowledge in proper farming practices
				S	Out	Criteron has no reference to social conditions
	• Must demonstrate how the affected pond water was treated prior to discharge outside of the farm boundary			N	Out	Criteron has no reference to natural conditions
				P	In	
				F	Out	Criteron has no reference to financial conditions

2.4 Use of antibiotics	• Antibiotics highly or critically important for human health are prohibited<br>• Prophylactic use of antibiotics of any kind is prohibited<br>• Every use of antibiotics at any time must be recorded, including the product name, dose, and route of administration.	H	Knowledge on antibiotics	H	In	
				S	In	Sharing knowledge with neighbours or association about antibiotics
				N	Out	Criteron has no reference to natural conditions
				P	In	Ability to replace critical antibiotics
				F	In	Ability to purchasenon-antibiotics, Hiring assistant to record
2.5 Use of veterinary drugs and chemicals	• The use of drugs or chemicals banned by the country of production, importing country, or the country of export is prohibited<br>• Use of trained staff to administer drugs (i.e. therapeutants) and chemicals according to the instructions prescribed by the manufacturer<br>• Every use of therapeutic drugs and chemicals must be recorded, including the product name, dose, and route of administration<br>• Any product used for pond preparation must be recorded by product name and sales company/ agent<br>• The production is allowed a maximum of 1 chemical treatment per production cycle	H	Knowledge on regulations	H	In	
			Training	S	In	Social networks facilitate knowledge sharing on regualtions on prohibited chemicals
			Documentation	N	Out	Criteron has no reference to natural conditions
				P	Out	Criteron has no reference to physical conditions
				F	In	Hiring assistant to record
2.6 Use of probiotics and other bioremediation agents	Must have records of use for probiotics and other bioremediation agents (type and dose)	H	Recording	H	In	
				S	Out	Criteron has no reference to social conditions

				N	Out	Criteron has no reference to natural conditions
				P	Out	Criteron has no reference to physical conditions
				F	In	Hiring assistant to record
2.7 Implementation of basic biosecurity protocols	Existence of a written health management plan that includes, at a minimum, disease monitoring procedures, disease response, active vector or boundary controls, and disposal of mortalities.	H	Knowledge, management	H	In	
				S	In	Social networks facilitate knowledge sharing and help, learning from other people's example
				N	Out	Criteron has no reference to natural conditions
				P	Out	Criteron has no reference to physical conditions
				F	In	Hiring assistant to make a proper management plan
2.8 Data recording and availability	Data relevant to compliance criteria in this section are collected using robust methods and are available for inspection. Collected data must be available for	H	Documentation	H	In	
				S	Out	Criteron has no reference to social conditions
				N	Out	Criteron has no reference to natural conditions
				P	Out	Criteron has no reference to physical conditions
				F	In	Hiring assistant to record
3. Source of Stock						
3.1 Sourcing fry or post larvae from wild sources	Prohibited except for passive settlement in extensive systems.	N		H	In	Knowledge on policy, regulations
				S	Out	Criteron has no reference to social conditions
				N	In	
				P	Out	Criteron has no reference to physical conditions

				F	In	Ability to purchase other types of fry
3.2 Movement documents/receipts for seed	Receipts for seed are required from the seed supplier. If farm uses a broker, the farm must have movement documents/receipts from the broker that show the origin of the seed.	H	Documentation	H	In	
				S	In	Supplier can provide the documents
				N	Out	Criteron has no reference to natural conditions
				P	Out	Criteron has no reference to physical conditions
				F	Out	Criteron has no reference to financial conditions
3.3 Identification of seed providers	The farm has a name and contact information for any seed company that is used.	H	Documentation	H	In	
				S	In	Supplier can provide the documents
				N	Out	Criteron has no reference to natural conditions
				P	Out	Criteron has no reference to physical conditions
				F	Out	Criteron has no reference to financial conditions
3.4 Sourcing of post larvae	Farmers must source seed that is compliant with Section 7.	H	Knowledge on certified sources	H	In	
				S	Out	Criteron has no reference to social conditions
				N	Out	Criteron has no reference to natural conditions
				P	Out	Criteron has no reference to physical conditions
				F	In	Financial capital to buy from other allowed sources
3.5 Data recording and availability	Data relevant to compliance criteria in this section are collected using robust methods and are available for inspection. Collected	H	Data collection	H	In	
				S	In	Supplier can provide the documents
				N	Out	Criteron has no reference to natural conditions

	data must be available for inspection by auditors and/ or ASIC upon request.			P	Out	Criteron has no reference to physical conditions
				F	Out	Criteron has no reference to financial conditions
4. Feed Sourcing and Management						
4.1 Use of commercial pelleted feed	Commercial pelleted feed must be purchased from manufacturers/sellers/ importers that have an operational license from the national government.	H	Knowledge on regulations	H	In	
				S	In	Social networks and contacts with proper sellers
				N	Out	Criteron has no reference to natural conditions
				P	Out	Criteron has no reference to physical conditions
				F	In	Ability to buy from certified sources
4.2 Use of approved additives and supplements	Feed additives and supplements must be legal/registered. Their use, including name/type and dose, must be identified and recorded.	P	Proper feed	H	In	
				S	Out	Criteron has no reference to social conditions
				N	Out	Criteron has no reference to natural conditions
		H	Documentation	P	In	
				F	In	Ability to buy legal feed additives and supplements
4.3 Economic Feed Conversion Ratio (L. vannamei)	Shall be less than 2.	H	Caculation	H	In	Knowledge in caculating ratio
				S	Out	Criteron has no reference to social conditions
				N	Out	Criteron has no reference to natural conditions
				P	Out	Criteron has no reference to physical conditions
				F	Out	Criteron has no reference to financial conditions

4.4 Movement documents/receipts for feed	Receipts for feed purchases are required from the feed supplier. If farm uses a broker, the farm must have movement documents/receipts from the broker that show the origin of the feed.	H	Documentation	H	In	
				S	In	Supplier can provide the documents
				N	Out	Criteron has no reference to natural conditions
				P	Out	Criteron has no reference to physical conditions
				F	Out	Criteron has no reference to financial conditions
4.5 Identification of feed providers	The farm has a name and contact information for any feed company that is used.	H	Documentation	H	In	
				S	In	Networks and contacts with seed company
				N	Out	Criteron has no reference to natural conditions
				P	Out	Criteron has no reference to physical conditions
				F	Out	Criteron has no reference to financial conditions
4.6 Certified feed use	Farmers must use feed that is compliant with Section 8.	P		H	In	Knowledge on regulations
				S	In	Social networks and contacts with proper feed company
				N	Out	Criteron has no reference to natural conditions
				P	In	
				F	In	Ability to buy from certified sources
4.7 Data Recording and availability	Data relevant to compliance criteria in this section are collected using robust methods and are available for inspection. Collected data must be available for inspection by auditors and/or ASIC upon request.	H	Data collection	H	In	
				S	In	Supplier can provide the documents
				N	Out	Criteron has no reference to natural conditions
				P	Out	Criteron has no reference to physical conditions
				F	Out	Criteron has no reference to financial conditions

**5. Environmental Impact Management**						
5.1 Mangrove and Habitat Impacts						
5.1.2 Siting in mangroves and other high value habitat	• Farms must not have been sited/built in mangrove ecosystems or other high value habitat after 1999 (or earlier according to national regulations, i.e., the Philippines).<br>• GPS coordinates of the farm site are recorded and farms have a document indicating land use from 1999 to the establishment of the farm or historical land use maps/statements. Farms can provide a statement indicating the year of construction of ponds. Farms shall follow local and national regulations about coastal spatial plan if applicable	N		H	In	
				S	Out	Criteron has no reference to social conditions
		H	Documentation	N	In	
				P	In	GPS coordinates system
				F	In	Ability to relocate to new location
5.1.3 Expansion of farms in high value habitat	Prohibited, except for canals, which must provide evidence of successful restoration activity	N		H	In	Restoration activity
				S	In	Social networks, collective actions facilitate restoration activity
				N	In	
				P	Out	Criteron has no reference to physical conditions
				F	In	Ability to relocate to new location, ability to pay for restoration activity
5.1.4 Siting in Protected Areas (PA)	There is evidence that the farm site or related facilities are not within a national or international Protected Area, unless	N	Farm location	H	In	Documentation
				S	Out	Criteron has no reference to social conditions

	permitted by the relevant authorities and if an effective management plan exists.			N	In	
				P	Out	Criteron has no reference to physical conditions
				F	In	Ability to relocate to new location
5.1.5 Cumulative Impacts	Any expansion of farms into habitat not previously converted (see 5.1.2 for mangrove exclusions) <u>must consider their contribution to cumulative impacts</u>. This requirement is not applicable for expansion into areas already in use for agriculture. <u>Farms can consider their impact via the use of Environmental Impact Assessment</u>	H	Management	H	In	
				S	Out	Criteron has no reference to social conditions
				N	Out	Criteron has no reference to natural conditions
				P	Out	Criteron has no reference to physical conditions
				F	In	Paid experts to conduct Environmental Impact Assessment
5.2 Use and Discharge of Water						
5.2.1 Use of <u>fresh groundwater</u> (below 5ppt)	• <u>Shall not be used in the ponds</u>.<br><br>• If permitted under national regulations, record of use of fresh groundwater must be kept, and the potential impacts (salinity of surrounding wells and reduced freshwater availability) must be assessed and recorded.	H	Knowledge on policy	H	In	
				S	Out	Criteron has no reference to social conditions
				N	In	Availability of water from other sources
				P	Out	Criteron has no reference to social conditions
				F	Out	Criteron has no reference to financial conditions
5.2.2 Discharge of saline water into natural freshwater bodies and agricultural lands	The farm is designed and managed <u>to ensure that saline water cannot be discharged into freshwater bodies</u> or agricultural lands.	P	Barriers	H	In	Designing and managing
				S	Out	Criteron has no reference to social conditions
				N	Out	Criteron refers to natural conditions but focus in on discharing system
				P	In	
				F	In	Building proper barriers
		P		H	In	

5.2.3 Discharge of water from the farm	• Daily average water exchange per farm shall not exceed 10% of pond volume, calculated over the entire production cycle<br>• Records of all water discharge and exchange must be kept		Infrastructure, equipment	S	Out	Criteron has no reference to social conditions
				N	Out	Criteron has no reference to natural conditions
		H	Caculation, documents	P	In	
				F	In	Ability to buy, install proper equipment
5.2.4 Effluent water quality monitoring	• Effluent water quality must be tested and recorded over all periods of the production cycle<br>• Records of effluent water quality testing demonstrate compliance with relevant laws and regulations (if applicable)<br>• Visual inspection of sampling procedure confirms testing produces accurate results	H	Testing, monitoring	H	In	
				S	Out	Criteron has no reference to social conditions
				N	Out	Criteron has no reference to natural conditions
				P	Out	Criteron has no reference to social conditions
				F	In	Hiring assistant for testing and recording
5.2.5 Disposal of sludge	Dredged sediment from canals, watercourses and ponds is properly contained and/or located to prevent the salinization of soil and groundwater, and does not cause other significant ecological impacts to receiving and/or surrounding environments.	P	Container	H	Out	Criteron has no reference to human conditions
				S	Out	Criteron has no reference to social conditions
				N	Out	Criteron refers to natural conditions but focus in on discharing system
				P	In	
				F	Out	Ability to build proper container, hiring assistant to contain sediment
5.2.6 Potential impact of effluent water	• Effluent water must be treated if the water quality poses a significant risk of impact to the receiving water<br>• Records of effluent treatment/water quality control prior to discharge	P	Infrastructure	H	In	
				S	Out	Criteron has no reference to social conditions
				N	Out	Criteron refers to natural conditions but focus in on discharing system
		H	Documentation	P	In	

				F	Out	Criteron has no reference to financial conditions
5.2.7 Data recording and availability	Data relevant to compliance criteria in this section are collected using robust methods and are available for inspection. Collected data must be available for inspection by auditors and/ or ASIC upon request.	H	Data collection	H	In	
				S	Out	Criteron has no reference to social conditions
				N	Out	Criteron has no reference to natural conditions
				P	Out	Criteron has no reference to physical conditions
				F	Out	Criteron has no reference to financial conditions
5.3 Predator Control						
5.3.1 Predator control	Active lethal predator (birds, mammals, reptiles) control is prohibited.	P	Equipment	H	In	Skills on (natural) predator control
				S	Out	Criteron has no reference to social conditions
				N	Out	Criteron has no reference to natural conditions
				P	In	
				F	Out	Criteron has no reference to financial conditions
5.3.2 Protection of listed species	Farming activities must not cause mortality of any threatened or endangered species, as listed by the IUCN	H	Management	H	In	
				S	Out	Criteron has no reference to social conditions
				N	Out	Criteron has no reference to natural conditions
				P	Out	Criteron has no reference to physical conditions
				F	Out	Criteron has no reference to financial conditions
		H	Documentation	H	In	

5.3.3 Records of predator mortalities	Any wildlife/ predator mortalities that occur on the farm, regardless of the reason for the incident must be recorded including the common name of the species, number of mortalities, and cause of mortality.			S	Out	Criteron has no reference to social conditions
				N	Out	Criteron has no reference to natural conditions
				P	Out	Criteron has no reference to physical conditions
				F	In	Hiring assistant to record
5.3.4 Data recording and availability	Data relevant to compliance criteria in this section are collected using robust methods and are available for inspection. Collected data must be available for inspection by auditors and/ or ASIC upon request	H	Documentation	H	In	
				S	Out	Criteron has no reference to social conditions
				N	Out	Criteron has no reference to natural conditions
				P	Out	Criteron has no reference to physical conditions
				F	In	Hiring assistant to record
5.4 Escape Management						
5.4.1 Stocking records	The number of shrimp stocked, their average weight, and total biomass must be recorded at stocking and at harvest.	H	Documentation	H	In	
				S	Out	Criteron has no reference to social conditions
				N	Out	Criteron has no reference to natural conditions
				P	Out	Criteron has no reference to physical conditions
				F	In	Hiring assistant to record
5.4.2 Escape prevention	• Farm shall employ appropriate measures to prevent the escape of cultured shrimp, including secondary containment at harvest.<br>• Appropriate escape prevention measures in place must include double screens or secondary catchment mechanisms on outlet	P	Equipment, infrastructure	H	In	Management
				S	Out	Criteron has no reference to social conditions
				N	Out	Criteron has no reference to natural conditions
				P	In	

	gates that are inspected and maintained regularly, and records of inspection (with any maintenance activity if enacted) are kept.			F	In	Ability to buy equipments and install prevention measures
5.4.3 Escape reporting	• In the event of a large escape, relevant authorities, including ASIC, must be informed	S	Communication with authorities	H	In	
				S	In	
	• Records (size of animal estimated number of escapees, their size, and estimated recapture if applicable) of any escape event must be kept	H		N	Out	Criteron has no reference to natural conditions
				P	Out	Criteron has no reference to physical conditions
				F	Out	Criteron has no reference to financial conditions
5.4.4 Data recording and availability	Data relevant to compliance criteria in this section are collected using robust methods and are available for inspection. Collected data must be available for inspection by auditors and/ or ASIC upon request.	H	Data collection	H	In	
				S	Out	Criteron has no reference to social conditions
				N	Out	Criteron has no reference to natural conditions
				P	Out	Criteron has no reference to physical conditions
				F	In	Hiring assistant to record
6. **Socio-economic aspects**						
6.1 General Working Conditions						
6.1.1 Child labor	• No hired workers under the minimum age according to national regulations and ILO	H	Management	H	In	
				S	Out	Criteron has no reference to social conditions
	• Exception: In the case of family businesses (apprenticeships), children who are immediate family members must not be			N	Out	Criteron has no reference to natural conditions
				P	Out	Criteron has no reference to physical conditions

	engaged in hazardous work and work that jeopardizes schooling			F	Out	Criteron has no reference to financial conditions
6.1.2 Farm worker agreement/ contract	Workers are covered with a lawful farm worker agreement (written or verbal [unless specified by law as written]) specifying the duration of work and remuneration package/income sharing arrangements.	H	Management	H	In	
				S	Out	Criteron has no reference to social conditions
				N	Out	Criteron has no reference to natural conditions
				P	Out	Criteron has no reference to physical conditions
				F	Out	Criteron has no reference to financial conditions
6.1.3 Termination conditions	Workers are free to terminate their employment and receive full payment until the last day of their employment, based on reasonable notice given to their employer (according to national law)	H	Management	H	In	
				S	Out	Criteron has no reference to social conditions
				N	Out	Criteron has no reference to natural conditions
				P	Out	Criteron has no reference to physical conditions
				F	Out	Criteron has no reference to financial conditions
6.1.4 Freedom of association	Workers have the right to form or join organizations, in accordance to national laws, to defend their rights	S		H	Out	Criteron has no reference to human conditions
				S	In	
				N	Out	Criteron has no reference to natural conditions
				P	Out	Criteron has no reference to physical conditions
				F	Out	Criteron has no reference to financial conditions
6.1.5 Non-discrimination	• Workers do not suffer any discrimination from the employer or other workers	H	Management	H	In	

	• Written anti-discrimination regulation is in place, stating that the company does not engage/support in discrimination in hiring, remuneration, access to training, promotion, termination or retirement based on race, caste, national origin, religion, disability, gender, sexual orientation, union membership, political affiliation, age or any other condition that may give rise to discrimination			S	Out	Criteron has no reference to social conditions
				N	Out	Criteron has no reference to natural conditions
				P	Out	Criteron has no reference to physical conditions
				F	Out	Criteron has no reference to financial conditions
6.1.6 Disciplinary actions	Disciplinary actions must not be in the form of physical abuse or deduction of pay for work already completed.	H	Management	H	In	
				S	Out	Criteron has no reference to social conditions
				N	Out	Criteron has no reference to natural conditions
				P	Out	Criteron has no reference to physical conditions
				F	Out	Criteron has no reference to financial conditions
6.1.7 Migrant worker registration	Foreign and national migrant farm workers shall be legally employed with an arrangement in their language that clearly shows workers obligations to employer and vice versa.	S	Management	H	In	
				S	In	Translation of arrangement into local language
				N	Out	Criteron has no reference to natural conditions
				P	Out	Criteron has no reference to physical conditions
				F	Out	Criteron has no reference to financial conditions
6.1.8 Grievance mechanism	• All issues raised by workers must be registered, tracked and responded to by the employer.	H	Management	H	In	
				S	Out	Criteron has no reference to social conditions

	• Register is available recording issues raised by workers (including complaint forms), date and response taken. Interviews with employees confirm compliance<br>• Evidence of outside grievance mechanisms are available to workers.			N<br>P<br>F	Out<br>Out<br>Out	Criteron has no reference to natural conditions<br>Criteron has no reference to physical conditions<br>Criteron has no reference to financial conditions
6.1.9 Wage	• The farm must demonstrate lawful payment that complies with the agreed upon agreement is in place. Salary payment receipts and interviews with the workers confirm compliance.<br>• This must be reflected in the workers agreement.	H	Management	H<br>S<br>N<br>P<br>F	In<br>Out<br>Out<br>Out<br>In	 Criteron has no reference to social conditions<br>Criteron has no reference to natural conditions<br>Criteron has no reference to physical conditions<br>Ability to provide lawful payment
6.1.10 Extra work	Employees confirm that extra work is voluntary and paid in compliance with the law.	H	Management	H<br>S<br>N<br>P<br>F	In<br>Out<br>Out<br>Out<br>In	 Criteron has no reference to social conditions<br>Criteron has no reference to natural conditions<br>Criteron has no reference to physical conditions<br>Ability to provide lawful payment
**6.2 Health and Safety**						
6.2.1 Safe working conditions	• Employees are adequately protected against hazards at work (i.e. accidents caused by electrical devices).<br>• Records of all accidents and corrective action taken are available. Evidence that	H	Management	H<br>S<br>N	In<br>Out<br>Out	 Criteron has no reference to social conditions<br>Criteron has no reference to natural conditions

	corrective actions, such as invoices of medicines, are still in place.<br>• Safety equipment must be provided to workers engaged in hazardous activities.	P	Equipments needed to support activities	P	In	
				F	In	Buying safety equipment
6.2.2 Sanitary facilities	Workers have access to clean food storage areas, designated rest areas, hand washing facilities, and potable/ safe drinking water; sanitary conditions for disposal of human waste are also in place.	P	Infrastructure	H	Out	Criteron has no reference to human conditions
				S	Out	Criteron has no reference to social conditions
				N	Out	Criteron has no reference to natural conditions
				P	In	
				F	In	Building proper facility
6.2.3 Safety training	General training on safe working practice, accident prevention, risk reduction and safety must be provided to all shrimp farm workers	H	Training	H	In	
				S	Out	Criteron has no reference to social conditions
				N	Out	Criteron has no reference to natural conditions
				P	Out	Criteron has no reference to physical conditions
				F	Out	Criteron has no reference to financial conditions
6.2.4 Worker accommodation	Employee housing is constructed of materials to sustain local conditions, and separate female accommodation if required.	P	Infrastructure	H	Out	Criteron has no reference to human conditions
				S	Out	Criteron has no reference to social conditions
				N	Out	Criteron has no reference to natural conditions
				P	In	
				F	In	Building employee housing

**6.3 Community Issues**						
6.3.1 Community benefits	• Shrimp farming must demonstrate social responsibility for the benefits to the local community<br><br>• Priority should be given to hire workers from the local community	S		H<br>S<br>N<br>P<br>F	In<br>In<br>Out<br>Out<br>Out	Management in hiring<br><br>Criteron has no reference to natural conditions<br>Criteron has no reference to physical conditions<br>Criteron has no reference to financial conditions
6.3.2 Management of conflicts with local communities	• Shrimp farms should not create restriction on access to public resources and negative impacts on the local community.<br>• Shrimp farming should have mechanisms for communication and engagement with the local community and take positive actions to respond to complaints	P<br><br>S	Infrastructure<br><br>Social network with local comunity	H<br>S<br>N<br>P<br>F	Out<br>In<br>Out<br>In<br>In	Criteron has no reference to human conditions<br><br>Criteron has no reference to natural conditions<br><br>Ability to buy new farm to relocate, building proper access to the farms
6.3.3 Farm siting in the local community	• Farm site shall not obstruct the customary access and/or interfere with the living condition and activities of the local community.	P	Infrastructure	H<br>S<br>N<br>P<br>F	In<br>In<br>Out<br>In<br>In	Management of farm layout<br>Communication with local comunity<br>Criteron has no reference to natural conditions<br><br>Ability to buy new farm to relocate, building proper access to the farms
7. **Use of Species**						
7.1 Health status of post larvae	Must be in compliance with any existing national standards	H	Knowledge about national standards	H<br>S	In<br>Out	Knowledge about national standards<br>Criteron has no reference to social conditions

				N	In	Health status of post larvae
				P	Out	Criterion has no reference to physical conditions
				F	Out	Criteron has no reference to financial conditions
7.2a Sourcing of broodstock from wild sources (L.vannamei)	Prohibited	N	broodstock	H	In	Knowledge about standards
				S	Out	Criterion has no reference to social conditions
				N	In	
				P	Out	Criterion has no reference to physical conditions
				F	In	Ability to buy broodstock from other sources
7.2b Sourcing of broodstock from wild sources (P. monodon)	Records kept for sourcing of broodstock, including at a minimum: number, location, date, and method of collection	H	Recording	H	In	
				S	Out	Criterion has no reference to social conditions
				N	Out	Criterion refers to natural conditions but focus is on recording data
				P	Out	Criterion has no reference to physical conditions
				F	In	Hiring assistant to record
7.3a Use of non-native species	Non-native species shall not be used for production unless already established for commercial production and approved by the national government.	H	Knowledge	H	Out	Criterion has no reference to humanconditions
				S	Out	Criterion has no reference to social conditions
				N	In	Availability of proper species
				P	Out	Criterion has no reference to physical conditions
				F	In	Ability to buy species that are already commercial established

7.3b Use of native species	All pond stock must be spawned directly by wild- caught broodstock (i.e. F1 progeny from F0 wild-captured parents)	N		H	Out	Criterion has no reference to humanconditions
				S	Out	Criterion has no reference to social conditions
				N	In	
				P	Out	Criterion has no reference to physical conditions
				F	Out	Criteron has no reference to financial conditions
7.4 Movement of broodstock and post larvae within the country of production	• The seed supplier must provide a movement document/receipt to the farm/broker.<br>• Movement of post larvae and broodstock must be in compliance with national and/or regional laws, if applicable.	H	Documentation, knowledge	H	In	
				S	In	Supplier can provide the documents
				N	Out	Criterion refers to natural conditions but focus is on compliance with law
				P	Out	Criterion has no reference to physical conditions
				F	Out	Criterion has no reference to financial conditions
7.5 Data recording and availability	Data relevant to compliance criteria in this section are collected using robust methods and are available for inspection. Collected data must be available for inspection by auditors and/ or ASIC upon request.	H	Documentation	H	In	
				S	Out	Criterion has no reference to social conditions
				N	Out	Criterion has no reference to natural conditions
				P	Out	Criterion has no reference to physical conditions
				F	Out	Criterion has no reference to financial conditions
8. **Feed Ingredients Sourcing**						
8.1 Sources of wild fish used as feed	Wild fish sources, including by-products, used as fish meal and fish oil must be identified by species and must not be illegal.	H	Knowledge on regulations	H	In	
				S	Out	Criterion has no reference to social conditions

				N	In	Availability of proper fish sources
				P	Out	Criterion has no reference to physical conditions
				F	In	Ability to buy proper and legal fish sources
8.2 Percent Inclusion of Fishmeal	Shall be less than 20%, or 25% if fisheries by products account for at 20% of the fishmeal used in the feed formula.	H	Knowledge on percentage of ingredients	H	In	Knowledge on feed ingredients
				S	Out	Criterion has no reference to social conditions
				N	In	Percentage of ingredients
				P	Out	Criterion has no reference to physical conditions
				F	In	Ability to buy feeds with proper percentage of ingredients
8.3 Percent inclusion of fish oil	Shall be less than 4%.	H	Knowledge on percentage of ingredients	H	In	Knowledge on feed ingredients
				S	Out	Criterion has no reference to social conditions
				N	In	Percentage of ingredients
				P	Out	Criterion has no reference to physical conditions
				F	In	Ability to buy feeds with proper percentage of ingredients
8.4 Maximum protein in the feed	Shall be less than 40%.	H	Knowledge on percentage of ingredients	H	In	Knowledge on feed ingredients
				S	Out	Criterion has no reference to social conditions
				N	Out	Criterion has no reference to natural conditions
				P	In	percentage of ingredients
				F	In	Ability to buy feeds with proper percentage of ingredients
8.5 Movement of feed products	• The feed supplier must provide a movement document/receipt to the farm/broker.	H	Documentation	H	In	

within the country of production	• Movement of feed must be in compliance with national and/or regional laws, if applicable.<br>• The name and contact information for all feed suppliers used by the farm must be available.			S	In	Supplier can provide the documents
				N	Out	Criterion has no reference to natural conditions
				P	Out	Criterion has no reference to physical conditions
				F	Out	Criterion has no reference to financial conditions
8.6 Data recording and availability	Data relevant to compliance criteria in this section are collected using robust methods and are available for inspection. Collected data must be available for inspection by auditors and/ or ASIC upon request.	H	Documentation	H	In	
				S	In	Supplier can provide the documents
				N	Out	Criteron has no reference to natural conditions
				P	Out	Criteron has no reference to physical conditions
				F	Out	Criteron has no reference to financial conditions
