# Supplementary material for: A capability approach to assess aquaculture sustainability standard compliance
Source: PLoS One. 2020 Jan 23;15(1):e0227812. doi: 10.1371/journal.pone.0227812 (PMC6977730; doi:10.1371/journal.pone.0227812)
Supplement: S4 Table — (PDF) [file pone.0227812.s004.pdf]

S4 Table. Assessment of prescribed capitals and bundles of capitals for the Thai Agricultural Standard Good Aquaculture Practices for Marine Shrimp Farm.

TAS 7401					
**Requirements**	**Prescribed capitals**	**Justification**	**Bundle of capitals**	**In/out**	**Justification (Bundle of capitals)**
**1. Farm site and registration**					
1.1 Farm shall not be located in the environment that has a risk of contamination which affects shrimp health and safety of consumer or availability of contamination preventive measures.	N		H	Out	Criteron has no reference to human conditions
			S	Out	Criteron has no reference to social conditions
			N	In	
			P	Out	Criteron has no reference to physical conditions
			F	In	Financial capital to relocate farm to new location
1.2 Farm shall be located outside mangrove and/or conserved wetland areas regarding to laws.	N		H	Out	Criteron has no reference to human conditions
			S	Out	Criteron has no reference to social conditions
			N	In	
			P	Out	Criteron has no reference to physical conditions
			F	In	Financial capital to relocate farm to new location
1.3 Closely located to quality water suitable for shrimp culture.	N		H	Out	Criteron has no reference to human conditions
			S	Out	Criteron has no reference to social conditions
			N	In	
			P	In	Infrastructure and accessibility to qaulity water, from other sources
			F	In	Financial capital to relocate farm to new location
1.4 Conveniently access to transportation both outside and inside the farm, in order to provide a convenient operation and rapid transportation of shrimp.	P	Infrastructure	H	Out	Criteron has no reference to human conditions
			S	Out	Criteron has no reference to social conditions
			N	Out	Criteron has no reference to natural conditions
			P	In	
			F	In	Financial capital to relocate farm to new location
	N		H	In	Management

1.5 Farm shall be registered with the Department of Fisheries as well as legally land rights or other land use permits.		Ownership rights of land	S	Out	Criteron has no reference to social conditions
			N	In	
			P	Out	Criteron has no reference to physical conditions
			F	Out	Criteron has no reference to financial conditions
**2. Farm management**					
2.1 Manual of Farm Management shall be made available and implemented.	H		H	In	
			S	In	Learning from neighbour's manual as example.
			N	Out	Criteron has no reference to natural conditions
			P	Out	Criteron has no reference to physical conditions
			F	In	Hiring assistant to create manual
2.2 Water testing from sources according to the specified time intervals in the manual	H	Testing	H	In	
			S	Out	Criteron has no reference to social conditions
			N	Out	Criteron has no reference to natural conditions
			P	Out	Criteron has no reference to physical conditions
			F	In	Hiring assistant to test and record
2.3 Vacating and/or preparing pond between crops.	H		H	In	
			S	Out	Criteron has no reference to social conditions
			N	In	Available land for vacating and preparing pond
			P	In	Equipment for preparing pond
			F	In	Paid assistance
2.4 Stocking density of shrimp larvae shall be appropriate.	N		H	Out	Criteron has no reference to human conditions
			S	Out	Criteron has no reference to social conditions
			N	In	
			P	Out	Criteron has no reference to physical conditions
			F	Out	Criteron has no reference to financial conditions
2.5 Availability of record/ certificate/ health test report.	H		H	In	
			S	In	Test report from DoF, feed company
			N	Out	Criteron has no reference to natural conditions
			P	Out	Criteron has no reference to physical conditions
			F	Out	Criteron has no reference to financial conditions

2.6 Inlet water shall be infiltrated to prevent the entering of exotic species to pond.	P	Barrier	H	Out	Criteron has no reference to human conditions
			S	Out	Criteron has no reference to social conditions
			N	Out	Criteron has no reference to natural conditions
			P	In	
			F	In	Building barrier, paid labour
2.7 Aerator or other aeration system shall be adequately placed in the pond.	P	Infrastructure	H	In	Knowledge, skills in installing aeration system
			S	Out	Criteron has no reference to social conditions
			N	Out	Criteron has no reference to natural conditions
			P	In	
			F	In	Ability to buy aerator
2.8 Use of registered, good quality and not expired formulated feed.	H	Management	H	In	
			S	In	Connection with feed companies
			N	Out	Criteron has no reference to natural conditions
			P	Out	Criteron has no reference to physical conditions
			F	In	Financial capital in buying certified feeds
2.9 In case the feed is prepared on the farm, feed ingredients shall be clearly stated, and legally prohibited ingredients shall not be used.	H	Management	H	In	
			S	Out	Criteron has no reference to social conditions
			N	Out	Criteron has no reference to natural conditions
			P	In	Availability proper ingredients
			F	Out	Criteron has no reference to financial conditions
2.10 Efficient feeding management shall be provided according to the requirements of shrimp.	H	Management	H	In	
			S	Out	Criteron has no reference to social conditions
			N	Out	Criteron has no reference to natural conditions
			P	In	Infrastructure, system, equipment
			F	Out	Criteron has no reference to financial conditions
2.11 Feed shall be stored in secured place to prevent the contamination and maintain its quality.	P	Storage	H	Out	Criteron has no reference to human conditions
			S	Out	Criteron has no reference to social conditions
			N	Out	Criteron has no reference to natural conditions
			P	In	
			F	In	Building of storage

2.12 Analysis of water quality in shrimp pond on a regular basis.	H	Monitoring, analysis	H	In	
			S	Out	Criteron has no reference to social conditions
			N	Out	Criteron refers to natural conditions but focus is on testing
			P	Out	Criteron has no reference to physical conditions
			F	In	Hiring assistant to test and record
2.13 Preventive measures for predators and disease carriers to enter the ponds during pond, water preparation and shrimp culturing shall be in place.	P	Infrastructure, barriers	H	In	Management
			S	Out	Criteron has no reference to social conditions
			N	Out	Criteron has no reference to natural conditions
			P	In	
			F	In	Buidling of barriers
2.14 Shrimp health shall be monitored regularly.	H	Monitoring	H	In	
			S	Out	Criteron has no reference to social conditions
			N	Out	Criteron refers to natural conditions but focus is on monitoring
			P	Out	Criteron has no reference to physical conditions
			F	In	Hiring assistant to monitor
2.15 Shrimp health management programme should be implemented in compliance with relevant national legislations taking into account the FAO CCRF Technical Guidelines on Health Management for Responsible Movement of Live Aquatic Animals and relevant OIE Standards.	H	Management	H	In	
			S	Out	Criteron has no reference to social conditions
			N	Out	Criteron has no reference to natural conditions
			P	Out	Criteron has no reference to physical conditions
			F	Out	Criteron has no reference to financial conditions
2.16 Movement of live shrimp and shrimp product should take place in accordance with the relevant provisions in the OIE Aquatic Animal Health Code to prevent introduction or transfer of disease and	H	Management	H	In	
			S	Out	Criteron has no reference to social conditions
			N	Out	Criteron has no reference to natural conditions
			P	Out	Criteron has no reference to physical conditions
			F	Out	Criteron has no reference to financial conditions

infectious nts pathogenic to shrimp while avoiding unwarranted sanitary measures.					
2.17 In case of disease outbreak, farmer shall inform the competent authority immediately.	H	Management	H	In	
			S	In	Social networks with authorities
			N	Out	Criteron has no reference to natural conditions
			P	Out	Criteron has no reference to physical conditions
			F	Out	Criteron has no reference to financial conditions
2.18 In case where shrimp shows any sign of poor health and/or symptom, diagnosis, causation analysis and corrective actions shall be carried out.	H	Management	H	In	
			S	In	Learning and discuss with neighbours, other farmers
			N	Out	Criteron has no reference to natural conditions
			P	Out	Criteron has no reference to physical conditions
			F	Out	Criteron has no reference to financial conditions
2.19 Preventive measure and control of disease outbreak shall be in place.	P	Equipment	H	In	Management
			S	In	Learning and discuss with neighbours, other farmers
			N	Out	Criteron has no reference to natural conditions
			P	In	
			F	In	Ability to buy and install proper preventive system
2.20 Where polyculture is conducted, effective measures should be taken to reduce potential disease transmission between culture species.	H	Management	H	In	
			S	In	Learning and discuss with neighbours, other farmers
			N	In	Ability to reduce potential disease transmission
			P	In	Equipment
			F	Out	Criteron has no reference to financial conditions
2.21 Worker should be trained on good aquatic animal health and welfare management practices to ensure they are aware of their roles and responsibilities in maintaining shrimp health and welfare.	H	Training	H	In	
			S	Out	Criteron has no reference to social conditions
			N	Out	Criteron has no reference to natural conditions
			P	Out	Criteron has no reference to physical conditions
			F	Out	Criteron has no reference to financial conditions
**3. Use of veterinary drugs, chemicals, and probiotics used in aquaculture**					
3.1 Veterinary drugs, chemicals and probiotics as prohibited by law shall not be used. Veterinary drugs, chemicals and	H	Management	H	In	
			S	Out	Criteron has no reference to social conditions
			N	Out	Criteron has no reference to natural conditions

probiotics used in aquaculture shall be registered with the competent authority and prudently used.			P	In	Other proper drugs, chemicals
			F	In	Ability to buy drugs, chemicals that are not prohibits
3.2 In case authorized veterinary drugs or chemicals are applied prior to harvesting, withdrawal period shall be strictly followed or used according to the label instruction.	P		H	In	Management
			S	Out	Criteron has no reference to social conditions
			N	Out	Criteron has no reference to natural conditions
			P	In	
			F	Out	Criteron has no reference to financial conditions
3.3 Veterinary drugs, chemicals and probiotics shall be appropriately stored to prevent deterioration and danger.	P	Storage	H	Out	Criteron has no reference to human conditions
			S	Out	Criteron has no reference to social conditions
			N	Out	Criteron has no reference to natural conditions
			P	In	
			F	In	Ability to build proper storage
**4. Effluent and Sediment management**					
4.1 Quality of effluent shall be complied with relevant laws and regulations.	H	Knowledge	H	In	
			S	Out	Criteron has no reference to social conditions
			N	Out	Criteron has no reference to natural conditions
			P	Out	Criteron has no reference to physical conditions
			F	Out	Criteron has no reference to financial conditions
4.2 Preventing of saline water discharged into freshwater area shall be in place for environmental protection.	P	Infrastructure, barrier	H	In	Management
			S	Out	Criteron has no reference to social conditions
			N	Out	Criteron has no reference to natural conditions
			P	In	
			F	In	Ability to build barrier
4.3 Sediment shall not be disposed into public or non-permitted area.	H	Management	H	In	Knowledge
			S	In	Social norm
			N	Out	Criteron has no reference to natural conditions
			P	In	Proper equipment or container
			F	In	Building or install proper container
**5. Energy source and fuel**					

5.1 Fuel and lubricant shall be stored properly and securely.	P	Storage	H	In	Management
			S	Out	Criteron has no reference to social conditions
			N	Out	Criteron has no reference to natural conditions
			P	In	
			F	In	Ability to build proper storage
5.2 Machine used on farm shall be in good condition without any fuel or lubricant leakage to water source.	P	Equipment	H	In	Management and skills in maintanance
			S	Out	Criteron has no reference to social conditions
			N	Out	Criteron has no reference to natural conditions
			P	In	
			F	In	Ability to buyequipment and maintanance
5.3 Used lubricant shall be disposed of in container and properly eliminated.	P	Container	H	Out	Criteron has no reference to human conditions
			S	Out	Criteron has no reference to social conditions
			N	Out	Criteron has no reference to natural conditions
			P	In	
			F	In	Ability to build or buy proper container
5.4 There shall be safe electricity system on farm.	P	Infrastructure	H	Out	Criteron has no reference to human conditions
			S	Out	Criteron has no reference to social conditions
			N	Out	Criteron has no reference to natural conditions
			P	In	
			F	In	Ability to install proper system
5.5 Save use of energy and/or renewable energy sources.	H	Management	H	In	
			S	Out	Criteron has no reference to social conditions
			N	Out	Criteron has no reference to natural conditions
			P	In	Energy
			F	Out	Criteron has no reference to financial conditions
**6. Farm sanitation**					
6.1 Garbage, refuse, veterinary drug and chemical containers shall be separately managed to prevent contamination to shrimp.	P	Infrastructure	H	In	Management
			S	Out	Criteron has no reference to social conditions
			N	Out	Criteron has no reference to natural conditions
			P	In	

			F	In	Ability to build proper storage
6.2 Keep in order the production inputs, materials and equipment so as not to harbour other animals/pest .	P		H	In	Management
			S	Out	Criteron has no reference to social conditions
			N	Out	Criteron has no reference to natural conditions
			P	In	
			F	In	Ability to build proper storage
6.3 Bathroom and toilet shall be hygienicly designed to prevent contamination to culture pond, canal and/or water sources.	P	Infrastructure	H	Out	Criteron has no reference to human conditions
			S	Out	Criteron has no reference to social conditions
			N	Out	Criteron has no reference to natural conditions
			P	In	
			F	In	Ability to build proper toilet
6.4 Manure shall not be used.	H	Management	H	In	
			S	Out	Criteron has no reference to social conditions
			N	Out	Criteron has no reference to natural conditions
			P	Out	Criteron has no reference to physical conditions
			F	Out	Criteron has no reference to financial conditions
6.5 Pets are not allowed in the production area.	P	Proper area for pet	H	In	Management
			S	Out	Criteron has no reference to social conditions
			N	Out	Criteron has no reference to financial conditions
			P	In	
			F	Out	Criteron has no reference to financial conditions
6.6 Worker should be trained in good hygienic practices to ensure they are aware of their roles and responsibilities for protecting shrimp from contamination and deterioration.	H	Training	H	In	
			S	Out	Criteron has no reference to social conditions
			N	Out	Criteron has no reference to financial conditions
			P	Out	Criteron has no reference to physical conditions
			F	Out	Criteron has no reference to financial conditions
**7. Harvest and post harvest handlings prior to distribution**					
	H	Management	H	In	Knowledge on regulation

7.1 Prohibited chemicals shall not be used during harvesting as well as post harvest.			S	Out	Criteron has no reference to social conditions
			N	Out	Criteron has no reference to financial conditions
			P	In	Proper chemicals
			F	In	Ability to buy proper approved chemicals
7.2 If chemicals are used, it shall be properly used in terms of type and quantity.	H	Management	H	In	
			S	Out	Criteron has no reference to social conditions
			N	Out	Criteron has no reference to natural conditions
			P	In	Proper chemical
			F	Out	Criteron has no reference to financial conditions
7.3 Select buyer/collector that has been certified in good hygienic practices of the post-harvest handling and transportation or registered with the Department of Fisheries	S	Social networks with certified buyers	H	In	Management
			S	In	
			N	Out	Criteron has no reference to natural conditions
			P	Out	Criteron has no reference to physical conditions
			F	Out	Criteron has no reference to financial conditions
7.4 Availability of good hygienic practices on harvesting to prevent contamination	H	Knowledge	H	In	
			S	In	Learning example from neighbour, other farmers, experts
			N	Out	Criteron has no reference to natural conditions
			P	Out	Criteron has no reference to physical conditions
			F	Out	Criteron has no reference to financial conditions
**8. Labour and welfare**					
8.1 Farm workers shall be legally employed.	H	Management	H	In	
			S	Out	Criteron has no reference to social conditions
			N	Out	Criteron has no reference to natural conditions
			P	Out	Criteron has no reference to physical conditions
			F	Out	Criteron has no reference to financial conditions
8.2 Wages shall be abided by law.	H	Management	H	In	
			S	Out	Criteron has no reference to social conditions
			N	Out	Criteron has no reference to natural conditions
			P	Out	Criteron has no reference to physical conditions
			F	Out	Criteron has no reference to financial conditions

8.3 Welfare for workers shall be appropriately provided.	H	Management	H	In	
			S	Out	Criteron has no reference to social conditions
			N	Out	Criteron has no reference to natural conditions
			P	Out	Criteron has no reference to physical conditions
			F	Out	Criteron has no reference to financial conditions
8.4 Provide precautions and working equipment for safe operation.	P	Equipment	H	In	
			S	Out	Criteron has no reference to social conditions
			N	Out	Criteron has no reference to natural conditions
	H	Management	P	In	
			F	In	Ability to buy safe and working equipments
8.5 Workers shall be trained on safety of operation.	H	Training	H	In	
			S	Out	Criteron has no reference to social conditions
			N	Out	Criteron has no reference to natural conditions
			P	Out	Criteron has no reference to physical conditions
			F	Out	Criteron has no reference to financial conditions
**9. Social and environmental responsibilities**					
9.1 Farm site shall not obstruct the customary access and/or interfere with the living condition and activities of the local community.	P	Infrastructure	H	Out	Criteron has no reference to human conditions
			S	In	Relations and communications with local community
			N	In	Farm location
			P	In	
			F	In	Ability to relcate the farm
9.2 Priority should be given to hire workers from the local community.	S		H	In	Management
			S	In	
			N	Out	Criteron has no reference to natural conditions
			P	Out	Criteron has no reference to physical conditions
			F	Out	Criteron has no reference to financial conditions
9.3 Benefits to the local community should be considered at all stages of shrimp farming.	S		H	In	Management
			S	In	
			N	Out	Criteron has no reference to natural conditions

			P	Out	Criteron has no reference to physical conditions
			F	Out	Criteron has no reference to financial conditions
9.4 Having good relationship with local community.	S		H	In	Management
			S	In	
			N	Out	Criteron has no reference to natural conditions
			P	Out	Criteron has no reference to physical conditions
			F	Out	Criteron has no reference to financial conditions
9.5 Having mechanisms for communication and engagement with the local community and take positive actions to respond to complaints.	S		H	In	Management
			S	In	
			N	Out	Criteron has no reference to natural conditions
			P	Out	Criteron has no reference to physical conditions
			F	Out	Criteron has no reference to financial conditions
9.6 Join and participate in shrimp farm organizations or other related professional organizations.	S	Participate in social networks	H	Out	Criteron has no reference to human conditions
			S	In	
			N	Out	Criteron has no reference to natural conditions
			P	Out	Criteron has no reference to physical conditions
			F	Out	Criteron has no reference to financial conditions
9.7 Use of hatchery seed preferably, natural seed should be avoided.	H	Management	H	In	
			S	Out	Criteron has no reference to social conditions
			N	In	Availiability of hatchery seed
			P	Out	Criteron has no reference to physical conditions
			F	In	Ability to buy proper hatchery seed
9.8 Use of exotic species only after positive assessment of Import Risk Analysis followed by appropriate quarantine measures.	N		H	In	Management
			S	Out	Criteron has no reference to social conditions
	F	Paid experts for conducting assessment	N	In	
			P	Out	Criteron has no reference to physical conditions
			F	In	
9.9 Shrimp whose genetic material has been altered in a way that does not occur naturally shall not be use.	H	Management	H	In	
			S	Out	Criteron has no reference to social conditions
			N	In	Ability to find/avoid certain shrimps

			P	Out	Criteron has no reference to physical conditions
			F	In	Ability to buy proper shrimps
9.10 Appropriate measures to minimize escape of shrimp should be in place.	P	Barrier	H	In	Management
			S	Out	Criteron has no reference to social conditions
			N	Out	Criteron has no reference to natural conditions
			P	In	
			F	In	Ability to build barrier
9.11 Damage caused by previous shrimp farming operation should be rehabilitated on or close to the farm site.	N		H	In	Rehabilitation
			S	Out	Criteron has no reference to social conditions
			N	In	
			P	Out	Criteron has no reference with physical conditions
			F	In	Ability to relocate the farm, financial capitals for rehabilitation
9.12 Effective mitigation measures should be taken if the current practices are effected to habitat/environment such as leakaging pond.	H	Management	H	In	
			S	Out	Criteron has no reference to social conditions
			N	Out	Criteron has no reference to natural conditions
			P	In	Barrier
			F	Out	Criteron has no reference to financial conditions
9.13 On-farm and off-farm environmental quality indicators should be monitored routinely including with the community participation.	H	Monitoring	H	In	
			S	Out	Criteron has no reference to social conditions
			N	Out	Criteron has no reference to natural conditions
			P	Out	Criteron has no reference to physical conditions
			F	In	Hiring assistant to monitor
9.14 Participate in conference, seminar, or training on issues related to environmentally friendly shrimp culture, shrimp health and animal welfare and food safety.	S		H	Out	Criteron has no reference to human conditions
			S	In	
			N	Out	Criteron has no reference to natural conditions
			P	Out	Criteron has no reference to physical conditions
			F	Out	Criteron has no reference to financial conditions
**10. Document and record keeping**					
	H	Documentation	H	In	

10.1 Fry movement document (FMD) and Movement Document (MD) shall be present upon request.			S	In	Supplier and buyers can provide documents
			N	Out	Criteron has no reference with natural conditions
			P	Out	Criteron has no reference with physical conditions
			F	Out	Criteron has no reference with financial conditions
10.2 Use of veterinary drugs, chemicals, hazardous substances and probiotics shall be records.	H	Documentation	H	In	
			S	Out	Criteron has no reference to social conditions
			N	Out	Criteron has no reference to natural conditions
			P	Out	Criteron has no reference to physical conditions
			F	In	Hiring assistant to record
10.3 Use of chemicals during harvesting shall be records.	H	Documentation	H	In	
			S	Out	Criteron has no reference to social conditions
			N	Out	Criteron has no reference to natural conditions
			P	Out	Criteron has no reference to physical conditions
			F	In	Hiring assistant to record
10.4 Records on the shrimp culture data and other necessary information such as diseases prevention and water quality shall be kept for further inspection.	H	Documentation	H	In	
			S	Out	Criteron has no reference to social conditions
			N	Out	Criteron has no reference to natural conditions
			P	Out	Criteron has no reference to physical conditions
			F	Out	Criteron has no reference with financial conditions
