# Supplementary material for: A capability approach to assess aquaculture sustainability standard compliance
Source: PLoS One. 2020 Jan 23;15(1):e0227812. doi: 10.1371/journal.pone.0227812 (PMC6977730; doi:10.1371/journal.pone.0227812)
Supplement: S5 Table — (PDF) [file pone.0227812.s005.pdf]

S5 Table. Summary of certification support programs.

Standard	Indicators	BAP	ASC	SEASAIP	TAS 7401
**Human Capital**	Skills	• GAA (standard owner) developed an improvement programme called iBAP by collaborating with processor that sponsored the farms. The programme provides guidelines on what farmers should do to comply with standards along with analyse the gaps between their current practices and BAP standards to provide suggestions *(Direct involvement).*	• ASC is currently developing its improvement programme. It also plans to collaborate with the bank with the idea that the bank can assist in providing funding, provide loan with low interest for farmers to make necessary improvements *(Direct involvement).*<br>• WWF, a partner organization, established Market Transformation Initiative (MTI) and Aquaculture Improvement Project (AIP) to help farmers achieve ASC certification by analysing the gaps between their current practices and ASC standards then develop and execute an action plan to improve their practices [5] *(Direct involvement).*	• SEASAIP conducted a pre-assessment at the beginning when introducing SEASAIP to farms. This was put in place to analyse the gaps between their current practices or farms management and SEASAIP standards. With this information, they then can give suggestions for farmers to develop proper plans for improvement *(Direct involvement).*	• There is no formal improvement programme developed for this standard. However, both ACFS (standard owner) and Department of Fisheries (DoF) have been providing a good practice training to farmers in field visits for training, seminars or shrimp day event *(Direct involvement).*

	Knowledge	• BAP representatives in the country collaborate with the processor that sponsored the farms to provide general training and general information on what the BAP standard is, and what the requirements are *(Direct involvement)*.	• Local expert organizations or NGO such as WWF in the case of Thailand provides general information on ASC to farmers, and companies that involve in supply chain or production process to become more interested in ASC standard *(Direct involvement)*.	• SEASAIP representative provides general training and general information on SEASAIP requirements to farmers *(Direct involvement)*.	• DoF has been providing general information about the importance of this standard to farmers, directly and through broker. This is for farmers to have better understanding of the standard and how the requirements are different from previous standard (GAP) *(Direct involvement).*
	Labour management	• There is not any evidence provided that any kind of support has been given *(No support*).	• There is not any evidence provided that any kind of support has been given *(No support*).	• There is not any evidence provided that any kind of support has been given *(No support*).	• There is not any evidence provided that any kind of support has been given *(No support*).
	Farming, pound management	• An improvement programme, called iBAP provides a guidance manual on what are the good practices BAP is looking for. This guidance is so that farmers should follow regarding farming practices, pound management for farmers to have better understanding or guideline to follow *(Direct involvement)*.	• ASC provides guidance materials as guideline and example of good practices for farmers to follow. The materials are for both general topics and tailor-made for specific species. ASC also developing an idea on using online movie clips as another example for farmers to learn from *(Direct involvement)*.	• Currently, no actual guidance materials have been provided yet. But there is already an idea on this and is currently being developed *(Direct involvement).*	• ACFS created guidance manual on the application of standard that provides guidelines and example for framers to learn from on how good practices should be conducted [6] *(Direct involvement).*

	Documentation, Data collection	• BAP is currently developing an idea of using smartphones (physical capital) to facilitate in directly collecting the data from farmers to reduce the number of paper-based recording *(Indirect involvement)*.<br>• BAP also simplified its form on feeds section, which makes it less confusing and require less time for farmers in filling them out *(Direct involvement)*.	• There is not any evidence provided that any kind of support has been given *(No support*).	• SEASAIP aims to reduce the number of documents required for data collection. SEASAIP collaborates with its partner stakeholder, FairAgora Asia, to develop the data collection method through the use of physical capital in the form of technology systems to collect the data directly from farms and cooperatives and digitalise them for storing those data and for auditor to access them for evaluation [7] *(Indirect involvement)*.	• ACFS simplified the log book to require less detailed to be filled out. This is to encourage farms to spend less time to record the data *(Direct involvement)*.
	General assessment	• There is not any evidence provided that any kind of support has been given *(No support*).	• ASC plans to have one general assessment to be conducted for the area, or community. Then farmers in that area can use it (through network of social capital) as shared assessment and source of information. This is to reduce the cost for every farmers to conduct the similar assessment on the same area *(Indirect involvement)*.	• There is not any evidence provided that any kind of support has been given *(No support)*.	• There is not any evidence provided that any kind of support has been given *(No support*).

**Social Capital**	Knowledge sharing	• BAP offers group certification for farmers under formal social organization such as cooperative. This is for farmers to share their knowledge, experiences, helping each other to improve and for collective action and management *(Direct involvement)*.<br>• BAP assigned farmers who have experienced or BAP certified farms to share knowledge and suggestions with other farmers who are interested in applying for BAP compliance *(Direct involvement)*.	• ASC offers group certification for farmers that participate in professional group management for administrative functions. This is for farmers to learn and share their knowledge, skills, and help each other to improve to get certified *(Direct involvement)*.<br>• Partnership organization, such as WWF, provides transition or improvement programme to farmers who want to improve their practices toward ASC compliance *(Direct involvement)*.	• SEASAIP offers group certification for farmers under cluster farming management among cooperatives or group of commercial farms. This is for farmers to learn from each other, and help each to address their weakness and improve their practices together toward SEASAIP compliance *(Direct involvement)*.	• ACFS offers group certification for farmers in neighbouring area or cooperatives. Although few random farms will be picked for audit, group certification offers quality control for farmers to learn from each other, and help each other address issues and improving toward compliance *(Direct involvement)*.
	Connections with others	• BAP often collaborates with processor that sponsored the farm to promote BAP or provide support for farms to get certified *(Direct involvement)*.	• WWF promotes ASC to farmers (through processor) for interest on ASC and for compliance *(Direct involvement)*.<br>• Local organizations can offer assistance in term of knowledge, service or act as project manager to assist farmers to improve	• SEASAIP collaborates with business partners, buyers, NGOs, academic to promote SEASAIP and too improve its criteria to be more closer to the reality of the practices in Southeast Asian. This includes processor to provide support, or contract for farms to get	• Although ACFS is the owner of the standard, instead DoF is the agency that has the main responsibility on managing aquaculture production. DoF is the agency that promotes the standard, or answering farmer's questions regarding the

			toward ASC compliance *(Direct involvement)*.	SEASAIP certified *(Direct involvement)*.	standards *(Direct involvement)*.
	Communication with community	• BAP gives lesson or training on how to reduce the gap between their current practices and BAP's requirements through leaning less from farmers who have previous experience in getting BAP certified *(Direct involvement)*.	• There is not any evidence provided that any kind of support has been given (No support).	• Farmers communicate or learn from other farms within the cluster to learn and exchange information about their current practices or updating on news or information *(Direct involvement)*.	• Famers learn from other neighbouring farms, or cooperatives, or local shrimp clubs, or regional association in exchanging information and knowledge, including updating news or trends *(Direct involvement)*.
	Communication with authorities	• There is not any evidence provided that any kind of support has been given (No support).	• There is not any evidence provided that any kind of support has been given (No support).	• There is not any evidence provided that any kind of support has been given (No support).	• Farmers can have frequent communication, contact or interaction with local DoF officers in the region regards information on the standard or good practices *(Direct involvement)*.
	Social network, connection with suppliers	• Processor that sponsored the farms to get BAP certified may give information or work closely with suppliers that can provide proper supply, equipment to farmers *(Direct involvement).*	• Processor that sponsored the farms to get ASC certified may give information or work closely with suppliers that can provide proper supply, equipment to farmers *(Direct involvement).*	• Buyers and industry stakeholders in SEASAIP committee such as Thai Union Frozen, Chicken of the Sea, Socksargen Federation of Fishing & Allied Industries can provide connection to supplier that have proper equipment or	• Broker or supplier provides information and connection to farmers on how and where they can buy certified feeds. Since farmers are usually in close contact with broker they can receive information about feeds, and information

				supply needed for improvement or certified equipment that is part of SEASAIP criteria *(Direct involvement)*.	about standard itself from brokers *(Direct involvement)*.
	Participate in social organization, collective representation	• For farmers to participate in group certification, they should participate or be parted of formal social organization such as cooperative. This is for them to collectively help each other to address the issues they may have and improve toward BAP compliance *(Direct involvement)*.	• There is not any evidence provided that any kind of support has been given *(No support)*.	• SEASAIP encourages farmers to participate in cluster farm, through cooperatives. This is for farmers to collectively help each other to address the issues they may have and improve toward SEASAIP compliance *(Direct involvement)*.	• Thai farmers usually participate in cooperatives, local shrimp clubs, or (national, regional or local) shrimp association to learn and share knowledge and information among each other. They also discuss their opinions about TAS 7401, and have the opinions have to association representatives, who then can discuss this further with DoF or ACFS representatives *(Direct involvement)*.
**Natural Capital**	Farm location	• There is not any evidence provided that any kind of support has been given *(No support)*.	• There is not any evidence provided that any kind of support has been given *(No support)*.	• There is not any evidence provided that any kind of support has been given *(No support)*.	• There is not any evidence provided that any kind of support has been given *(No support)*.
	Natural barriers	• There is not any evidence provided that any kind of support has been given *(No support)*.	• There is not any evidence provided that any kind of support has been given *(No support)*.	• There is not any evidence provided that any kind of support has been given *(No support)*.	• There is not any evidence provided that any kind of support has been given *(No support)*.

	Water, soil quality	• There is not any evidence provided that any kind of support has been given *(No support)*.	• There is not any evidence provided that any kind of support has been given *(No support)*.	• There is not any evidence provided that any kind of support has been given *(No support)*.	• There is not any evidence provided that any kind of support has been given *(No support)*.
	Specific shrimp larvae species	• There is not any evidence provided that any kind of support has been given *(No support)*.	• There is not any evidence provided that any kind of support has been given *(No support)*.	• There is not any evidence provided that any kind of support has been given *(No support)*.	• There is not any evidence provided that any kind of support has been given *(No support)*.
	Restoring the area	• There is not any evidence provided that any kind of support has been given *(No support)*.	• There is not any evidence provided that any kind of support has been given *(No support)*.	• There is not any evidence provided that any kind of support has been given *(No support)*.	• There is not any evidence provided that any kind of support has been given *(No support)*.
**Physical Capital**	Infrastructure	• IDH, a partner organization, developed Farmers in Transition Fund to provide funding for farmers to make necessary changes that are identified through assessment from iBAP programme. This includes farm renovation to comply with BAP *(Indirect involvement)*.	• ASC is developing an idea to provide funding farmers to make substantial infrastructure improvement. This includes the cooperation with the bank in providing loan with low interests *(Indirect involvement)*.	• SEASAIP fosters the partnership with their stakeholders, which include market actors in supply chain. SEASAIP aims to establish commitment with those buyers, companies to ensure there will be price premium or buying commitment from them to provide funding for farmers in making necessary infrastructure improvement *(Indirect involvement)*.	• Thai Government has developed temporary policy in 2016 to subside loan interests for farmers. This is for farmers to have the opportunities to improve, renovate, or reconstruct their farm's infrastructure to comply with standard at low cost *(Indirect involvement)*.
	Approved equipment,	• IDH, a partner organization, developed Farmers in Transition	• There is not any evidence provided that any kind of support has	• SEASAIP fosters the partnership with their stakeholders, which	• Thai Government has developed several policies and projects

	devices, materials	Fund to provide funding for farmers to make necessary changes that are identified through assessment from iBAP programme. This includes buying or sharing devices, martials, and equipment *(Indirect involvement)*.	been given *(No support)*.	include market actors in supply chain. SEASAIP aims to establish commitment with those buyers, companies to ensure there will be price premium or buying commitment from them to provide funding for farmers in making necessary improvement through buying or sharing devices, martials, and equipment *(Indirect involvement)*.	that collaborates with suppliers to reduce prices of devices. This is to assist or support farmers to buy necessary devices, materials for renovating or improving their farms at low price *(Indirect involvement)*.
	Container, storage	• There is not any evidence provided that any kind of support has been given *(No support)*.	• There is not any evidence provided that any kind of support has been given *(No support)*.	• There is not any evidence provided that any kind of support has been given *(No support)*.	• There is not any evidence provided that any kind of support has been given *(No support)*.
	Approved probiotics	• There is not any evidence provided that any kind of support has been given *(No support)*.	• There is not any evidence provided that any kind of support has been given *(No support)*.	• There is not any evidence provided that any kind of support has been given *(No support)*.	• DoF gives Bacillus spp. in probiotic (Pormor-1 product) to farmers for free to improve the environmental aspect of water quality and overall production [8] *(Direct involvement)*.
	Specific feeds	• Farmers receive information about certified feeds and which suppliers can provide those feeds through the connection	• Farmers receive information about certified feeds and which suppliers can provide those feeds through the connection	• Farmers receive information about certified feeds and which suppliers can provide those feeds through the connection	• Farmers receive information about certified feeds and which suppliers can provide those feeds through the connection

		or social network with processor that they have contract or working with *(Indirect involvement)*.<br>• Farmers also receive information and collectively negotiate as cooperative with suppliers in buying proper feeds *(Indirect involvement)*.	or social network with processor that they have contract or working with *(Indirect involvement)*.	or social network with processor that they have contract or working with *(Indirect involvement)*.<br>• Farmers also receives information about certified feeds and where they can buy them from social network of SEASAIP stakeholders, especially companies, or processors *(Indirect involvement)*.	or social network with processor, brokers that they have contract or working with *(Indirect involvement)*.<br>• Farmers also receives information about certified feeds and where they can buy them from cooperatives *(Indirect involvement)*.<br>• Thai Government has developed several policies and projects that collaborates with suppliers to reduce prices of feeds for farmers to acquire them at lower cost *(Indirect involvement)*.
	Irrigation, feeding system	• There is not any evidence provided that any kind of support has been given *(No support)*.	• There is not any evidence provided that any kind of support has been given *(No support)*.	• There is not any evidence provided that any kind of support has been given *(No support)*.	• There is not any evidence provided that any kind of support has been given *(No support)*.
**Financial Capital**	Purchasing certified, specific feeds	• In case of farmers that participate in group certification, farmers receive information through social network and collectively negotiate as cooperative with suppliers in buying proper feeds at lower price than individual	• There is not any evidence provided that any kind of support has been given *(No support)*.	• In case of farmers that participate in group certification, farmers receive information through social network and collectively negotiate as cooperative with suppliers in buying proper feeds at lower price than individual	• In case of farmers that participate in group certification, farmers receive information through social network and collectively negotiate as cooperative with suppliers in buying proper feeds at lower price than

		buying *(Indirect involvement).*		buying *(Indirect involvement).*	individual buying *(Indirect involvement).*
	Hiring assistant	• There is not any evidence provided that any kind of support has been given *(No support).*	• There is not any evidence provided that any kind of support has been given *(No support).*	• There is not any evidence provided that any kind of support has been given *(No support).*	• There is not any evidence provided that any kind of support has been given *(No support).*
	Assistance for construction	• Through social network with IDH, a partner organization, that developed the Farmers in Transition Fund to provide funding for farmers to make necessary changes that are identified through assessment from iBAP programme. This includes hiring workers for assistance to renovate the farms *(Indirect involvement).*	• Through social network of partnership with WWF or local organization that provide assistance and funding through impartment or transition program for substantial infrastructure improvement that include hiring assistance to renovate the farms *(Indirect involvement).*	• There is not any evidence provided that any kind of support has been given *(No support).*	• Thai Government has developed policies that subside loan interests or providing financial support for farmers to improve or renovate their farms. This includes hiring workers for assistance to renovate the farms *(Direct involvement).*
	Buying proper equipment, supplies	• Through social network with IDH, a partner organization, that developed the Farmers in Transition Fund to provide funding for farmers to make necessary changes that are identified through assessment from iBAP programme. This includes buying proper equipment to make	• Through social network of partnership with WWF or local organization that provide assistance and funding through impartment or transition program for substantial infrastructure improvement that include buying proper equipment to make	• There is not any evidence provided that any kind of support has been given *(No support).*	• Thai Government has developed several policies and projects that collaborates with suppliers to reduce prices of devices. This is to assist or support farmers to buy necessary devices, materials for renovating or improving their farms at lower price *(Direct involvement).*

		improvement *(Indirect involvement)*.	improvement *(Indirect involvement)*.		
	Hiring expert to conduct assessment	• There is not any evidence provided that any kind of support has been given *(No support)*.	• There is not any evidence provided that any kind of support has been given *(No support)*.	• There is not any evidence provided that any kind of support has been given *(No support)*.	• There is not any evidence provided that any kind of support has been given *(No support)*.

References

1. Global Aquaculture Alliance. Aquaculture Facility Certification Finfish and Crustacean Farms. Best Aquaculture Practices Certification Standards, Guidelines. New Hampshire, USA: Global Aquaculture Alliance (GAA); 2017.
2. ASC. ASC Shrimp Standard v1.0 - March 2014. Utrecht, The Netherlands: Aquaculture Stewardship Council (ASC); 2014.
3. SEASAIP. The Southeast Asian Shrimp Aquaculture Improvement Protocol Draft #5. Southeast Asian Shrimp Aquaculture Improvement Protocol (SEASAIP); 2016.
4. Thai Agricultural Standard TAS 7401-2014, TAS 7401-2014 (2014).
5. WWF. First ASC-Certified Shrimp Farm in Thailand is a Big Step Forward for Responsible Aquaculture 2016 [updated 31 October 20162 May 2019]. Available from: http://wwf.panda.org/wwf_news/?282950.
6. ACFS. Guidance on the application of Thai Agricultural Standard TAS-7401(G) - 2015 (Good Aquaculture Practices for Marine Shrimp Farm). In: The National Bureau of Agricultural Commodity and Food Standards, editor. Bangkok, Thailand2015.
7. FIS. Verifik8 Tracks, Manages and Displays Social and Environmental Data on Seafood Operations: Fish Information & Services (FIS); 2018. July 10, 2018:[Available from: https://fis.com/fis/techno/newtechno.asp?id=98239.
8. DoF. Extending of Bacillus Pormor-1 production base Department of Fisheries: Department of Fisheries; 2018 [updated May 28, 2018]. Available from: https://www4.fisheries.go.th/index.php/dof/activity_item/1695/all_activity2/4.
